# Sesamol derivatives bearing a quinazolin moiety: synthesis, antifungal efficacy against tea plant pathogens, and mechanistic insights

**DOI:** 10.1007/s13659-025-00568-x

**Published:** 2026-06-01

**Authors:** Haixia Tang, Shiyi Liu, Fali Wang, Chao Gao, Linhong Jin, Dandan Xie

**Affiliations:** 1 State Key Laboratory of Green Pesticide, Guizhou University, Guiyang, 550025 China; 2 College of Forestry, Guizhou University, Huaxi District, Guiyang, 550025 China; 3 College of Tea Science, Guizhou University, Huaxi District, Guiyang, 550025 China

**Keywords:** Fungicide, Sesamol, Quinazolin, Tea plant disease, Antifungal activity, Induced resistance

## Abstract

**Abstract:**

*Pestalotiopsis* sp. and *Colletotrichum camelliae* are two devastating fungal pathogens that cause significant yield losses and quality degradation in tea plants (*Camellia sinensis*). The increasing resistance of these pathogens to commercial fungicides necessitates the development of novel fungicidal agents with distinct modes of action. In this study, A series of novel sesamol derivatives incorporating a quinazolin moiety were synthesized and structurally validated by ^1^H NMR, ^13^C NMR, and HRMS. In vitro bioassays demonstrated that these derivatives exhibited potent antifungal activity, with compounds **4g** and **4i** standing out: against *Pestalotiopsis* sp., their inhibition rates reached 76.4% and 86.9% (vs. 18.0% for the parent compound sesamol); against *C. camelliae*, the rates were 57.9% and 70.8% (vs. 42.3% for sesamol). Notably, their in vivo control efficacy significantly surpassed that of azoxystrobin: **4g** and **4i** controlled *C. camelliae* with efficiencies of 66.6% and 51.7% (vs. 25.7% for azoxystrobin) and *Pestalotiopsis* sp. with 99.0% efficacy (vs. 57.1% for azoxystrobin). Mechanistic investigations revealed dual modes of action: (i) direct disruption of fungal cell membrane integrity; (ii) induction of tea plant resistance via up-regulating activities of defense-related enzymes (PAL, POD, SOD, CAT). Integrated transcriptomic and proteomic analyses further confirmed that **4g** activates classical defense pathways by coordinately regulating defense gene expression and protein synthesis.

In summary, these sesamol-quinazolin hybrids demonstrate potent antifungal activities with multiple targets. This study not only expands the application scope of the natural product sesamol but also provides promising lead compounds for the development of eco-friendly fungicides in tea plant protection.

**Graphical Abstract:**

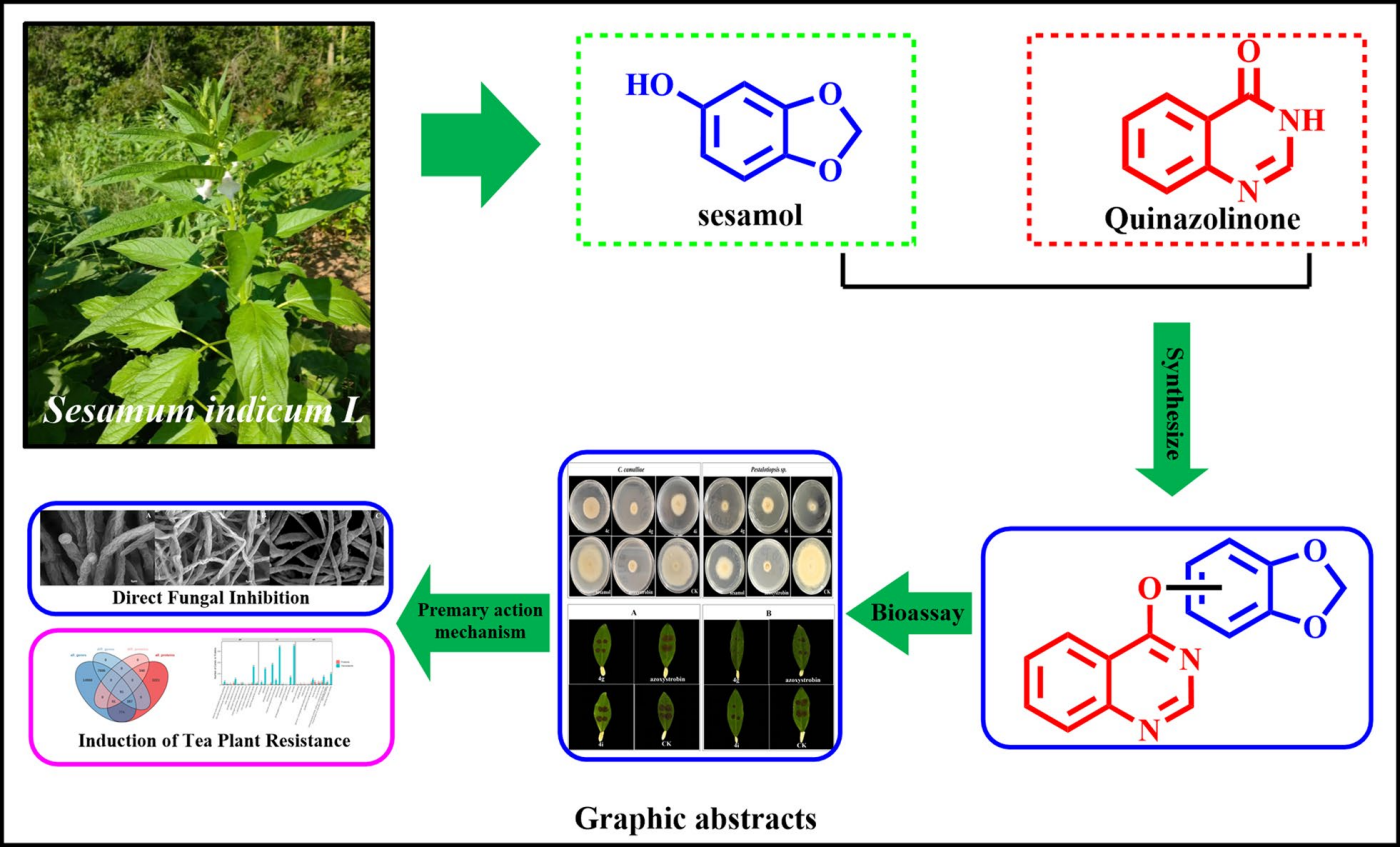

**Supplementary Information:**

The online version contains supplementary material available at 10.1007/s13659-025-00568-x.

## Introduction

Tea plant (*Camellia sinensis*) is one of the most economically valuable beverage crops globally, with its production supporting agricultural sustainability and livelihoods in major tea-producing regions [1]. However, fungal diseases pose a persistent threat to tea cultivation, with tea grey blight (caused by *Pestalotiopsis* sp.) and tea anthracnose (caused by *Colletotrichum camelliae*) being two of the most destructive [2, 3]. Infection by these pathogens leads to leaf wilting, abscission, and yield losses of 25–50%, alongside degradation of tea quality, such as reduced amino acid content and altered flavor profiles [4, 5]. Despite the use of commercial fungicides such as azoxystrobin, the rapid evolution of pathogen resistance and growing concerns over environmental toxicity have created an urgent need for novel fungicides with innovative modes of action [6, 7].

Natural products have long served as a rich source of lead compounds for pesticide development, owing to their structural diversity, low environmental impact, and evolutionary adaptation to biological targets [8, 9]. Sesamol (3,4-methylenedioxyphenol), a lignan isolated from *Sesamum indicum L*., exhibits diverse bioactivities, including antifungal and antioxidant properties [10]. However, its application in crop protection is limited by low potency, and no studies have reported its efficacy against tea plant pathogens. Structural modification of natural products–by integrating pharmacophores with proven bioactivity—is a well-established strategy to enhance efficacy [11].

Quinazolin derivatives, a class of heterocyclic compounds, have gained prominence in agrochemical research due to their broad-spectrum antimicrobial activity [12, 13]. For instance, quinazolin-based fungicides have been commercialized for controlling wheat rust and rice blast, targeting pathways such as fungal cell wall synthesis and energy metabolism [14, 15]. Inspired by the “pharmacophore fusion” strategy, we hypothesized that conjugating a quinazolin moiety to the sesamol scaffold could synergistically enhance antifungal activity against tea plant pathogens.

Herein, we report the synthesis of a series of sesamol-quinazolin derivatives, their in vitro and in vivo antifungal activity against *Pestalotiopsis* sp. And *C. camelliae*, and their mode of action (direct antifungal activity and induction of plant resistance) elucidated via morphological observation, enzyme assays, and multi-omics analysis.

## Results and discussion

### Chemistry

Intermediates 1–3 were synthesized according to previous reported method and the yield of the product was higher than 90% at each step. In the synthesis of the target compounds, we directly used commercially available K_2_CO_3_ as the catalyst for the reaction at the beginning, but the reaction time was long and the yield was only about 80%. Therefore, we ground K_2_CO_3_ into a fine powder before the reaction, resulting in a much shorter reaction time, usually less than 1 h, and the yield of more than 90%. The structures of all compounds were determined by NMR and HRMS. Usually speaking, people are most concerning about the heal effect of medicine rather than the price due to medicine are directly to treat human diseases. Pesticides are different from medicine, the people are more concerning about the cost of pesticide because of pesticides are mainly used for the control crop diseases rather than directly acting on the human body. The raw materials we adopted for synthesizing target compounds are very cheap and all intermediates have high yield. In addition, the structure of the target compound uncomplicated, which conforms to the atomic economy of pesticides.

### Antifungal activity against tea plant pathogens

**Fig. 1:**
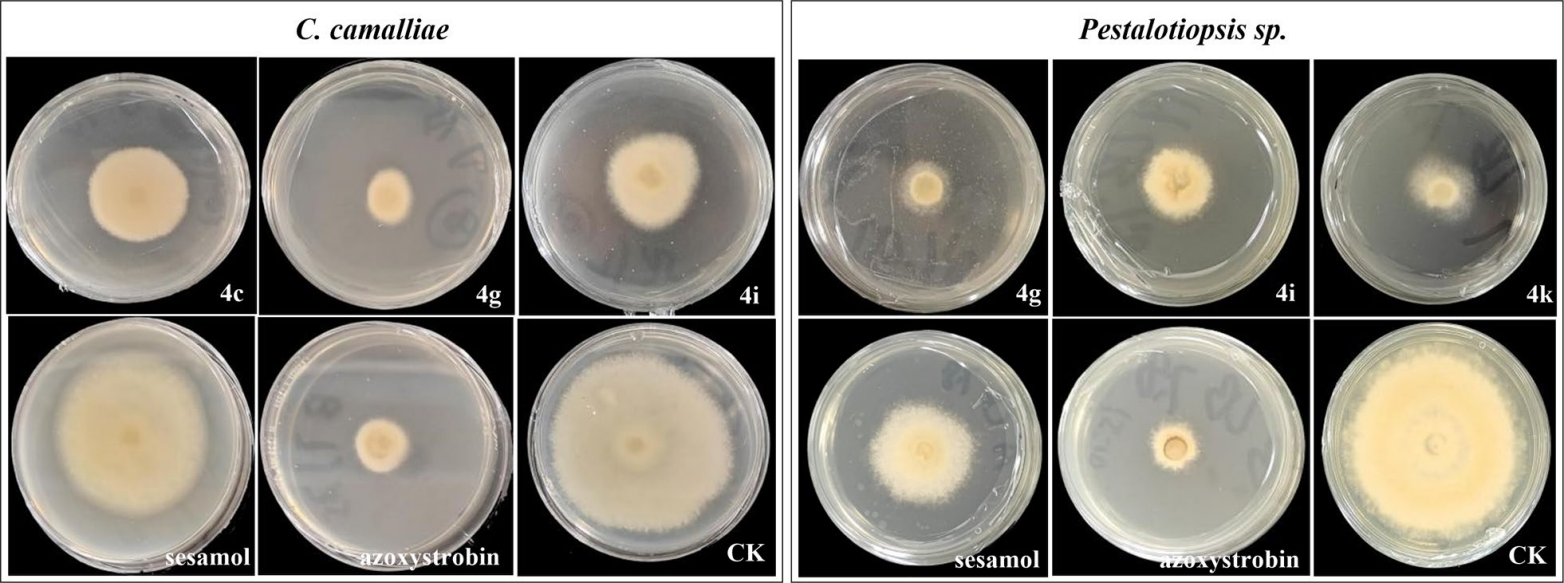
Inhibitory activity of compounds **4g**, **4c**, and **4i** against *Pestalotiopsis* sp. and C. *camalliae* in vitro

**Fig.2:**
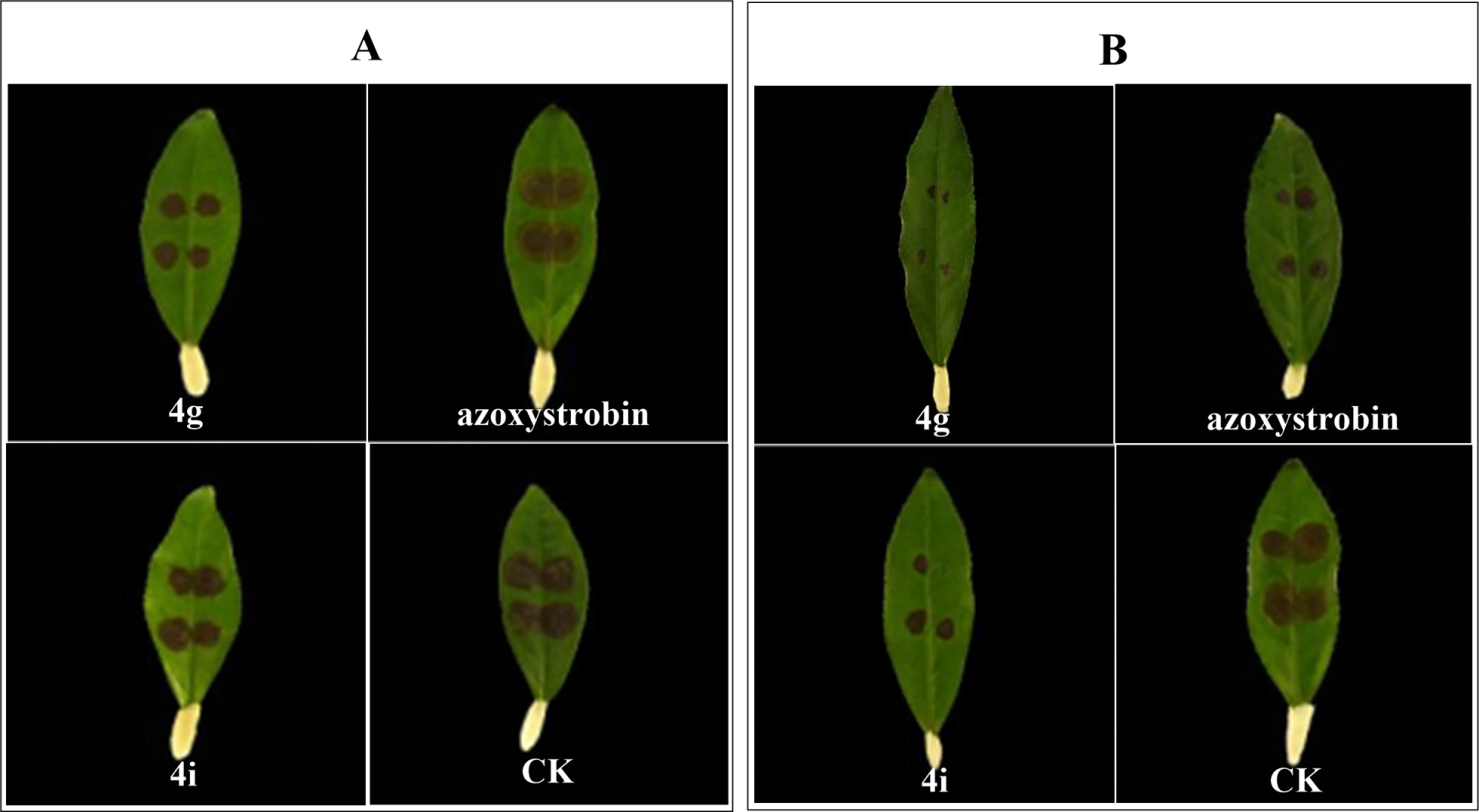
Inhibitory activity of compounds **4g** and **4i** against *Pestalotiopsis* sp. and C. *camalliae* in vivo

In vitro assays showed that most derivatives exhibited moderate to strong activity against *Pestalotiopsis* sp. And *C. camelliae* (Table 1, Fig. 1). Compounds **4g** and **4i** were the most potent: inhibition rates against *Pestalotiopsis* sp. were 76.4% and 86.9% (vs. 18.0% for sesamol), and against *C. camelliae* were 57.9% and 70.8% (vs. 42.3% for sesamol). Notably, their in vivo efficacy was superior to azoxystrobin: **4g** and **4i** controlled *C. camelliae* with efficiencies of 66.6% and 51.7% (vs. 25.7% for azoxystrobin) and *Pestalotiopsis* sp. with 99.0% efficacy (vs. 57.1% for azoxystrobin) (Table 2, Fig. 2). The higher in vivo activity suggests that these derivatives may act through both direct antifungal activity and induction of plant resistance—a characteristic that aligns with the advantages of natural product derivatives reported in previous studies [16]. Analysis of structural characteristics reveals that the high activity of Compound 4g benefits from a key factor: the retention of a hydrogen atom at the 5-position of the quinazoline ring. This structure has no additional steric hindrance, ensuring the molecule can flexibly fit into the active pocket of pathogen targets, and it also maintains an appropriate overall electron cloud distribution, providing a structural basis for stable binding with targets through hydrogen bonds and hydrophobic interactions, thereby significantly enhancing activity. Further analysis of the structure–activity relationship (SAR) based on substituent differences shows that at the 5-position, besides hydrogen, introducing halogens such as fluorine (4c), chlorine (4f), and bromine (4q) significantly reduces activity against both pathogens—for example, the inhibition rate of 4f against *Pestalotiopsis* sp. is 44.1 ± 2.5%, only 50.7% of that of 4g—confirming that the absence of substitution at the 5-position is crucial for maintaining activity; at the 6-position, the derivative with a fluorine substituent (4i, 6-F) shows relatively high activity (inhibition rate against *C. camelliae*: 57.9 ± 1.2%), while activity decreases sequentially with methyl (4a, 6-CH₃: 39.8 ± 0.8%), iodine (4h, 6-I: 39.4 ± 1.4%), and nitro (4s, 6-NO₂: 29.6 ± 0.7%) substituents, suggesting that small-sized substituents with moderate electronegativity are more suitable for this site; at the 7-position, introducing a nitro group (4l, 7-NO₂) causes a sharp drop in activity (inhibition rate against *Pestalotiopsis* sp. is only 20.3 ± 2.3%), and derivatives with chlorine (4t, 7-Cl: 36.8 ± 2.1%) and bromine (4n, 7-Br: 20.3 ± 2.3%) substituents also exhibit moderate activity, indicating that strong electron-withdrawing groups disrupt the active conformation; at the 8-position, derivatives with methyl (4d, 8-CH₃) and bromine (4j, 8-Br) substituents show little difference in activity (inhibition rates against *Pestalotiopsis* sp. are 49.6 ± 1.4% and 78.8 ± 2.3%, respectively), showing that this position has a limited regulatory effect on overall activity. In conclusion, the antifungal activity of sesamol-quinazoline derivatives is closely related to the position, type, and physicochemical properties of substituents on the quinazoline ring, and among these, "no substitution at the 5-position and modification with small-sized electronegative groups at the 6-position" are important structural features enabling such compounds to exhibit high activity against tea plant pathogens.

**Table 1:** In vitro inhibition rate (%) of target compounds 4a-4t against *Pestalotiopsis* sp. and *C. camelliae* at 100 µg/mL

Comp		Inhibition rate ± SD (%)	
		*C. camalliae*	*Pestalotiopsis* sp.
**4a**	6-CH_3_	39.8 ± 0.8	56.4 ± 1.1
**4b**	6-Cl	12.5 ± 1.5	31.8 ± 1.4
**4c**	5-F	46.8 ± 0.3	36.9 ± 0.6
**4d**	8-CH_3_	13.0 ± 1.4	49.6 ± 1.4
**4e**	6,7-OCH_3_	12.5 ± 0.3	33.1 ± 2.0
**4f**	5-Cl	21.3 ± 2.1	44.1 ± 2.5
**4g**	5-H	76.4 ± 1.1	86.9 ± 0.6
**4h**	6-I	39.4 ± 1.4	39.4 ± 1.1
**4i**	6-F	57.9 ± 1.2	70.8 ± 0.9
**4j**	8-Br	29.2 ± 1.3	78.8 ± 2.3
**4k**	7-F	32.9 ± 1.2	72.5 ± 0.6
**4l**	7-NO_2_	–	20.3 ± 2.3
**4m**	6-Cl, 8-CH_3_	32.9 ± 0.8	–
**4n**	7-Br	27.3 ± 1.1	20.3 ± 2.3
**4o**	6-Br	–	–
**4p**	6,8-Cl	25.0 ± 0	18.2 ± 2.3
**4q**	5-Br	39.4 ± 2.3	67.0 ± 1.7
**4r**	6-OCH_3_	44.9 ± 0.5	31.8 ± 2.0
**4s**	6-NO_2_	29.6 ± 0.7	66.5 ± 2.3
**4t**	7-Cl	36.8 ± 2.1	–
**Sesamol**		18.0 ± 2.4	42.3 ± 2.2
**Azoxystrobin**		70.8 ± 1.7	84.9 ± 1.0

**Table 2:** Inhibition effect of compounds against *C. camalliae* and *Pestalotiopsis* sp. at 100 μg/mL in vivo

Comp	*C. camalliae*	*Pestalotiopsis* sp*.*
4 g	66.6 ± 4.1	99.0 ± 1.0
4i	51.7 ± 5.4	81.5 ± 5.0
azoxystrobin	25.7 ± 3.5	57.1 ± 2.9

### Broad-spectrum antifungal activity

**Fig. 3:**
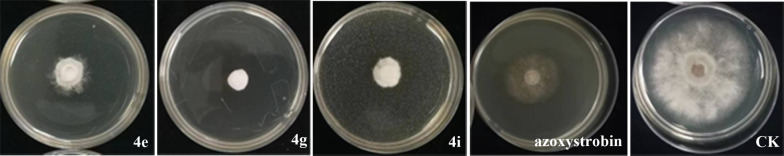
Inhibitory activity of compounds **4e**, **4g**, and **4i** against *Botrytis cinerea Pers.* Fr. in vitro

Derivatives also exhibited activity against other agricultural pathogens (Table 3, Fig. 3). All compounds inhibited *B. cinerea* with rates > 60% (vs. 56.0% for azoxystrobin), indicating potential for controlling gray mold in fruits and vegetables. However, most derivatives were less active than azoxystrobin against *S. sclerotiorum* and *P. capsici*, suggesting that structural optimization is needed to broaden their spectrum of activity.

**Table 3:** Inhibition effect of target compounds against fungi at 100 μg/mL in vitro

Comp		Inhibition rate ± SD (%)			
		*Phomopsis* sp.	*Sclerotinia sclerotiorum*	*Phytophthora capsici Leonian*	*Botrytis cinerea Pers. Fr*
**4a**	6-CH_3_	46.9 ± 0.6	49.4 ± 2.0	11.29 ± 1.2	73.66 ± 1.9
**4b**	6-Cl	24.5 ± 1.3	51.3 ± 1.3	30.1 ± 2.3	68.3 ± 0.7
**4c**	5-F	69.7 ± 1.7	59.2 ± 0.6	37.6 ± 2.3	81.9 ± 0.7
**4d**	8-CH_3_	27.8 ± 1.7	43.1 ± 1.7	15.9 ± 1.8	50.6 ± 1.2
**4e**	6,7-OCH_3_	42.7 ± 0.4	57.3 ± 0.0	29.7 ± 0.5	69.1 ± 1.2
**4f**	5-Cl	33.2 ± 0.6	39.0 ± 0.6	13.8 ± 1.2	70.8 ± 0.7
**4 g**	H	80.5 ± 2.0	92.5 ± 1.7	54.0 ± 2.2	92.6 ± 0.2
**4 h**	6-I	36.9 ± 0.6	62.6 ± 0.6	12.6 ± 0.1	72.8 ± 0.2
**4i**	6-F	63.5 ± 1.3	65.9 ± 1.3	15.5 ± 1.5	87.6 ± 0.2
**4j**	8-Br	65.2 ± 4.2	56.2 ± 1.1	16.7 ± 0.7	74.1 ± 1.2
**Azoxystrobin**		67.6 ± 0.2	74.5 ± 2.3	50.2 ± 1.0	56.0 ± 0.7

### Mode of action: direct fungal inhibition

**Fig. 4:**
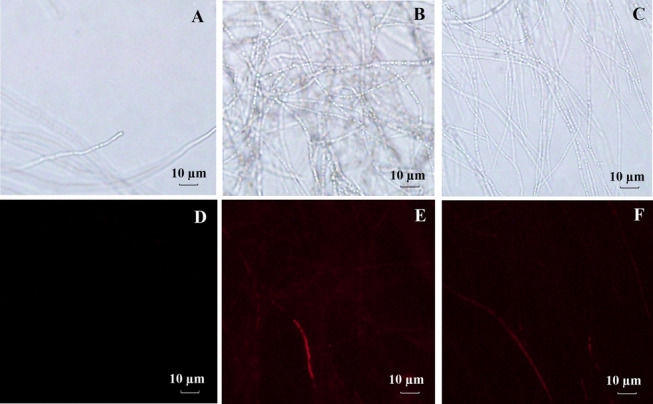
Staining of mycelium by target compounds **4g**, **4i** after treatment at 100 μg/mL

**Fig. 5:**
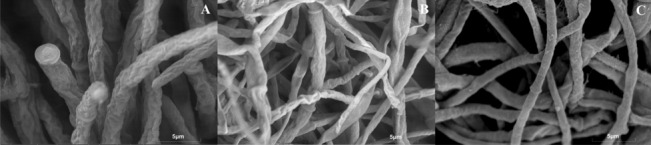
SEM observations of *Pestalotiopsis* sp. cell morphological changes post-treatment with 100 μg/mL concentrations of compounds **4g**、**4i**

Fluorescence Microscope (FM) analysis showed that **4g**- and **4i**-treated *Pestalotiopsis* sp. mycelia emitted strong red fluorescence after PI (pyridinium iodide) staining, indicating disrupted cell membrane integrity (Fig. 4). SEM revealed that treated mycelia were shriveled with collapsed walls and irregular surfaces, whereas control mycelia were smooth and intact (Fig. 5). These results confirm that the derivatives act by damaging fungal cell membranes—a mode of action shared by many effective antifungals targeting membrane lipids or proteins [17].

### Mode of action: induction of tea plant resistance

**Fig. 6:**
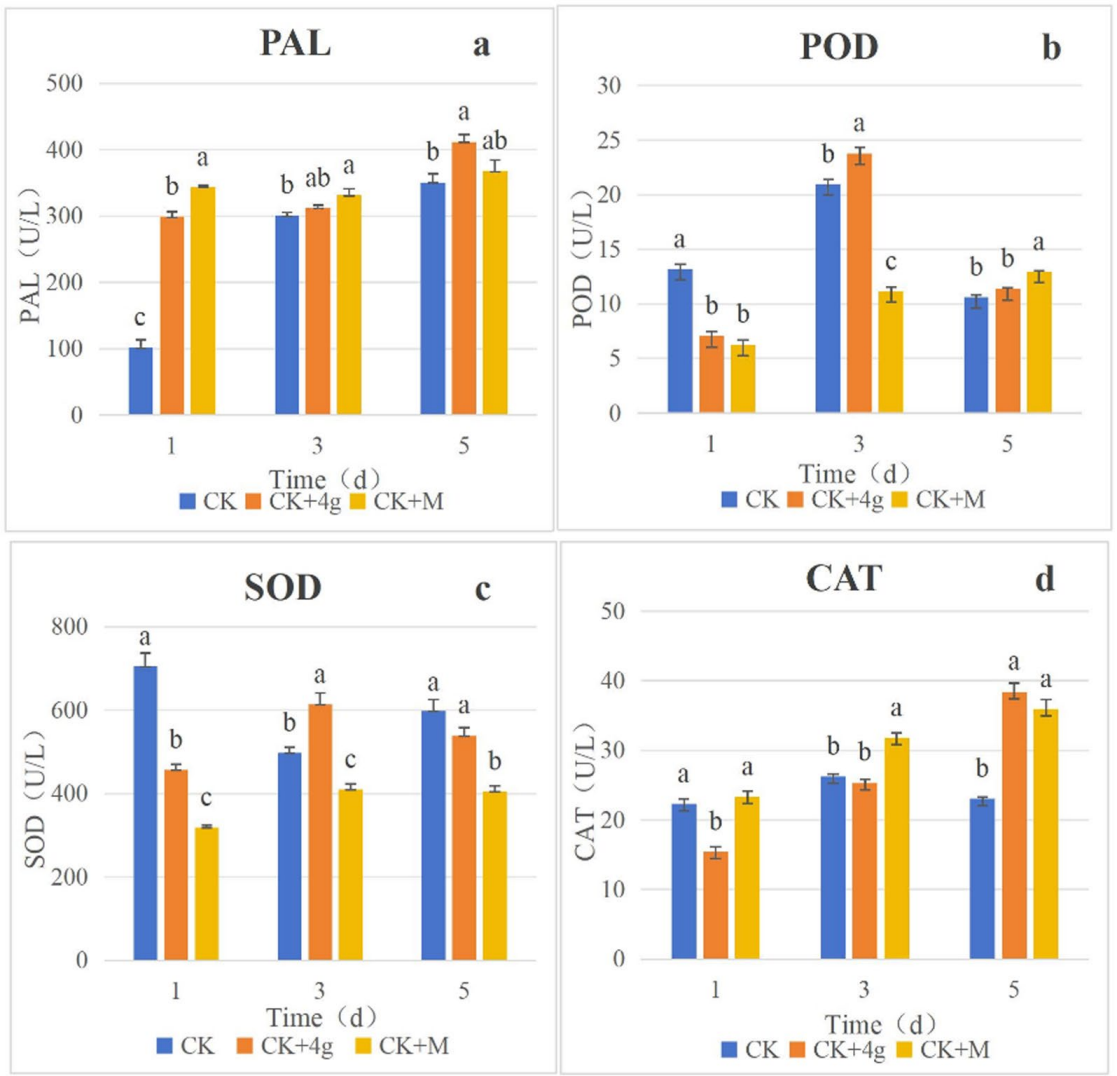
Effect of compound **4 g** on defence enzyme in tea plant

Defense enzyme assays showed that 4g enhanced SOD (1.23-fold), CAT (1.67-fold), POD (1.13-fold), and PAL (1.2-fold) activities in tea leaves (Fig. 6). SOD and CAT scavenge reactive oxygen species (ROS) to maintain redox homeostasis, while PAL and POD promote lignin synthesis and cell wall reinforcement [18]. This indicates that **4g** induces systemic resistance, complementing its direct antifungal activity.

**Fig. 7:**
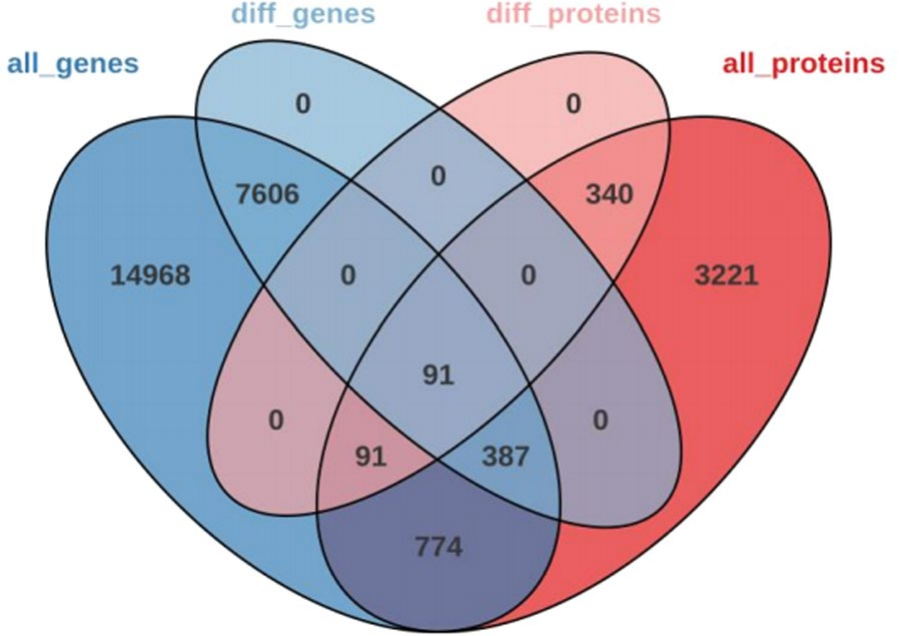
Venn diagram of DEPs and DEGs

**Fig. 8:**
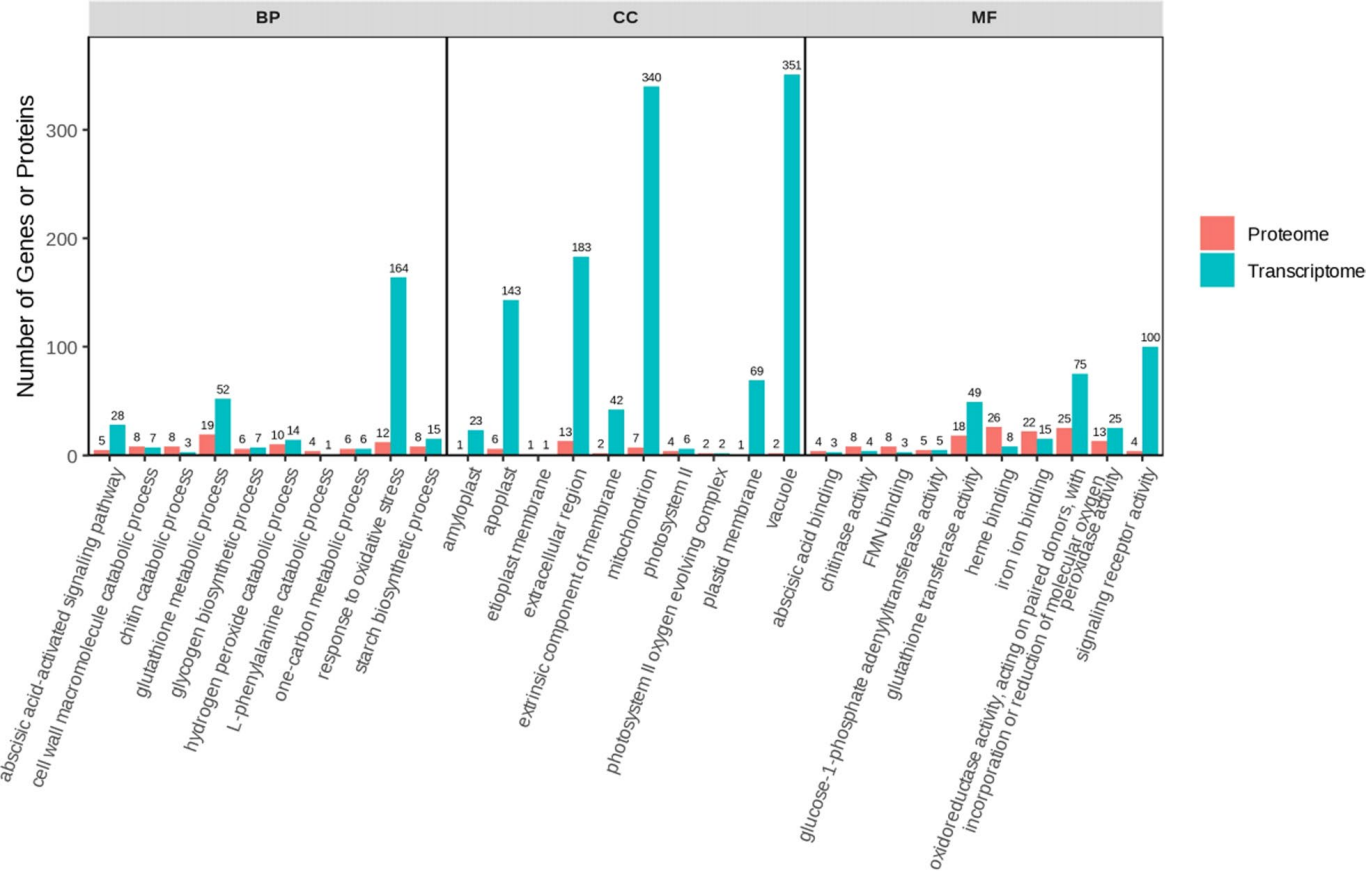
Bar chart of GO enrichment analysis

**Fig. 9:**
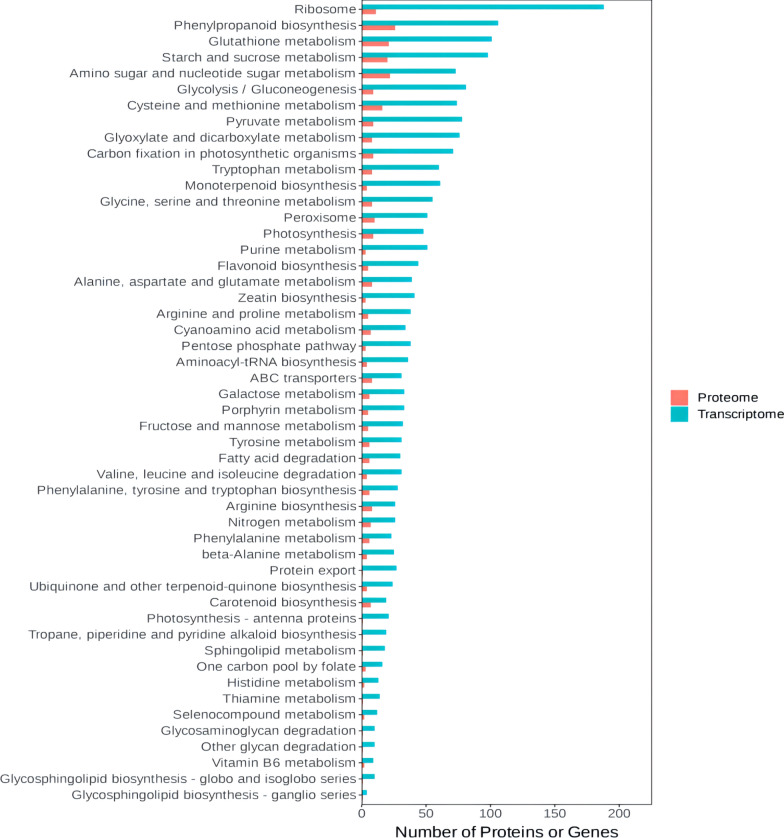
Bar chart of KEGG enrichment analysis

We had the effects of compound **4 g** on genes and proteins in tea plants by transcriptome and proteomics analysis (Figs. S1, S2, S3, S5, S6, S7 in Supplementary Data). Multi-omics analysis identified 91 common DEGs and DEPs involved in phenylpropanoid metabolism, glutathione metabolism, and oxidative stress response (Figs. 7, 8, 9). For example, PAL and 4CL (key enzymes in phenylpropanoid biosynthesis) were upregulated, promoting lignin and flavonoid synthesis (physical and chemical barriers). Antioxidant enzymes (GPX, GR) and ROS-scavenging proteins were also upregulated, consistent with enzyme activity data. Correlation analysis showed that Cu/Zn-SOD and CAT expression positively correlated with their respective enzyme activities (r = 0.89 and 0.82), confirming coordinated regulation of resistance at the transcriptional and translational levels.

## Conclution

In this study, we synthesized a series of sesamol-quinazolin derivatives and demonstrated their potent in vitro and in vivo antifungal activity against *Pestalotiopsis* sp. And *C. camelliae*. Mechanistic studies revealed that these derivatives act through dual modes of action: direct disruption of fungal cell membranes and induction of tea plant resistance via activation of defense enzymes and key metabolic pathways. Given their strong efficacy, clear mechanisms, and simple synthesis, these derivatives are promising leads for the development of novel fungicides. Future work will focus on structural optimization to enhance broad-spectrum activity and field efficacy evaluation.

## Methods and materials

### Chemicals and instruments

All starting materials and reagents were commercially available and used without further purification. The ^1^H NMR and ^13^C NMR spectra were recorded on a Bruker DPX 400 MHz (Bruker BioSpin GmbH, Rheinstetten, Germany) NMR spectrometer with CDCl_3_ as the solvent. The following abbreviations were used to explain the multiplicities: s, singlet; d, doublet; t, triplet; m, multiplet; and br, broadened. The melting points were determined on a WRX–4 microscope melting point apparatus (YiCe Apparatus & Equipment co., LTD, Shanghai, China). High-resolution mass spectrometry (HRMS) was conducted using a Thermo Scientific Q Exactive (Thermo Fisher Scientific, Massachusetts, America).

### General procedure for the synthesis of target compounds (4a–4t)

**Fig. 10:**
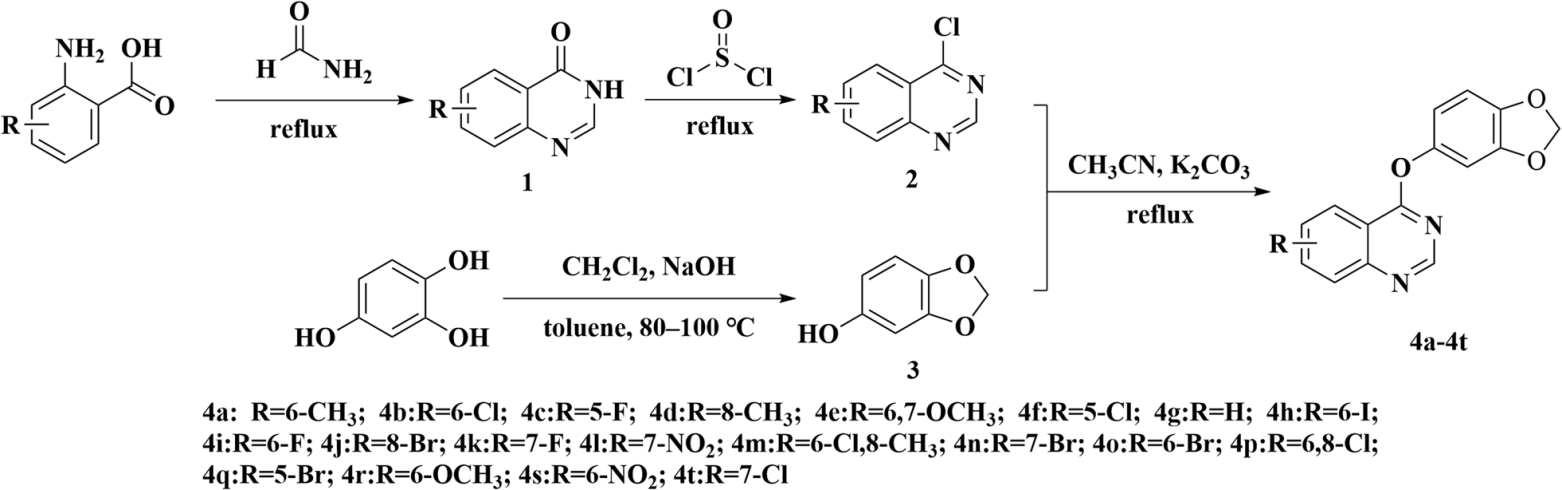
Synthetic route of the title compounds **4a–4t**

The synthetic route is outlined in Scheme 1 (Fig. 10). Intermediates 1–3 were prepared according to previously reported methods with minor modifications [19–22]^.^ Briefly, intermediate **2** (6 mmol), intermediate **3** (6 mmol), and anhydrous K₂CO₃ (12 mmol) were added to a 250 mL round-bottom flask containing acetonitrile (100 mL). The mixture was stirred under reflux at 80 °C and monitored by TLC (eluent: ethyl acetate/petroleum ether = 1:3, v/v). Upon completion (4–6 h), the solvent was removed under reduced pressure (40 °C, rotary evaporator). The crude product was purified by silica gel column chromatography (eluent: ethyl acetate/petroleum ether = 3:1, v/v) to afford compounds **4a–4t** as white or light yellow solids. Target compounds were structurally confirmed by ^1^H NMR, ^13^C NMR, and HRMS.

#### 4-(benzo[d][1,3]dioxol-5-yloxy)-6-methylquinazoline (4a)

White solid;m.p. 173.6 ~ 174.4 °C; yield, 93.64%.^1^H NMR (400 MHz, CDCl_3_) δ 8.73 (s, 1H), 8.12 (d, *J* = 1.0 Hz, 1H), 7.90 (d, *J* = 8.6 Hz, 1H), 7.73 (dd, *J* = 8.6, 2.0 Hz, 1H), 6.87 (d, *J* = 8.3 Hz, 1H), 6.77 (d, *J* = 2.3 Hz, 1H), 6.70 (dd, *J* = 8.3, 2.4 Hz, 1H), 6.03 (s, 2H), 2.59 (s, 3H).^13^C NMR (100 MHz, CDCl_3_) *δ* 166.8, 153.5, 150.1, 148.3, 146.7, 145.5, 137.8, 136.2, 127.6, 122.4, 116.2, 114.2, 108.3, 104.1, 101.9, 21.8. HRMS (ESI): calculated for C_16_H_12_N_2_O_3_ [M + H]^+^: 281.092069, found: 218.09237.

#### 4-(benzo[d][1,3]dioxol-5-yloxy)-6-chloroquinazoline(4b)

White solid; m.p. 138.1 ~ 139.7 °C; yield, 94.63%. ^1^H NMR (400 MHz, CDCl_3_) *δ* 8.78 (s, 1H), 8.29 (d, *J* = 8.8 Hz, 1H), 8.00 (d, *J* = 2.0 Hz, 1H), 7.61 (dd, *J* = 8.8, 2.0 Hz, 1H), 6.88 (d, *J* = 8.4 Hz, 1H), 6.76 (d, *J* = 2.3 Hz, 1H), 6.70 (dd, *J* = 8.4, 2.4 Hz, 1H), 6.04 (s, 2H). ^13^C NMR (100 MHz, CDCl_3_) *δ* 167.2, 155.4, 152.3, 148.4, 146.3, 145.7, 140.5, 128.7, 127.2, 125.1, 114.8, 114.12, 108.4, 103.9, 102.0. HRMS (ESI): calculated for C_15_H_9_ClN_2_O_3_ [M + H]^+^: 301.037446, found: 301.03756.

#### 4-(benzo[d][1,3]dioxol-5-yloxy)-5-fluoroquinazoline(4c)

White solid; m.p. 140.3 ~ 141.9 °C; yield, 74.76%.^1^H NMR (400 MHz, CDCl_3_) *δ* 8.75 (s, 1H), 7.89–7.79 (m, 2H), 7.34 –7.29 (m, 1H), 6.88 (d, *J* = 8.3 Hz, 1H), 6.77 (d, *J* = 2.4 Hz, 1H), 6.71 (dd, *J* = 8.3, 2.4 Hz, 1H), 6.04 (s, 2H). ^13^C NMR (100 MHz, CDCl_3_) *δ* 166.25 (d, ^3^*J*_*C-F*_ = 5.1 Hz), 158.0 (d, ^1^*J*_*C-F*_ = 264.3 Hz), 154.8 (d, ^4^*J*_*C-F*_ = 2.0 Hz), 153.3, 148.3, 146.5, 145.6, 134.2, 134.1, 123.9 (d, ^4^*J*_*C-F*_ = 4.6 Hz), 114.2, 113.2 (d, ^2^*J*_*C-F*_ = 21.1 Hz), 108.3, 104.0, 101.9. ^19^F NMR (377 MHz, CDCl_3_) *δ*-106.6. HRMS (ESI): calculated for C_15_H_9_FN_2_O_3_ [M + H]^+^: 285.066997, found: 285.06713.

#### 4-(benzo[d][1,3]dioxol-5-yloxy)-8-methylquinazoline(4d)

Yellow solid;m.p. 146.7 ~ 147.3 °C; yield, 42.12%.^1^H NMR (400 MHz, CDCl_3_) *δ* 8.84 (s, 1H), 8.20 (d, *J* = 8.1 Hz, 1H), 7.75 (d, *J* = 7.0 Hz, 1H), 7.54 (dd, *J* = 8.3, 7.2 Hz, 1H), 6.88 (d, *J* = 8.3 Hz, 1H), 6.77 (d, *J* = 2.3 Hz, 1H), 6.70 (dd, *J* = 8.3, 2.4 Hz, 1H), 6.04 (s, 2H), 2.77 (s, 3H). ^13^C NMR (100 MHz, CDCl_3_) *δ* 167.5, 153.3, 150.8, 148.3, 146.7, 145.5, 136.3, 134.2, 127.1, 121.2, 116.3, 114.2, 108.3, 104.1, 101.9, 17.7.HRMS (ESI): calculated for C_16_H_12_N_2_O_3_ [M + H]^+^: 281.092069, found: 281.09273.

#### 4-(benzo[d][1,3]dioxol-5-yloxy)-6,7-dimethoxyquinazoline.(4e)

White solid; m.p. 199.6 ~ 200.6 °C; yield, 31.44%.^1^H NMR (400 MHz, CDCl_3_) *δ* 8.65 (s, 1H), 7.53 (s, 1H), 7.32 (s, 1H), 6.88 (d, *J* = 8.3 Hz, 1H), 6.77 (d, *J* = 2.3 Hz, 1H), 6.71 (dd, *J* = 8.4, 2.4 Hz, 1H), 6.04 (s, 2H), 4.07 (s, 3H), 4.06 (s, 3H). ^13^C NMR (100 MHz, CDCl_3_) *δ* 165.7, 155.9, 153.0, 150.2, 149.3, 148.3, 146.8, 145.5, 114.2, 110.6, 108.3, 106.8, 104.1, 101.9, 101.0, 56.4, 56.4.HRMS (ESI): calculated for C_17_H_14_N_2_O_5_ [M + H]^+^: 327.097548, found: 327.09868.

#### 4-(benzo[d][1,3]dioxol-5-yloxy)-5-chloroquinazoline(4f)

Yellow solid;m.p. 145.1 ~ 146.1 °C; yield, 64.79%. ^1^H NMR (400 MHz, CDCl_3_) *δ* 8.78 (s, 1H), 8.29 (d, *J* = 8.8 Hz,1H), 7.99 (d, *J* = 2.0 Hz, 1H), 7.61 (dd, *J* = 8.8, 2.0 Hz, 1H), 6.88 (d, *J* = 8.4 Hz, 1H), 6.76 (d, *J* = 2.4 Hz, 1H), 6.70 (dd, *J* = 8.3, 2.4 Hz, 1H), 6.04 (s, 2H). ^13^C NMR (100 MHz, CDCl_3_) *δ* 167.2, 155.4, 152.3, 148.4, 146.3, 145.7, 140.5, 128.6, 127.2, 125.1, 114.8, 114.1, 108.3, 103.9, 101.9.HRMS (ESI): calculated for C_15_H_9_ClN_2_O_3_ [M + H]^+^: 301.037446, found: 301.03741.

#### 4-(benzo[d][1,3]dioxol-5-yloxy)quinazoline(4g)

White solid; m.p.147.8 ~ 148.2 °C; yield, 85.43%.^1^H NMR (400 MHz, CDCl_3_) *δ* 8.79 (s, 1H), 8.45–8.32 (m, 1H), 8.12–7.97 (m, 1H), 7.93–7.89 (m, 1H), 7.68–7.64 (m, 1H), 6.88 (d, *J* = 8.4 Hz, 1H), 6.78 (d, *J* = 2.4 Hz, 1H), 6.71 (dd, *J* = 8.3, 2.4 Hz, 1H), 6.04 (s, 2H). ^13^C NMR (100 MHz, CDCl_3_) *δ* 167.2, 154.3, 151.6, 148.4, 146.6, 145.6, 134.2, 127.9, 127.6, 123.6, 116.4, 114.2, 108.3, 104.0, 101.9. HRMS (ESI): calculated for C_15_H_10_N_2_O_3_ [M + H]^+^: 267.076419, found: 267.07674.

#### 4-(benzo[d][1,3]dioxol-5-yloxy)-6-iodoquinazoline(4h)

Yellow solid;m.p. 153.7 ~ 154.9 °C; yield, 79.92%.^1^H NMR (400 MHz, CDCl_3_) *δ* 8.79 (s, 1H), 8.73 (d, *J* = 2.0 Hz, 1H), 8.15 (dd, *J* = 8.8, 2.0 Hz, 1H), 7.73 (d, *J* = 8.8 Hz, 1H), 6.88 (d, *J* = 8.4 Hz, 1H), 6.75 (d, *J* = 2.4 Hz, 1H), 6.69 (dd, *J* = 8.4, 2.4 Hz, 1H), 6.05 (s, 2H). ^13^C NMR (100 MHz, CDCl_3_) *δ* 165.9, 154.6, 150.7, 148.4, 146.3, 145.8, 142.9, 132.6, 129.6, 117.9, 114.1, 108.4, 103.9, 102.0, 92.5.HRMS (ESI): calculated for C_15_H_9_IN_2_O_3_ [M + H]^+^: 392.973062, found: 392.97322.

#### 4-(benzo[d][1,3]dioxol-5-yloxy)-6-fluoroquinazoline(4i)

Yellow solid;m.p. 141.9 ~ 143.0 °C; yield, 74.94%. ^1^H NMR (400 MHz, CDCl_3_) *δ* 8.77 (s, 1H), 8.02 (dd, *J* = 9.2, 5.0 Hz, 1H), 7.95 (dd, *J* = 8.3, 2.8 Hz, 1H), 7.70–7.65 (m, 1H), 6.88 (d, *J* = 8.3 Hz, 1H), 6.77 (d, *J* = 2.4 Hz, 1H), 6.70 (dd, *J* = 8.3, 2.4 Hz, 1H), 6.05 (s, 2H). ^13^C NMR (100 MHz, CDCl_3_) *δ* 166.9 (d, ^4^*J*_*C-F*_ = 5.1 Hz), 160.7 (d, ^1^*J*_*C-F*_ = 250.6 Hz), 153.6 (d, ^4^*J*_*C-F*_ = 2.6 Hz), 148.7, 148.4, 146.4, 145.7, 130.6 (d, ^3^*J*_*C-F*_ = 8.7 Hz), 124.2 (d, ^2^*J*_*C-F*_ = 25.3 Hz), 117.0 (d, ^3^*J*_*C-F*_ = 9.7 Hz), 114.1, 108.3, 107.6 (d, ^2^*J*_*C-F*_ = 23.4 Hz), 103.9, 101.9. ^19^F NMR (377 MHz, CDCl_3_) *δ*-109.95. HRMS (ESI): calculated for C_15_H_9_FN_2_O_3_ [M + H]^+^:285.066997, found: 285.06739.

#### 4-(benzo[d][1,3]dioxol-5-yloxy)-8-bromoquinazoline(4j)

Yellow solid;m.p. 142.6 ~ 143.5 °C; yield, 64.13%. ^1^H NMR (400 MHz, CDCl_3_) *δ* 8.92 (s, 1H), 8.34 (dd, *J* = 8.2, 1.3 Hz, 1H), 8.21 (dd, *J* = 7.6, 1.4 Hz, 1H), 7.52 (t, *J* = 7.9 Hz, 1H), 6.88 (d, *J* = 8.4 Hz, 1H), 6.76 (d, *J* = 2.4 Hz, 1H), 6.70 (dd, *J* = 8.3, 2.4 Hz, 1H), 6.04 (s, 2H). ^13^C NMR (100 MHz, CDCl_3_) *δ* 167.5, 155.0, 149.2, 148.4, 146.4, 145.8, 137.7, 128.0, 123.3, 123.2, 117.7, 114.1, 108.3, 103.9, 102.0. HRMS (ESI): calculated for C_15_H_9_BrN_2_O_3_ [M + H]^+^:344.986932, found: 344.98701.

#### 4-(benzo[d][1,3]dioxol-5-yloxy)-7-fluoroquinazoline(4k)

Yellow solid;m.p. 132.7 ~ 133.2 °C; yield, 38.35%. ^1^H NMR (400 MHz, CDCl_3_) *δ* 8.78 (s, 1H), 8.39 (dd, *J* = 9.1, 5.9 Hz, 1H), 7.64 (dd, *J* = 9.6, 2.5 Hz, 1H), 7.42 (td, *J* = 8.8, 2.5 Hz, 1H), 6.88 (d, *J* = 8.4 Hz, 1H), 6.76 (d, *J* = 2.3 Hz, 1H), 6.70 (dd, *J* = 8.4, 2.4 Hz, 1H), 6.04 (s, 2H). ^13^C NMR (100 MHz, CDCl_3_) *δ* 167.4, 164.8, 155.5, 151.4, 148.4, 146.3, 145.8, 126.6, 118.0, 114.1, 112.2, 108.4, 104.0, 102.0, 101.1. HRMS (ESI): calculated for C_15_H_9_FN_2_O_3_ [M + H]^+^:285.066997, found: 285.06725.

#### 4-(benzo[d][1,3]dioxol-5-yloxy)-7-nitroquinazoline(4l)

Yellow solid;m.p. 188.6 ~ 190.0 °C; yield, 10.40%. 1H NMR (400 MHz, CDCl_3_) *δ* 9.29 (d, *J* = 2.6 Hz, 1H), 8.90 (s, 1H), 8.68 (dd, *J* = 9.2, 2.6 Hz, 1H), 8.14 (d, *J* = 9.2 Hz, 1H), 6.90 (d, *J* = 8.3 Hz, 1H), 6.78 (d, *J* = 2.4 Hz, 1H), 6.68 (s, 1H), 6.07 (s, 2H). HRMS (ESI): calculated for C_15_H_9_N_3_O_5_ [M + H] + :312.061497, found. 312.05995.

#### 4-(benzo[d][1,3]dioxol-5-yloxy)-6-chloro-8-methylquinazoline(4m)

White solid; m.p. 205.1 ~ 205.7 °C; yield, 65.42%.^1^H NMR (400 MHz, CDCl_3_) *δ* 8.81 (s, 1H), 8.17 (d, *J* = 2.4 Hz, 1H), 7.80–7.59 (m, 1H), 6.87 (d, *J* = 8.3 Hz, 1H), 6.75 (d, *J* = 2.3 Hz, 1H), 6.69 (dd, *J* = 8.4, 2.4 Hz, 1H), 6.04 (s, 2H), 2.74 (s, 3H). ^13^C NMR (100 MHz, CDCl_3_) *δ* 166.7, 153.4, 149.3, 148.4, 146.4, 145.7, 138.8, 134.8, 132.7, 120.2, 116.9, 114.1, 108.3, 103.9, 101.9, 17.5. HRMS (ESI): calculated for C_16_H_11_ClN_2_O_3_ [M + H]^+^:315.053096, found: 315.05293.

#### 4-(benzo[d][1,3]dioxol-5-yloxy)-7-bromoquinazoline(4n)

Yellow solid;m.p. 137.5 ~ 138.0 °C; yield, 63.13%.^1^H NMR (400 MHz, CDCl_3_) *δ* 8.78 (s, 1H), 8.29–8.07 (m, 2H), 7.76 (dd, *J* = 8.7, 1.9 Hz, 1H), 6.88 (d, *J* = 8.3 Hz, 1H), 6.76 (d, *J* = 2.3 Hz, 1H), 6.69 (dd, *J* = 8.3, 2.4 Hz, 1H), 6.04 (s, 2H).^13^C NMR (100 MHz, CDCl_3_) *δ* 167.3, 155.3, 152.4, 148.4, 146.3, 145.7, 131.3, 130.5 129.0, 125.0, 115.1, 114.1, 108.4, 103.9, 102.0. HRMS (ESI): calculated for C_15_H_9_BrN_2_O_3_ [M + H]^+^:344.986932, found: 344.98701.

#### 4-(benzo[d][1,3]dioxol-5-yloxy)-6-bromoquinazoline(4o)

White solid; m.p. 170.2 ~ 170.6 °C; yield, 61.69%.^1^H NMR (400 MHz, CDCl_3_) *δ* 8.79 (s, 1H), 8.51 (d, *J* = 2.2 Hz, 1H), 7.98 (dd, *J* = 8.9, 2.2 Hz, 1H), 7.88 (d, *J* = 8.9 Hz, 1H), 6.88 (d, *J* = 8.3 Hz, 1H), 6.76 (d, *J* = 2.4 Hz, 1H), 6.70 (dd, *J* = 8.4, 2.4 Hz, 1H), 6.05 (s, 2H).^13^C NMR (100 MHz, CDCl_3_) *δ* 166.3, 154.6, 150.3, 148.4, 146.3, 145.8, 137.6, 129.7, 126.1, 121.2, 117.5, 114.1, 108.4, 103.9, 102.0. HRMS (ESI): calculated for C_15_H_9_BrN_2_O_3_ [M + H]^+^:344.986932, found: 344.98679.

#### 4-(benzo[d][1,3]dioxol-5-yloxy)-6,8-dichloroquinazoline(4p)

Yellow solid; m.p. 211.2 ~ 211.9 °C; yield, 73.21%. ^1^H NMR (400 MHz, CDCl_3_) *δ* 8.89 (s, 1H), 8.28 (d, *J* = 2.3 Hz, 1H), 7.99 (d, *J* = 2.3 Hz, 1H), 6.88 (d, *J* = 8.4 Hz, 1H), 6.75 (d, *J* = 2.3 Hz, 1H), 6.69 (dd, *J* = 8.4, 2.4 Hz, 1H), 6.05 (s, 2H). ^13^C NMR (100 MHz, CDCl_3_) *δ* 166.7, 155.0, 148.4, 147.0, 146.1, 145.9, 134.6, 133.8, 132.9, 121.7, 117.9, 114.0, 108.4, 103.8, 102.0. HRMS (ESI): calculated for C_15_H_8_Cl_2_N_2_O_3_ [M + H]^+^:334.998474, found: 334.99843.

#### 4-(benzo[d][1,3]dioxol-5-yloxy)-5-bromoquinazoline(4q)

Yellow solid;m.p. 130.8 ~ 131.3 °C; yield, 51.16%. ^1^H NMR (400 MHz, CDCl_3_) *δ* 8.74 (s, 1H), 8.05–7.85 (m, 2H), 7.67 (t, *J* = 8.0 Hz, 1H), 6.88 (d, *J* = 8.3 Hz, 1H), 6.79 (d, *J* = 2.3 Hz, 1H), 6.72 (dd, *J* = 8.4, 2.4 Hz, 1H), 6.04 (s, 2H). ^13^C NMR (100 MHz, CDCl_3_) *δ* 166.1, 154.1, 153.7, 148.4, 146.0, 145.6, 134.2, 133.8, 128.0, 117.4, 116.2, 114.2, 108.3, 104.0, 101.9.

#### 4-(benzo[d][1,3]dioxol-5-yloxy)-6-methoxyquinazoline(4r)

Brown solid;m.p. 166.7 ~ 167.6 °C; yield, 41.64%.^1^H NMR (400 MHz, CDCl_3_) *δ* 8.68 (s, 1H), 7.91 (d, *J* = 9.0 Hz, 1H), 7.62–7.47 (m, 2H), 6.88 (d, *J* = 8.3 Hz, 1H), 6.78 (d, *J* = 2.4 Hz, 1H), 6.74–6.68 (m, 1H), 6.04 (s, 2H), 3.98 (s, 3H).^13^C NMR (100 MHz, CDCl_3_) *δ* 166.3, 158.6, 152.1, 148.4, 147.5, 146.7, 145.6, 129.4, 126.6, 117.0, 114.2, 108.3, 104.1, 101.9, 101.0, 55.9.

#### 4-(benzo[d][1,3]dioxol-5-yloxy)-6-nitroquinazoline(4s)

Yellow solid;m.p. 196.4 ~ 197.1 °C; yield, 14.22%. ^1^H NMR (400 MHz, CDCl_3_) *δ* 8.86 (s, 1H), 8.19 (dd, J = 8.5, 1.1 Hz, 1H), 7.96 (dd, J = 8.5, 7.6 Hz, 1H), 7.78–7.74 (m, 1H), 6.85 (d, J = 8.3 Hz, 1H), 6.75 (d, J = 2.4 Hz, 1H), 6.68 (dd, J = 8.4, 2.4 Hz, 1H), 6.03 (s, 2H). 1^3^C NMR (100 MHz, CDCl_3_) *δ* 168.3, 157.4, 154.1, 148.5, 146.1, 146.0, 145.9, 130., 127.7, 120.9, 115.8, 114.0, 108.4, 103.7, 102.1. HRMS (ESI): calculated for C_15_H_9_N_3_O_5_ [M + H]^+^:312.061497, found: 312.06163.

#### 4-(benzo[d][1,3]dioxol-5-yloxy)-7-chloroquinazoline(4t)

White solid; m.p. 141.5 ~ 142.7 °C; yield, 71.34%. ^1^H NMR (400 MHz, CDCl_3_) *δ* 8.78 (s, 1H), 8.30 (d, J = 8.8 Hz, 1H), 8.01 (d, J = 2.0 Hz, 1H), 7.63 (dd, J = 8.8, 2.1 Hz, 1H), 6.88 (d, J = 8.4 Hz, 1H), 6.76 (d, J = 2.4 Hz, 1H), 6.70 (dd, J = 8.3, 2.4 Hz, 1H), 6.04 (s, 2H). ^13^C NMR (100 MHz, CDCl_3_) *δ* 160.4, 151.1, 150.2, 148.8, 147.8, 141.1, 138.9, 128.5, 127.9, 126.7, 121.0, 113.2, 110.9, 101.8, 98.0.

### Fungal strains and culture conditions

*Pestalotiopsis* sp. and *Colletotrichum camelliae* were provided by the Guizhou Tea Research Institute (Guiyang, China). *Phomopsis* sp., *Sclerotinia sclerotiorum*, *Phytophthora capsici*, and *Botrytis cinerea Pers. Fr.* were isolated and identified in our laboratory (voucher specimens are deposited at Guizhou University). All strains were cultured on potato dextrose agar (PDA) at 25 ± 1 °C and stored at 4 °C for short-term preservation.

### In vitro antifungal activity assay

Mycelial growth inhibition assays were performed as described previously with modifications [23, 24]. Test compounds were dissolved in DMSO (1%, v/v) and diluted with sterile water to 100 μg/mL. Aliquots (1 mL) of the compound solution were added to molten PDA (50 °C, 9 mL) to prepare drug-containing medium. After solidification, a 5 mm-diameter mycelial plug (from 7-day-old PDA cultures) was inoculated onto the center of each plate. Plates containing 1% DMSO (without compounds) served as blank controls, and azoxystrobin (100 μg/mL) was used as a positive control. Each treatment was replicated three times. Plates were incubated at 28 ± 1 °C, and colony diameter was measured when the control colony reached 80% of the plate area. The inhibition rate was calculated as: ${\text{Inhibition rate }}\left( \% \right)\, = \,\left[ {\left( {{\text{Colony diameter of control}}{-}{\text{Colony diameter of treatment}}} \right)/\left( {{\text{Colony diameter of control}}{-}{\text{5 mm}}} \right)} \right]\, \times \,{1}00$.

### In vivo antifungal activity assay

One-year-old *C. sinensis cv*. Longjing 43 plants were grown in a greenhouse (25 ± 2 °C, 16 h light/8 h dark). For *Pestalotiopsis* sp. inoculation: leaves were wounded with sterile forceps, and 5 mm mycelial plugs were placed on the wounds. After 24 h, test compounds (100 μg/mL, dissolved in 1% DMSO) were sprayed onto leaves until runoff. azoxystrobin (100 μg/mL) and 1% DMSO were used as positive and blank controls, respectively. Lesion length was measured, and each treatment included 3 plants (10 leaves per plant), and the experiment was repeated thrice[25].

### Morphological observation of fungal mycelia

Fluorescence microscopy (FM): *Pestalotiopsis* sp. mycelia were cultured in potato dextrose broth (PDB) with test compound (100 μg/mL) for 24 h. Mycelia were stained with propidium iodide (PI, 20 μg/mL) for 15 min at 37 °C in the dark, washed with PBS (0.1 M, pH 7.4), and observed under a fluorescence microscope [26].

Scanning electron microscopy (SEM): Mycelia treated with test compound (100 μg/mL) for 24 h were fixed in 2.5% glutaraldehyde (4 °C, 24 h), dehydrated in a graded ethanol series (30–100%, v/v), and freeze-dried. Samples were sputter-coated with gold and observed under the SEM [27].

### Defense enzyme activity assays

Tea leaves were inoculated with *Pestalotiopsis* sp. mycelial plugs as described in Sect. 2.5. After 24 h, leaves were sprayed with test compound (100 μg/mL), azoxystrobin (100 μg/mL), or 1% DMSO (control). Leaf samples were collected at 1, 3, and 5 days post-treatment, ground into powder in liquid nitrogen, and homogenized in extraction buffer (0.1 M PBS, pH 7.0, containing 1% PVP). The supernatant was used to determine superoxide dismutase (SOD), peroxidase (POD), catalase (CAT), and phenylalanine ammonia-lyase (PAL) activities using commercial kits (Shanghai Yuanxin Biotechnology Co., Ltd.) according to the manufacturer’s instructions. Enzyme activity was measured with a microplate reader (BioTek ELx808, Winooski, VT, USA), with three biological replicates per treatment.

### Transcriptomic and proteomic analysis

The target compound was initially dissolved in DMSO, and a 100 μg/mL test solution was prepared using 0.1% Tween-80 solution. The same treatment without target compound served as the CK *Pestalotiopsis sp* pathogens were inoculated into wounds, and 24 h later, the prepared test solutions were uniformly sprayed onto tea plant leaves. Leaf samples collected on days 1, 3, and 5 after treatment (including the compound-treated group and the water control group). Then the samples were entrusted to Wuhan Igene Book Biotechnology Co., Ltd. for total RNA extraction, quality detection, cDNA library construction, and RNA-Seq sequencing analysis.

The treatment method for tea plant leaf samples was consistent with the transcriptome analysis workflow. After grinding the leaves into powder, proteins were extracted and identified from leaf samples of the treatment group (compound + *Pestalotiopsis sp*) and control group (CK + *Pestalotiopsis sp*) at days 1, 3 and 5, post-spraying with target compound, following the methodology described in previous experiments. Functional analysis of differentially expressed proteins (DEPs) was performed using the Gene Ontology (GO) database, with screening criteria set as p < 0.05 and fold change > 1.5. Eligible DEPs were classified into biological processes (BPs), cellular components (CCs), and molecular functions (MFs), and the number of proteins corresponding to each GO term was statistically analyzed. Meanwhile, Kyoto Encyclopedia of Genes and Genomes (KEGG) database was used for signaling pathway annotation of DEPs to identify major metabolic and signaling pathways enriched with differentially expressed proteins.

## Supplementary Information

**Additional file 1.**

## Data Availability

The experimental data supporting this work are accessible within the article and its additional file.
