# Supplementary material for: Sesamol derivatives bearing a quinazolin moiety: synthesis, antifungal efficacy against tea plant pathogens, and mechanistic insights
Source: Nat Prod Bioprospect. 2026 Jun 1;16(1):66. doi: 10.1007/s13659-025-00568-x (PMC13226774; doi:10.1007/s13659-025-00568-x)
Supplement: Supplementary file 1 — Additional file 1. [file 13659_2025_568_MOESM1_ESM.docx]

**Supplementary Data**

**Sesamol Derivatives Bearing a Quinazolin Moiety: Synthesis, Antifungal Efficacy Against Tea Plant Pathogens, and Mechanistic Insights**

Haixia Tang^†1,^ Shiyi Liu^†2^, Fali Wang^3^, Chao Gao^2^, Linhong Jin*^1,3^, Dandan Xie*^1,3^

^1^ *State Key Laboratory of Green Pesticide, Guizhou University, Guiyang 550025, China*

^2^ *College of Forestry, Guizhou University,* *Huaxi District, Guiyang 550025, China*

^3^ *College of Tea Science, Guizhou University, Huaxi District, Guiyang 550025, China*

^†^ These authors contributed equally to this work

* Author to whom correspondence should be addressed; E-mail: xddxed@163.com


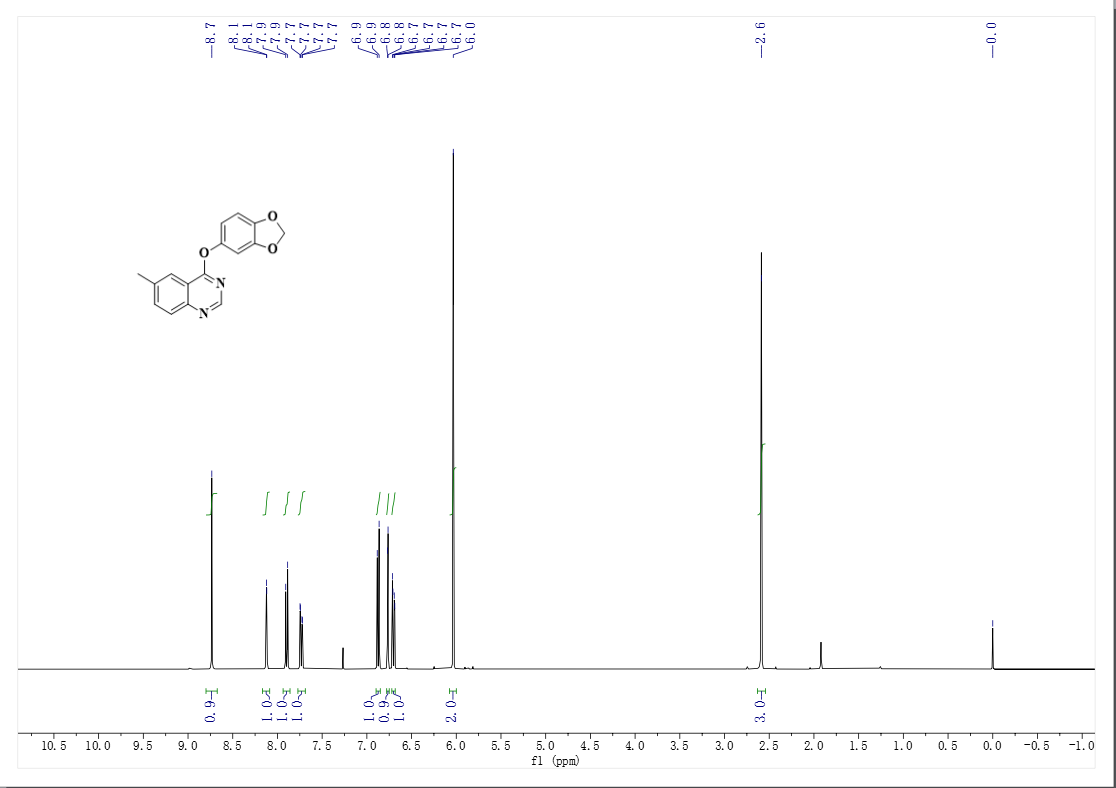


^1^H NMR of Compound 4a


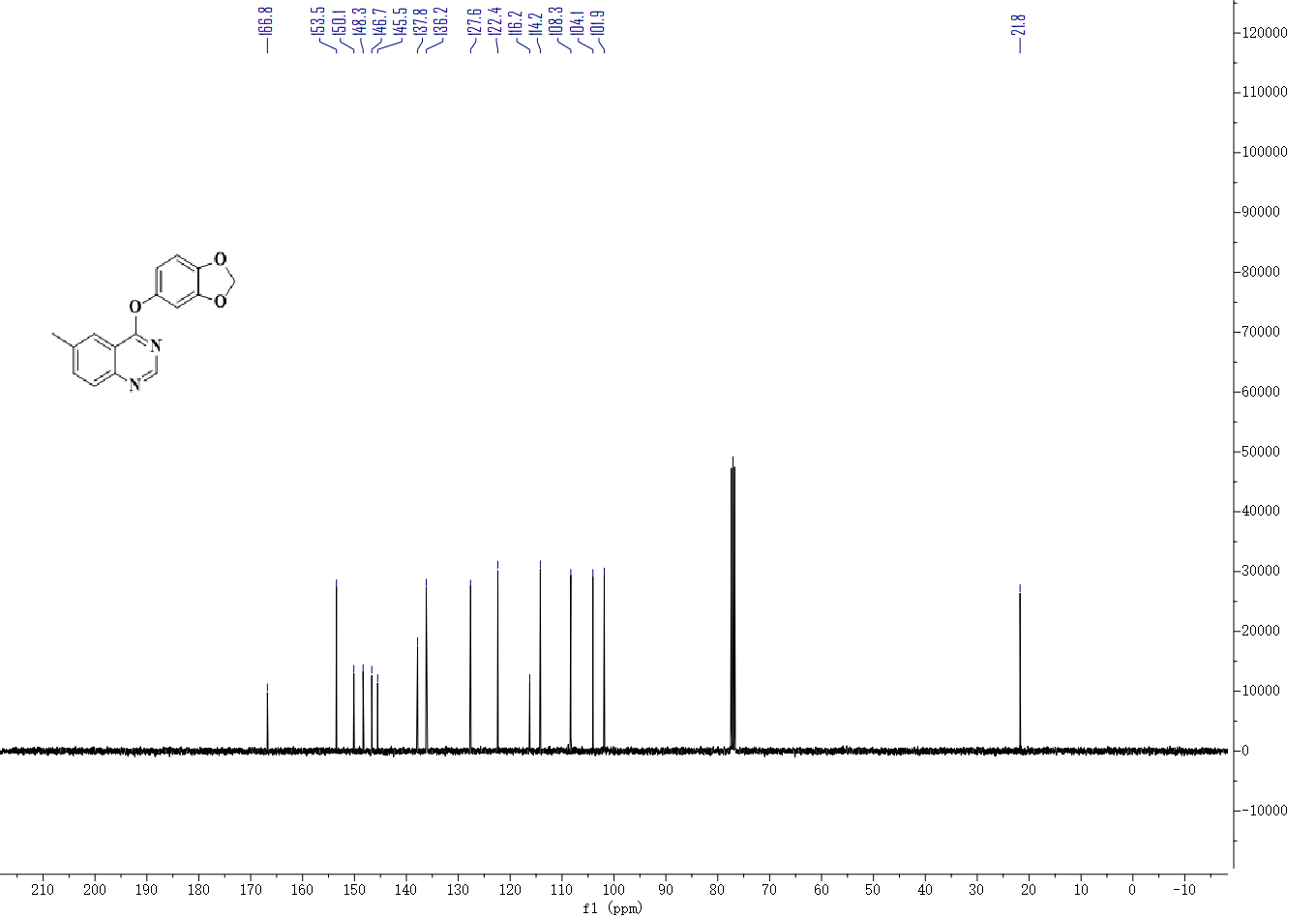


^13^C NMR of Compound 4a


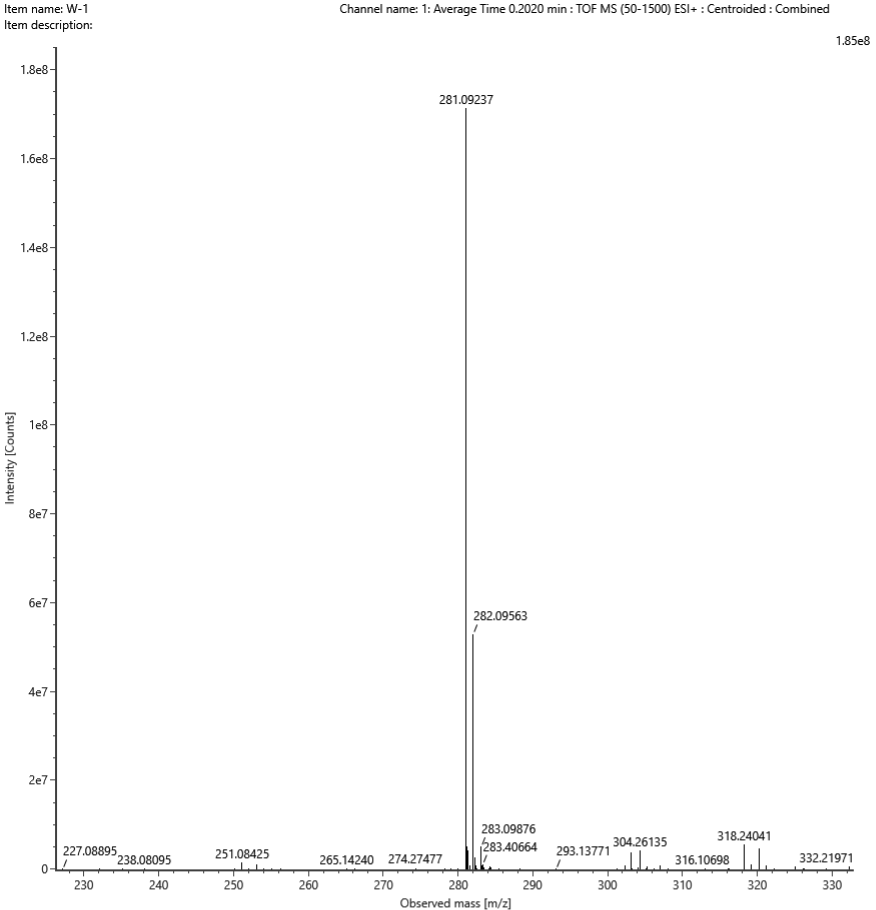


HRMS of Compound 4a


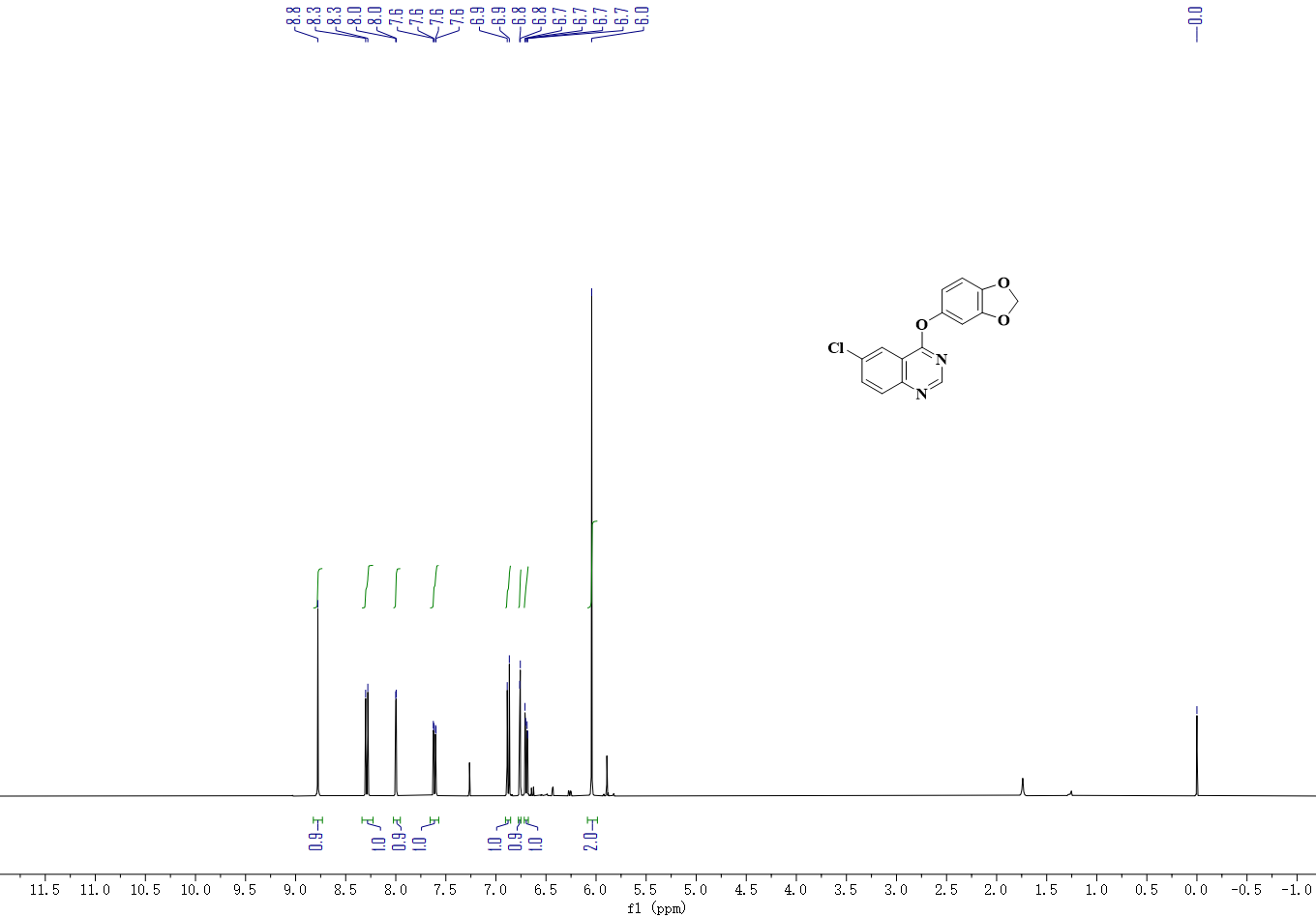


^1^H NMR of Compound 4b


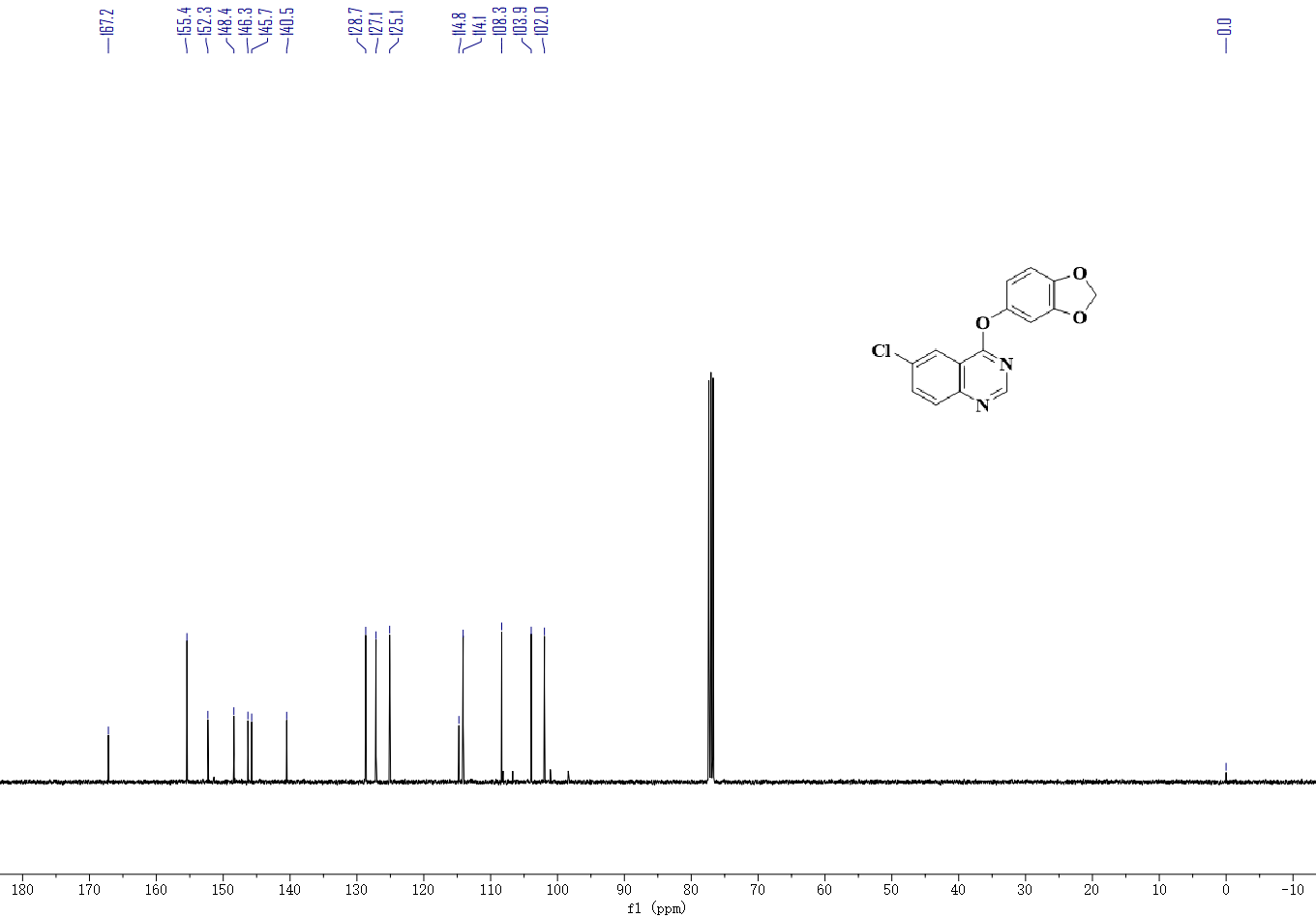


^13^C NMR of Compound 4b


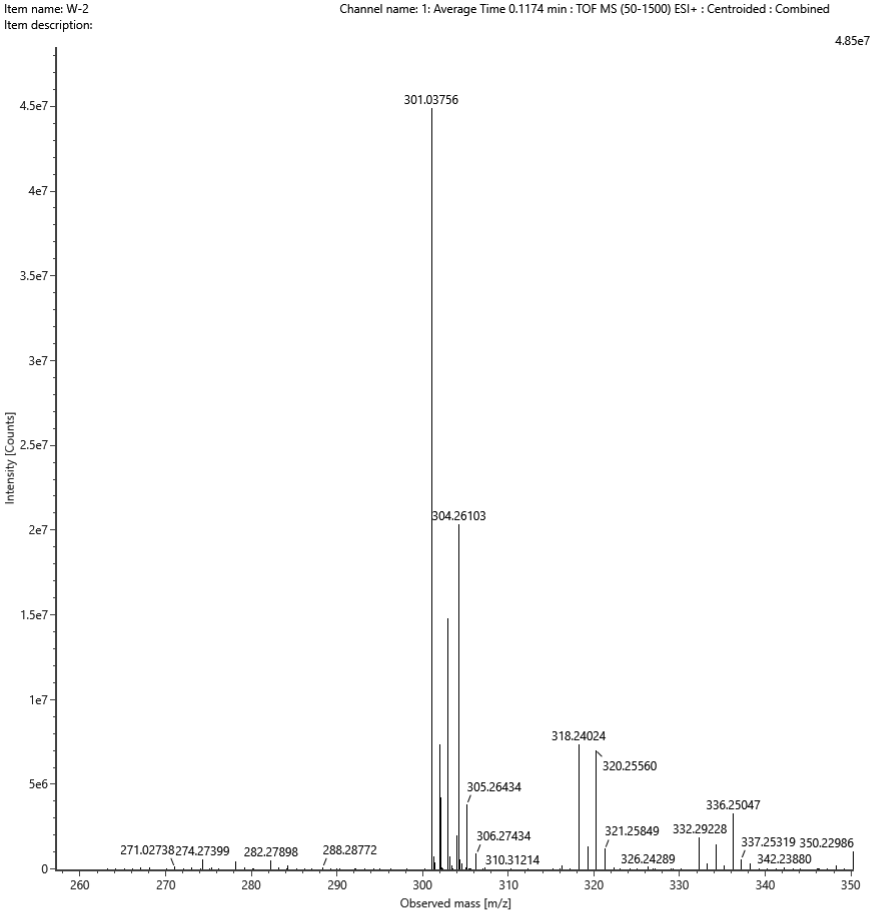


HRMS of Compound 4b


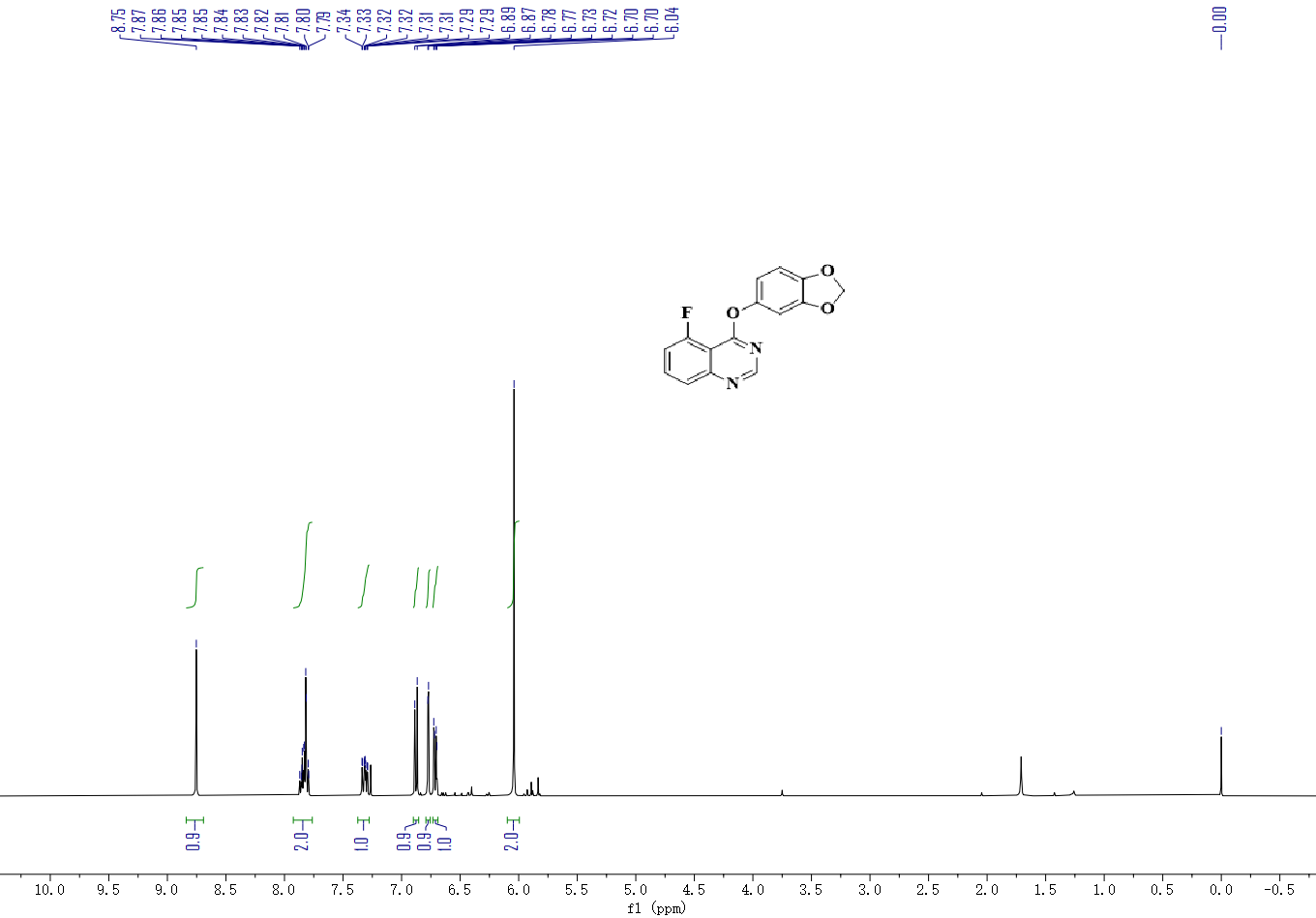


^1^H NMR of Compound 4c


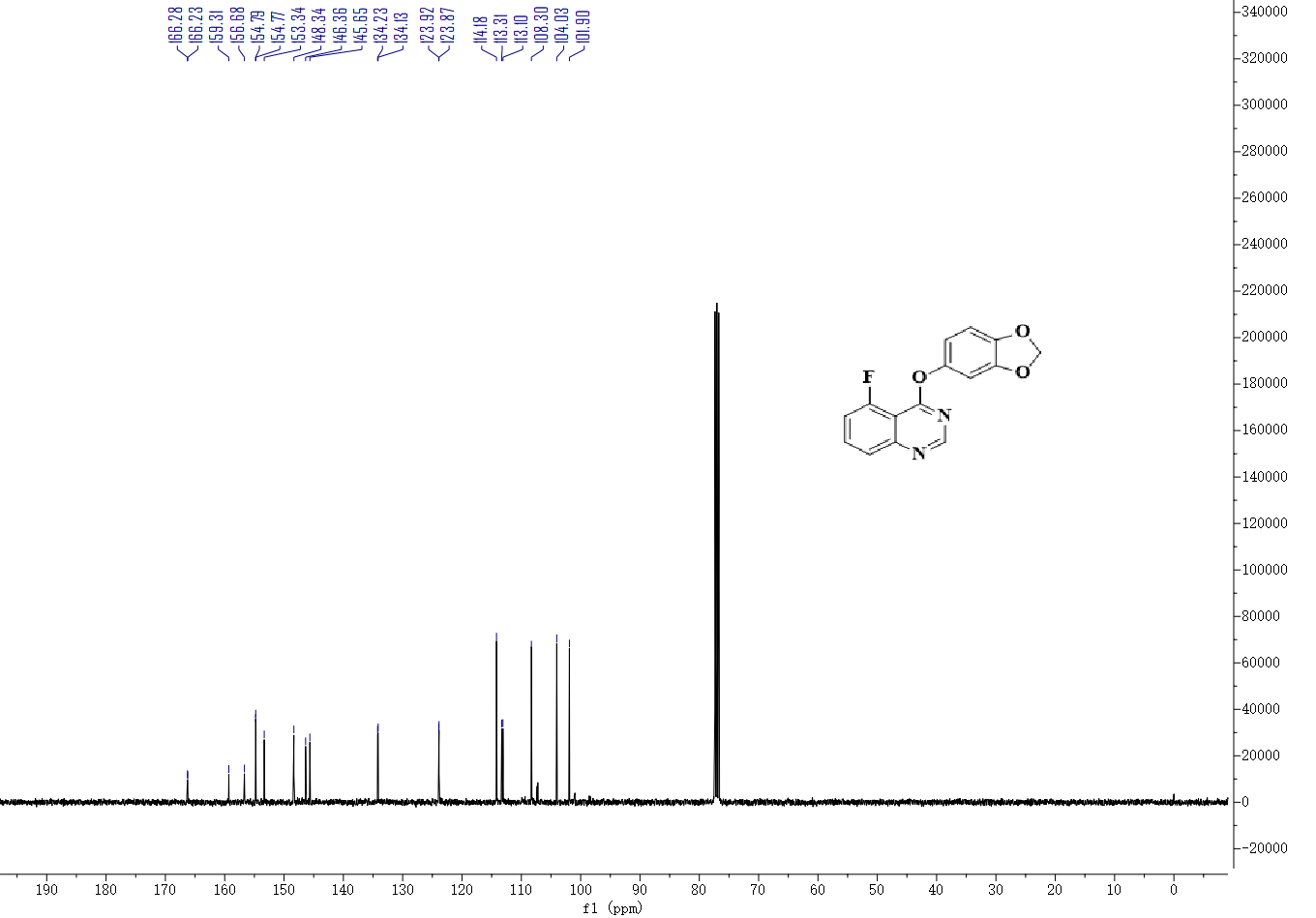


^13^C NMR of Compound 4c


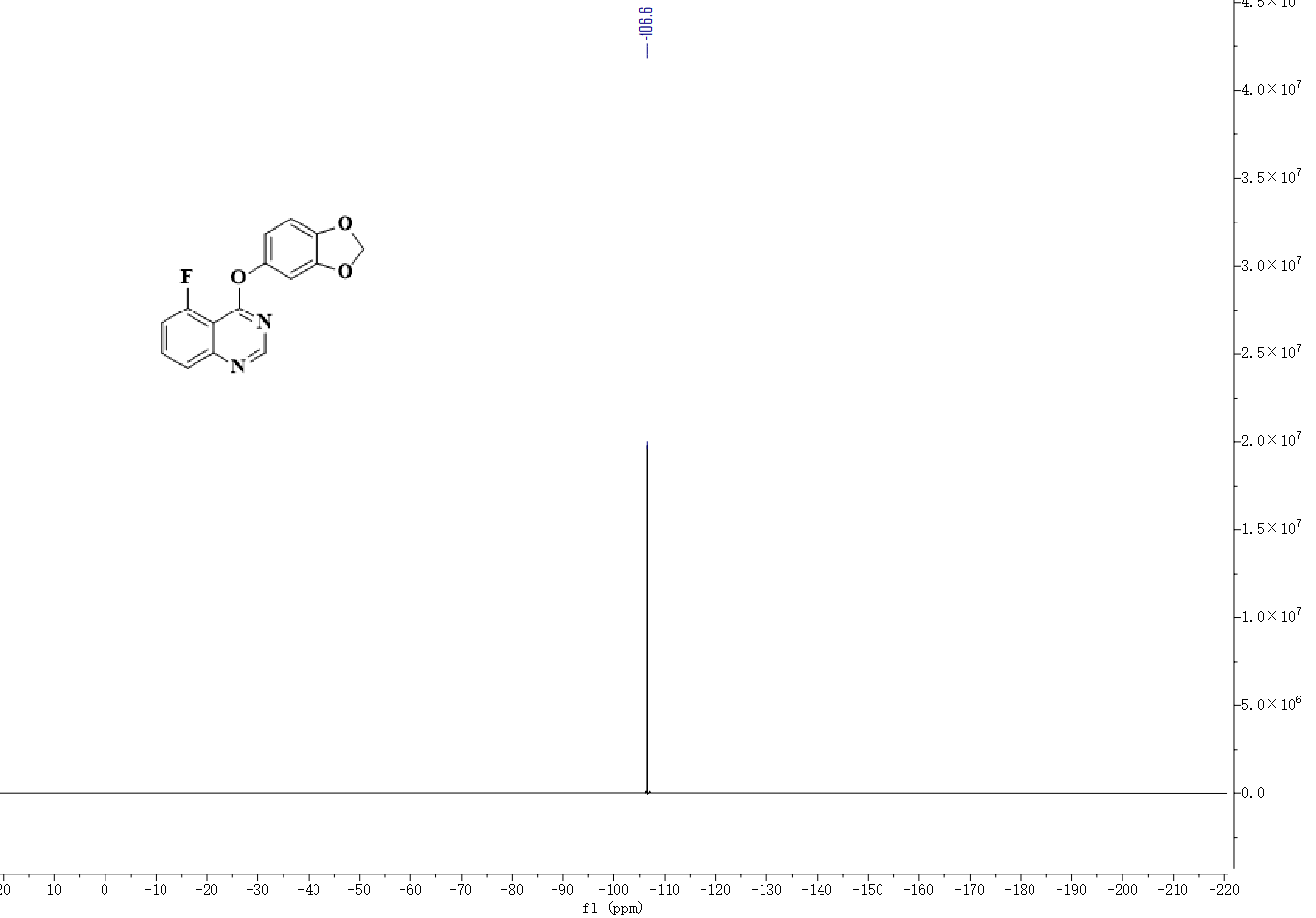


^19^F NMR of Compound 4c


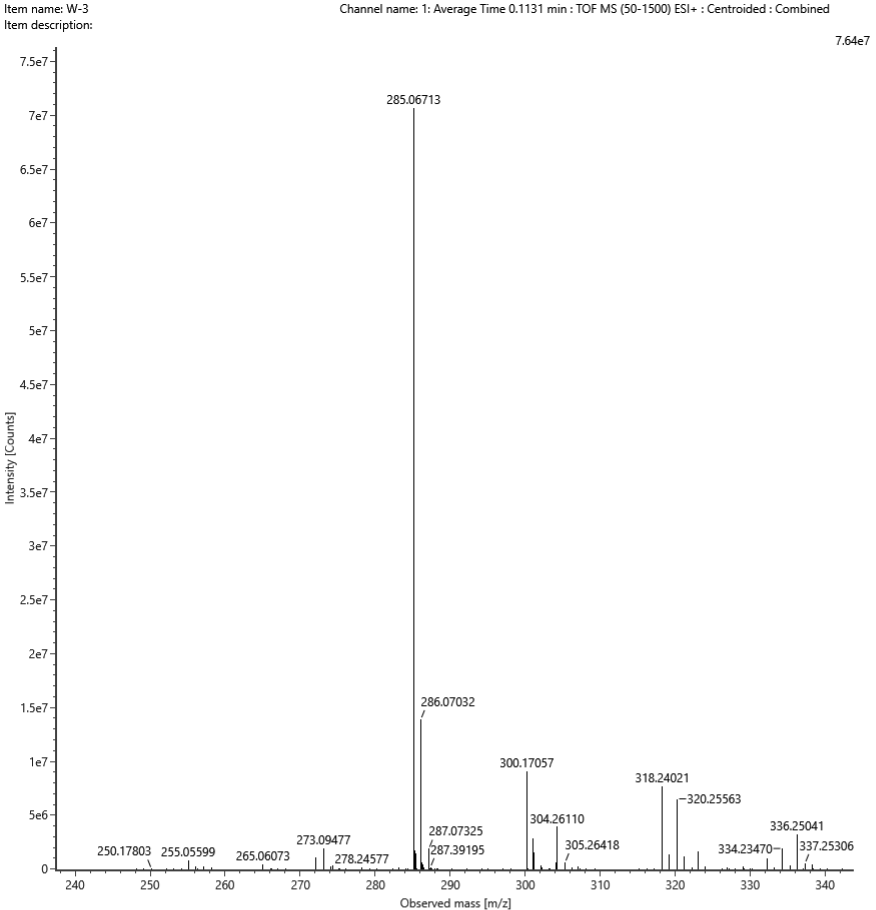


HRMS of Compound 4c


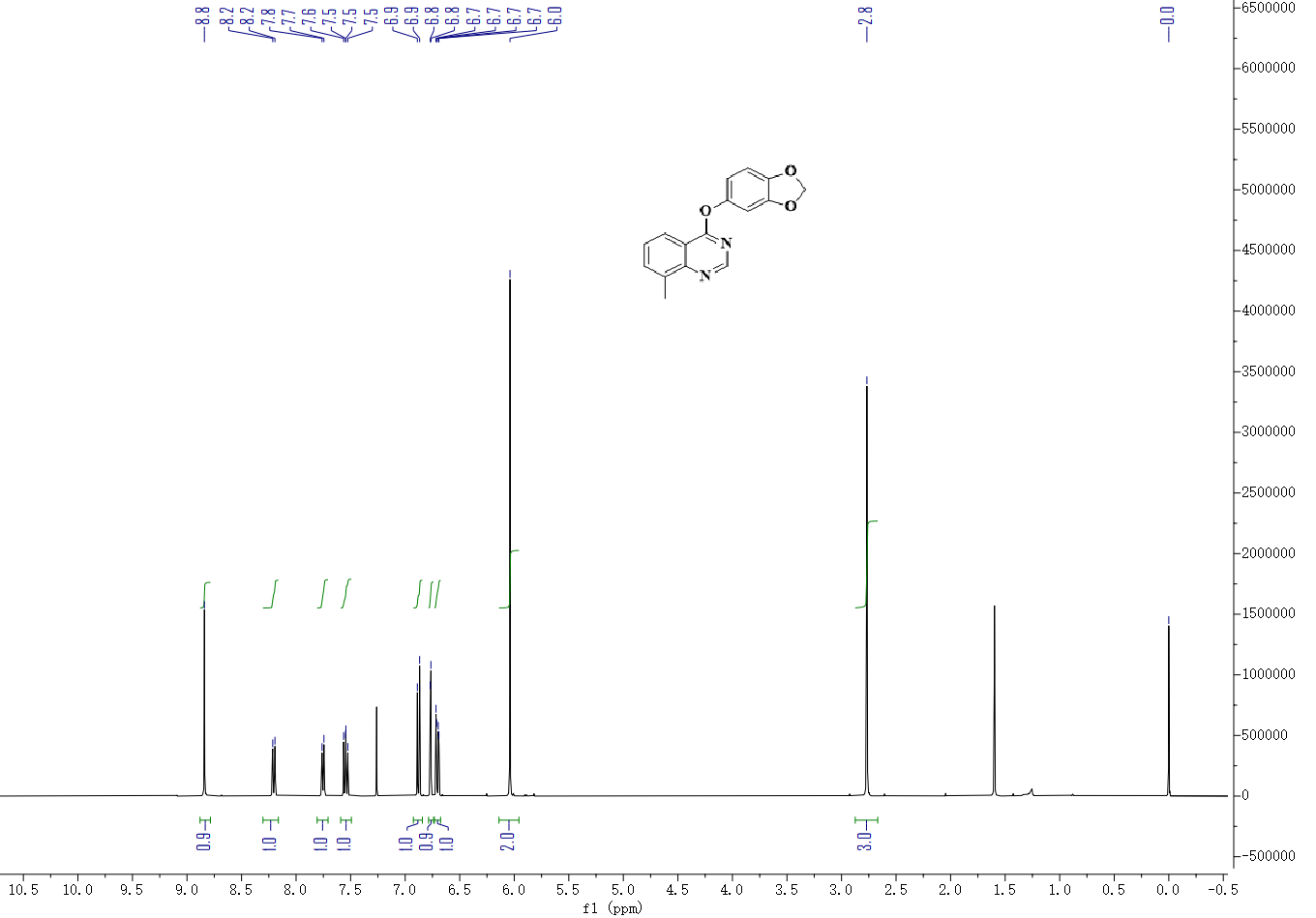


^1^H NMR of Compound 4d


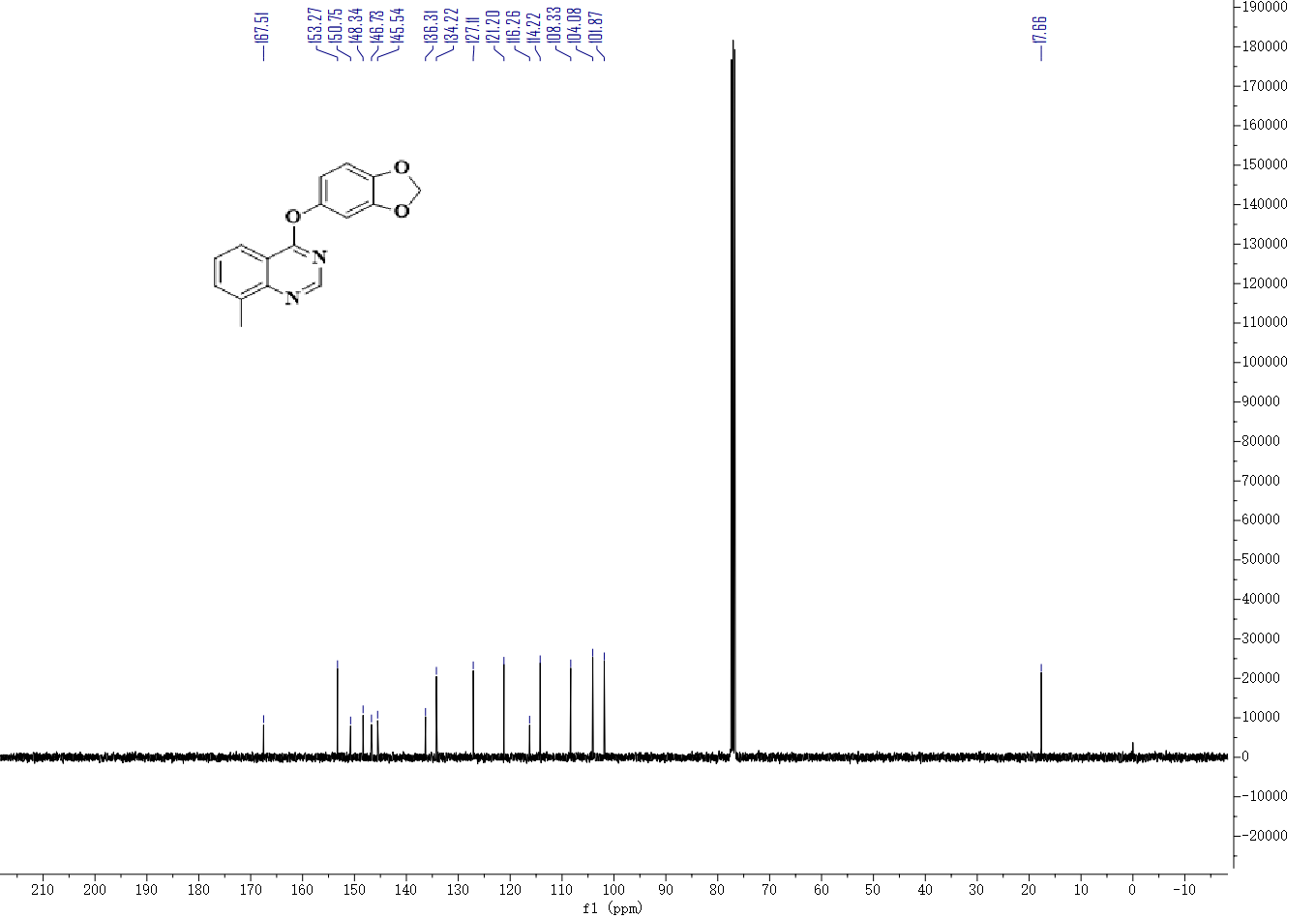


^13^C NMR of Compound 4d


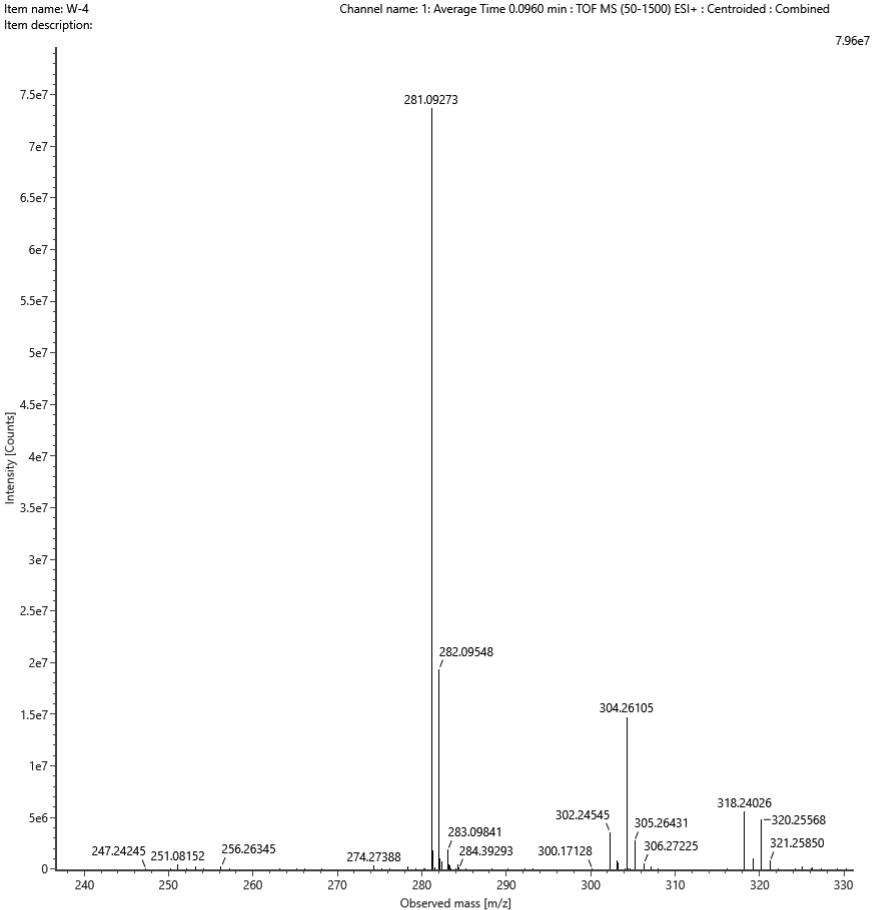


HRMS of Compound 4d


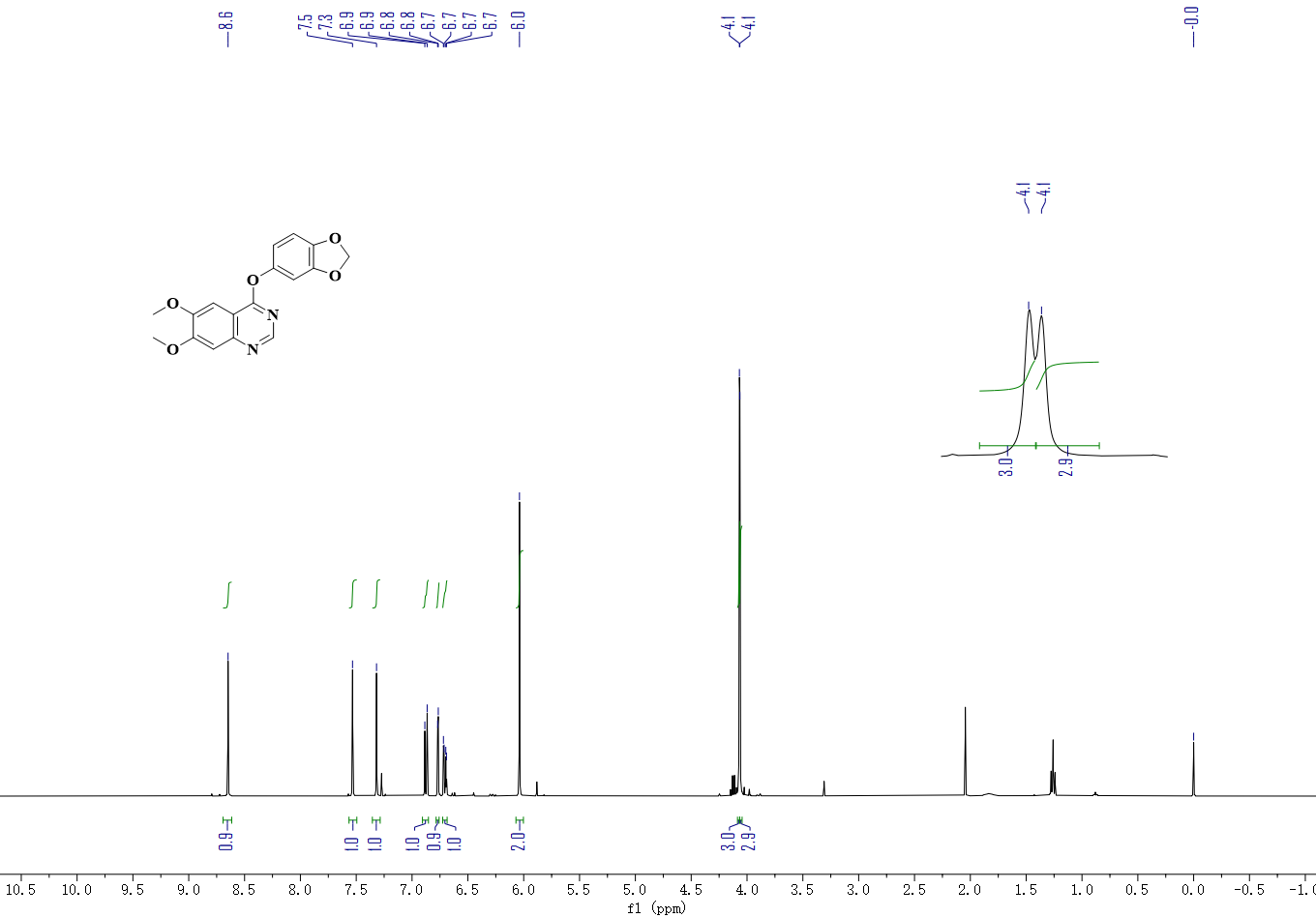


^1^H NMR of Compound 4e


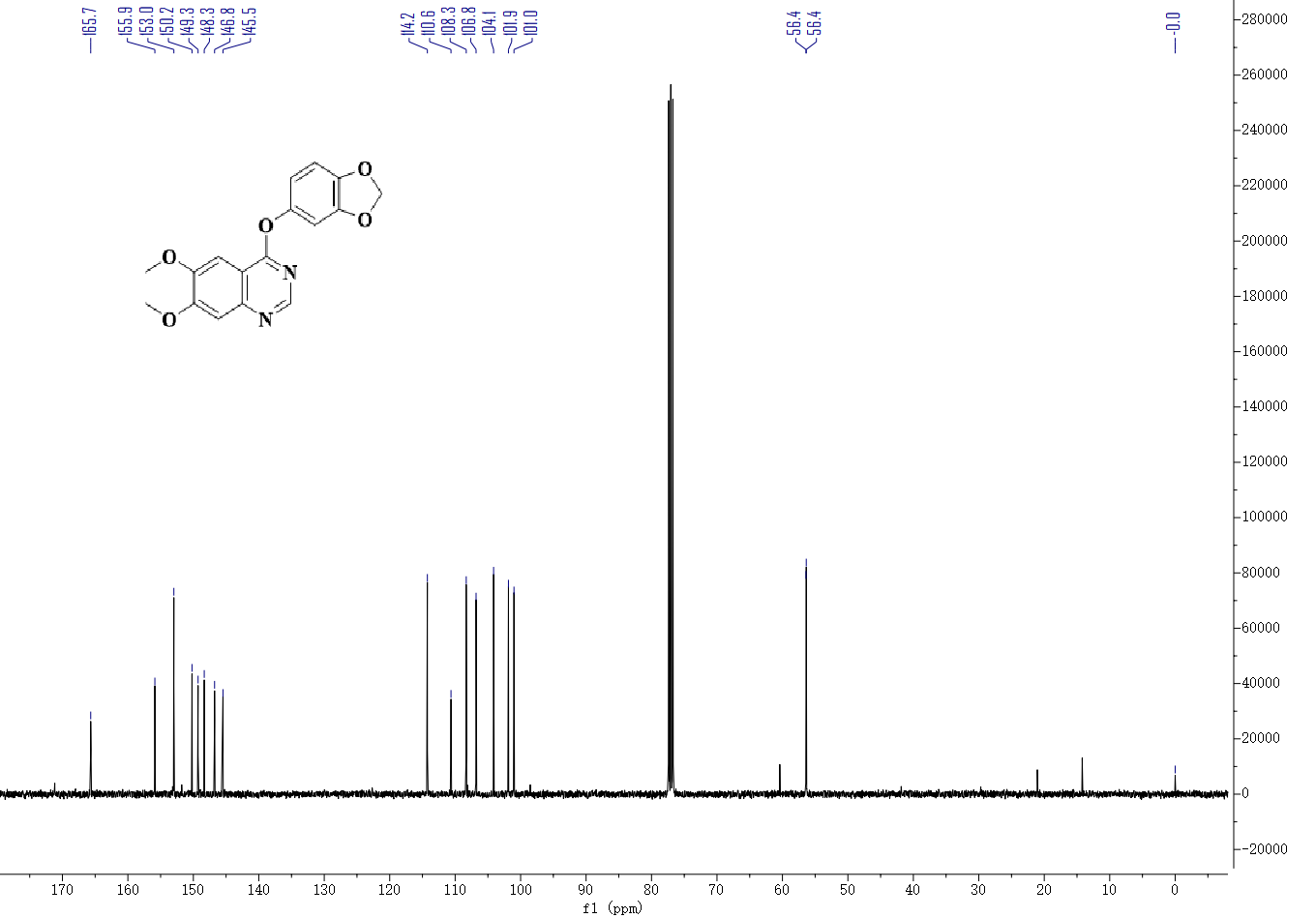


^13^C NMR of Compound 4e


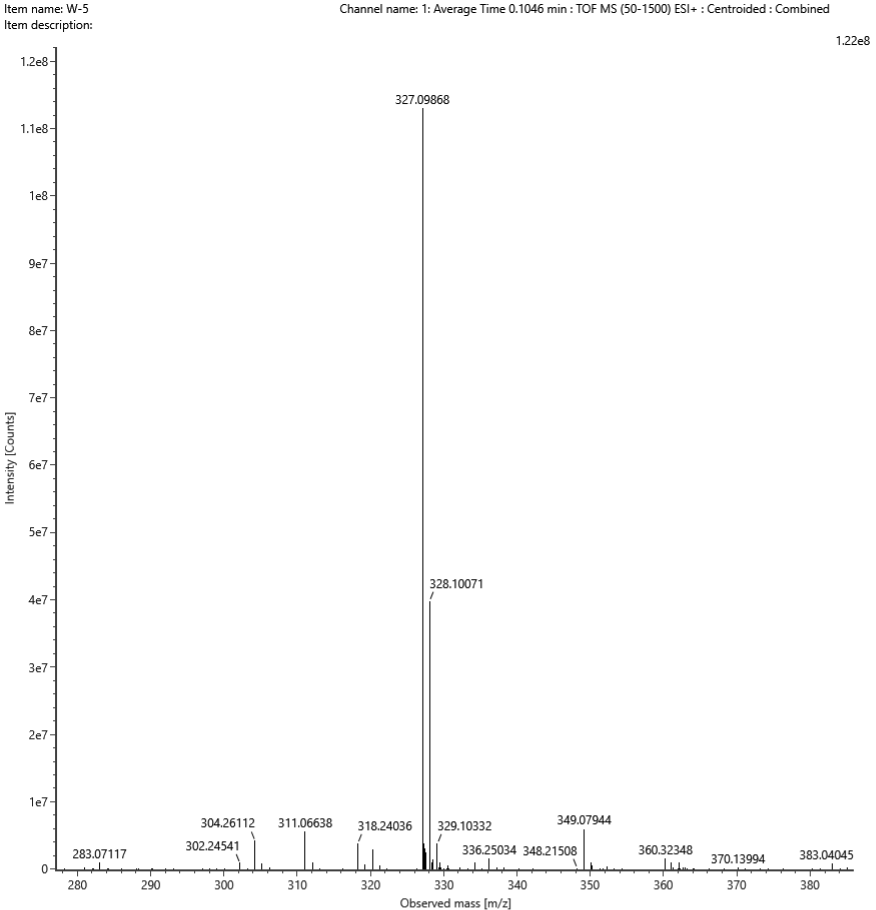


HRMS of Compound 4e


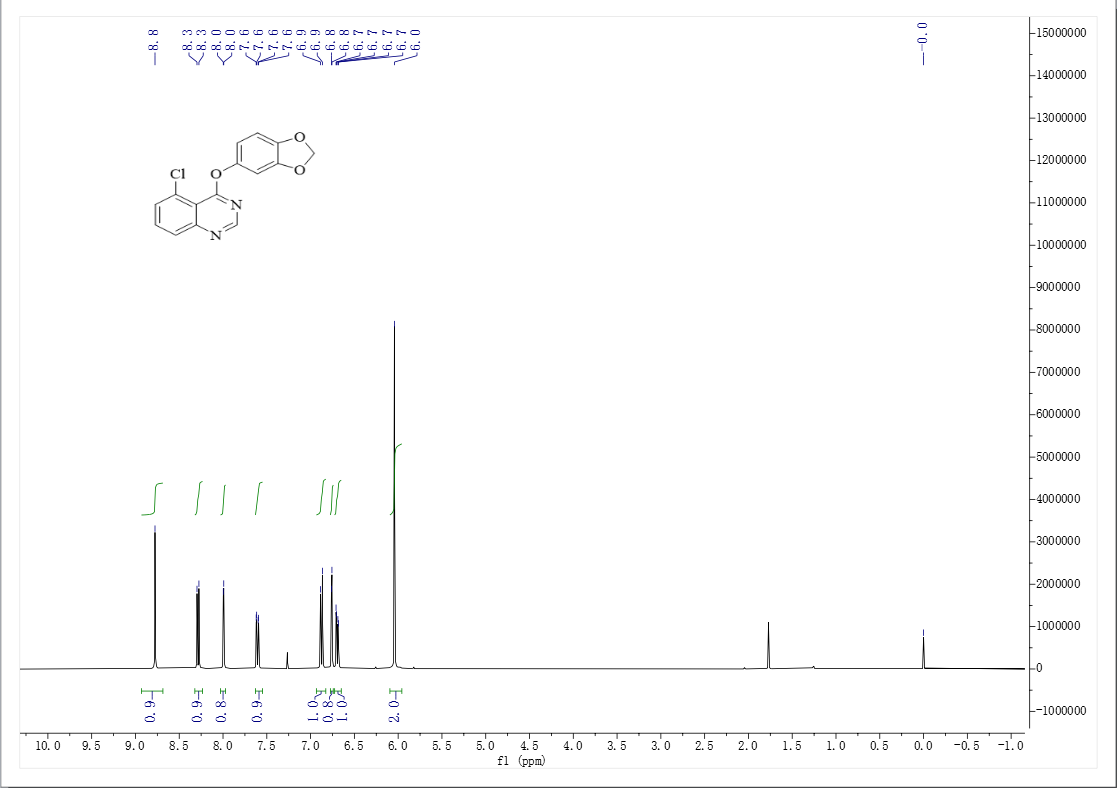


^1^H NMR of Compound 4f


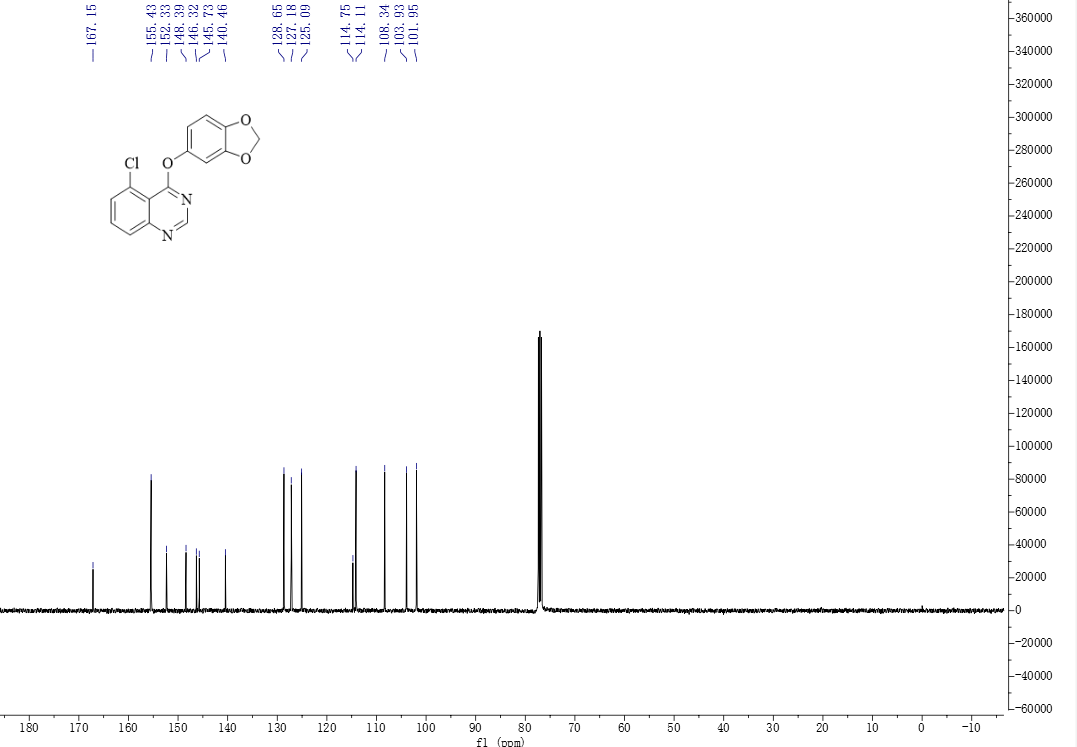


^13^C NMR of Compound 4f


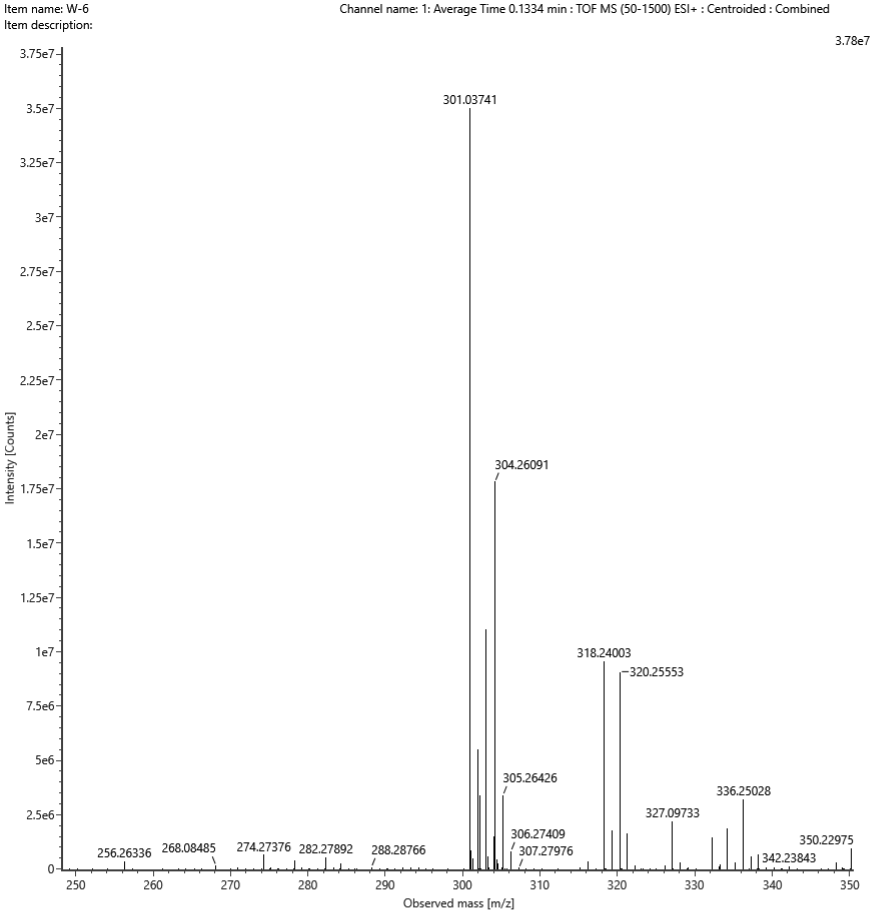


HRMS of Compound 4f


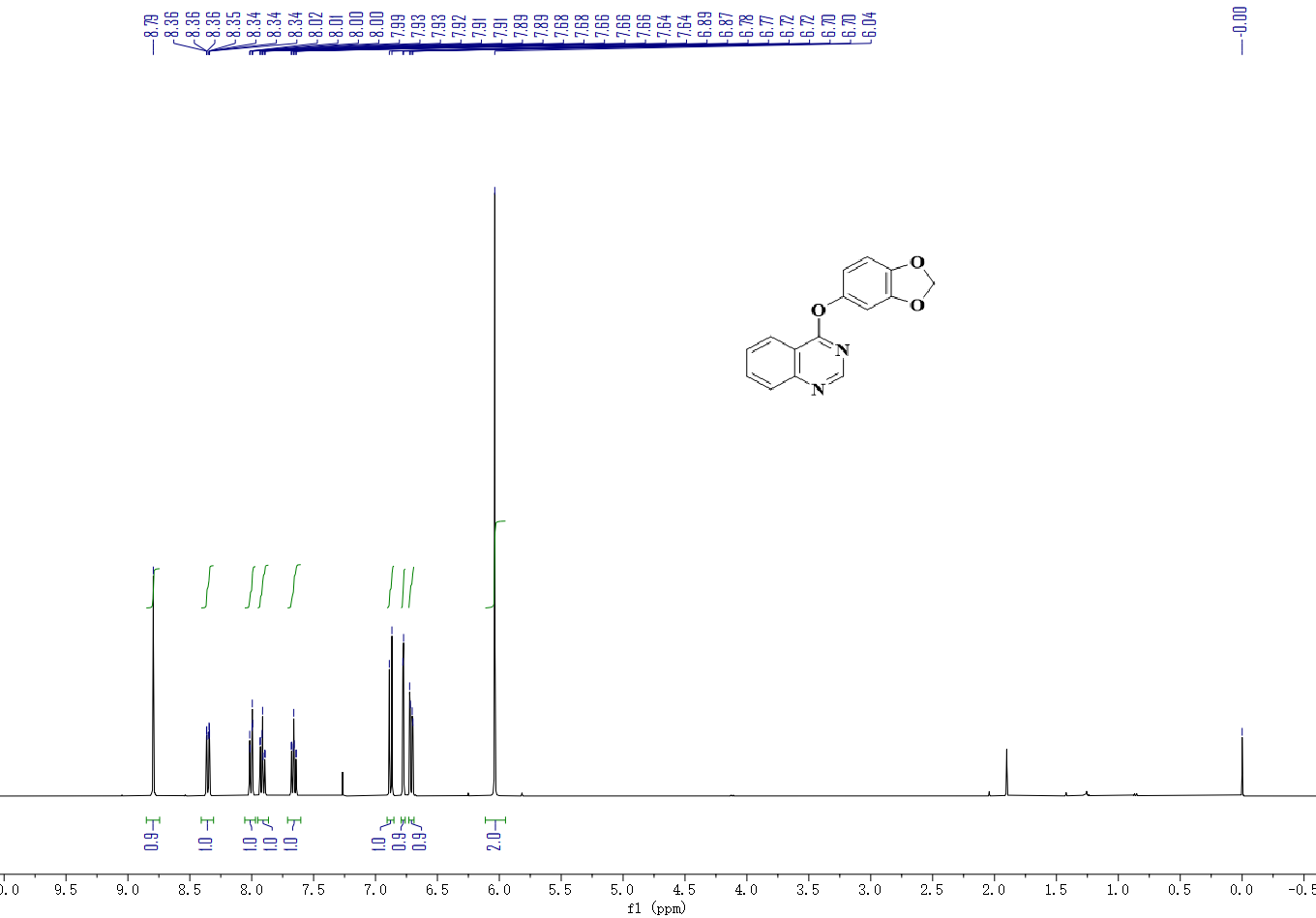


^1^H NMR of Compound 4g


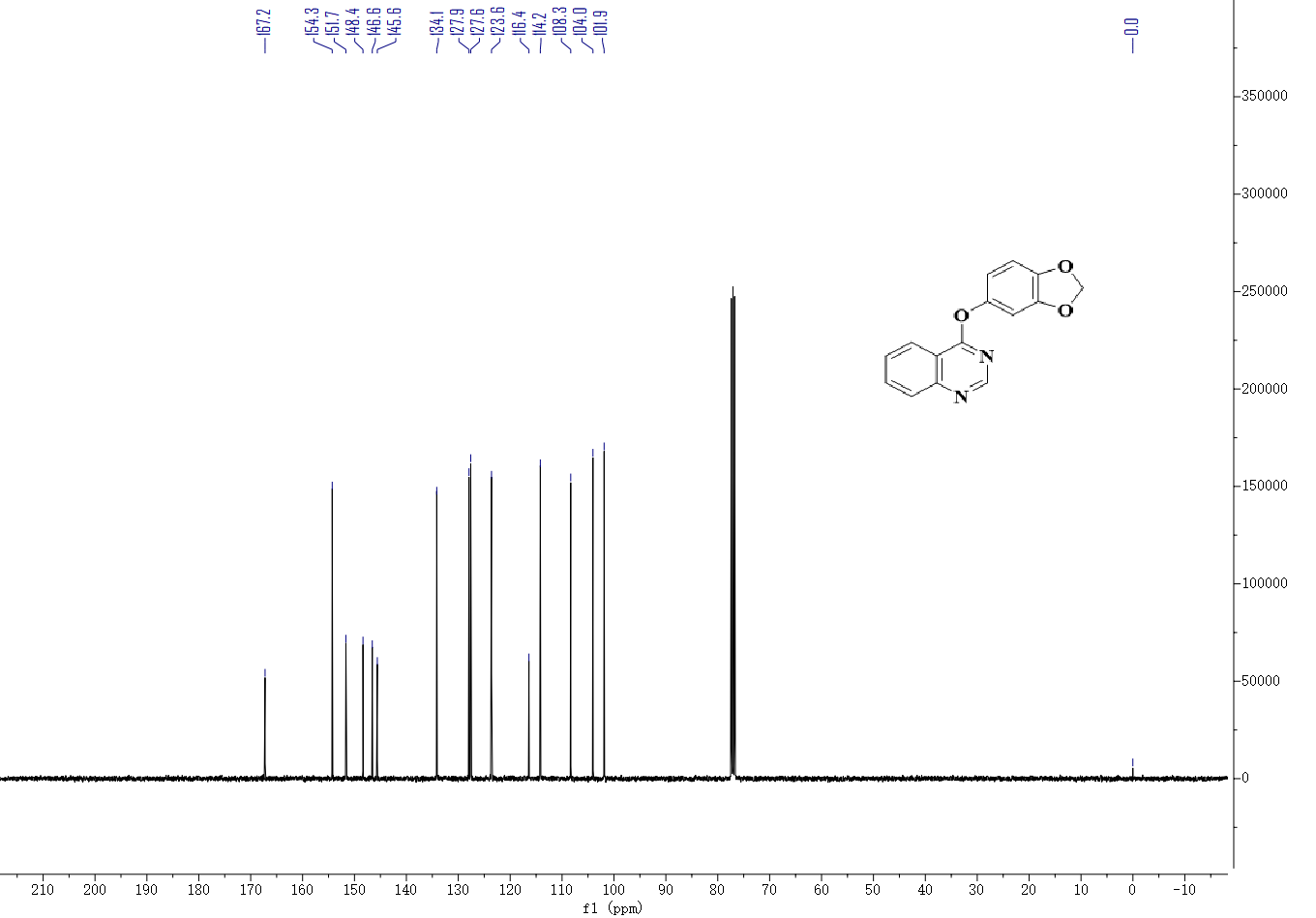


^13^C NMR of Compound 4g


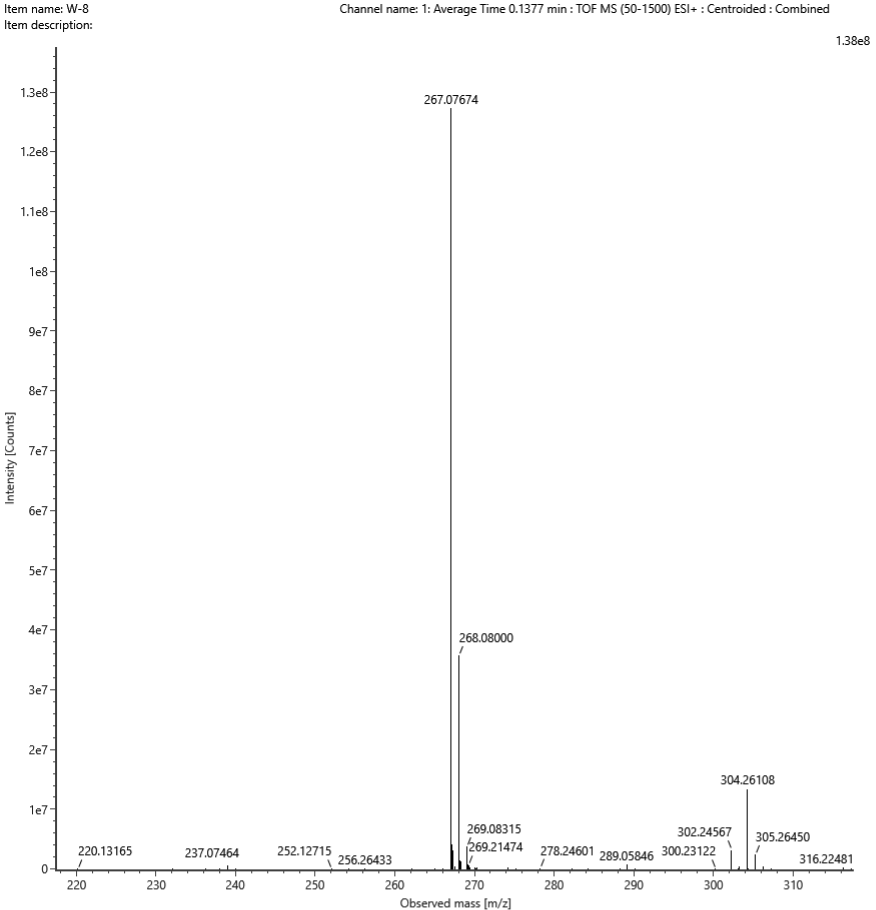


HRMS of Compound 4g


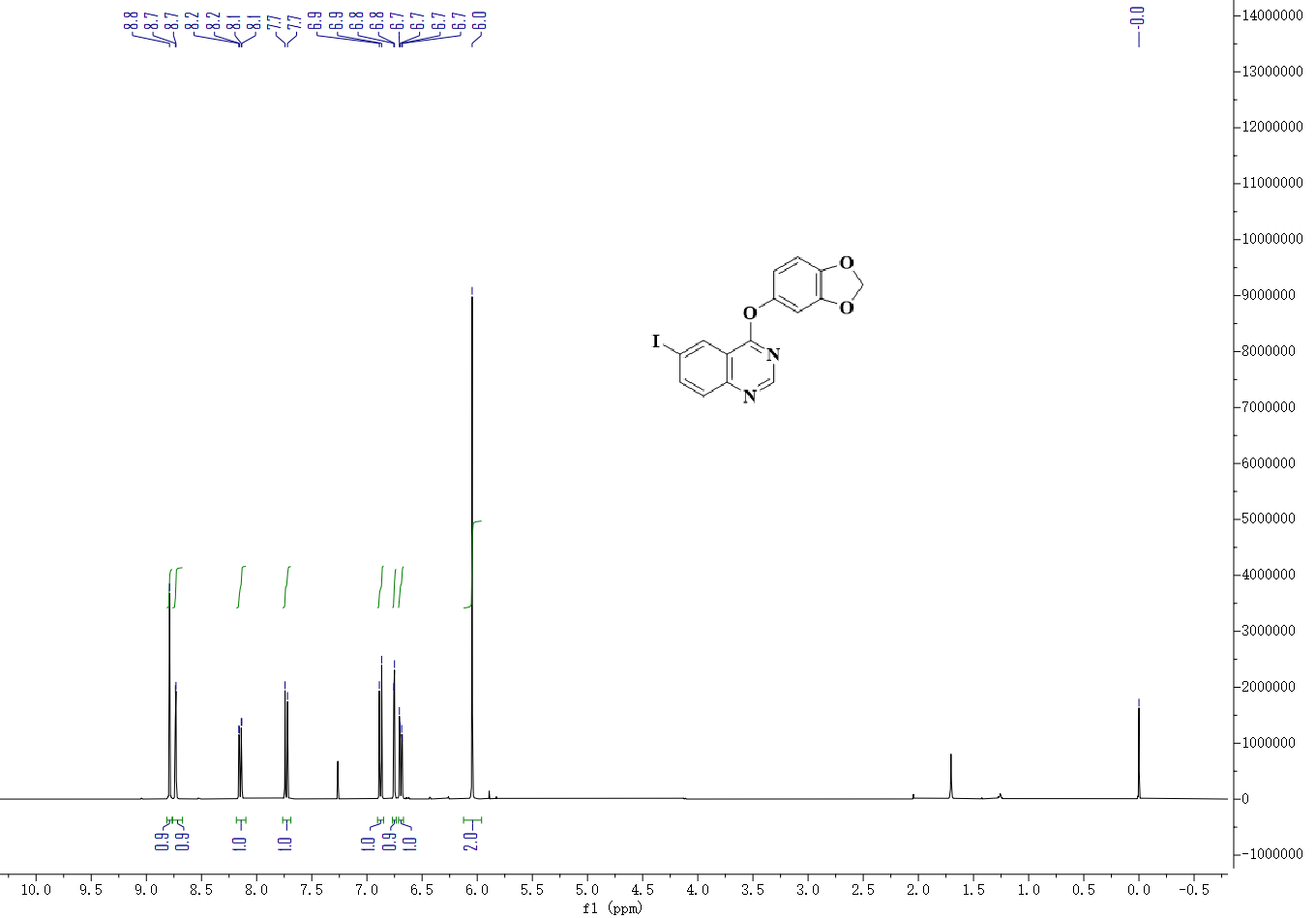


^1^H NMR of Compound 4h


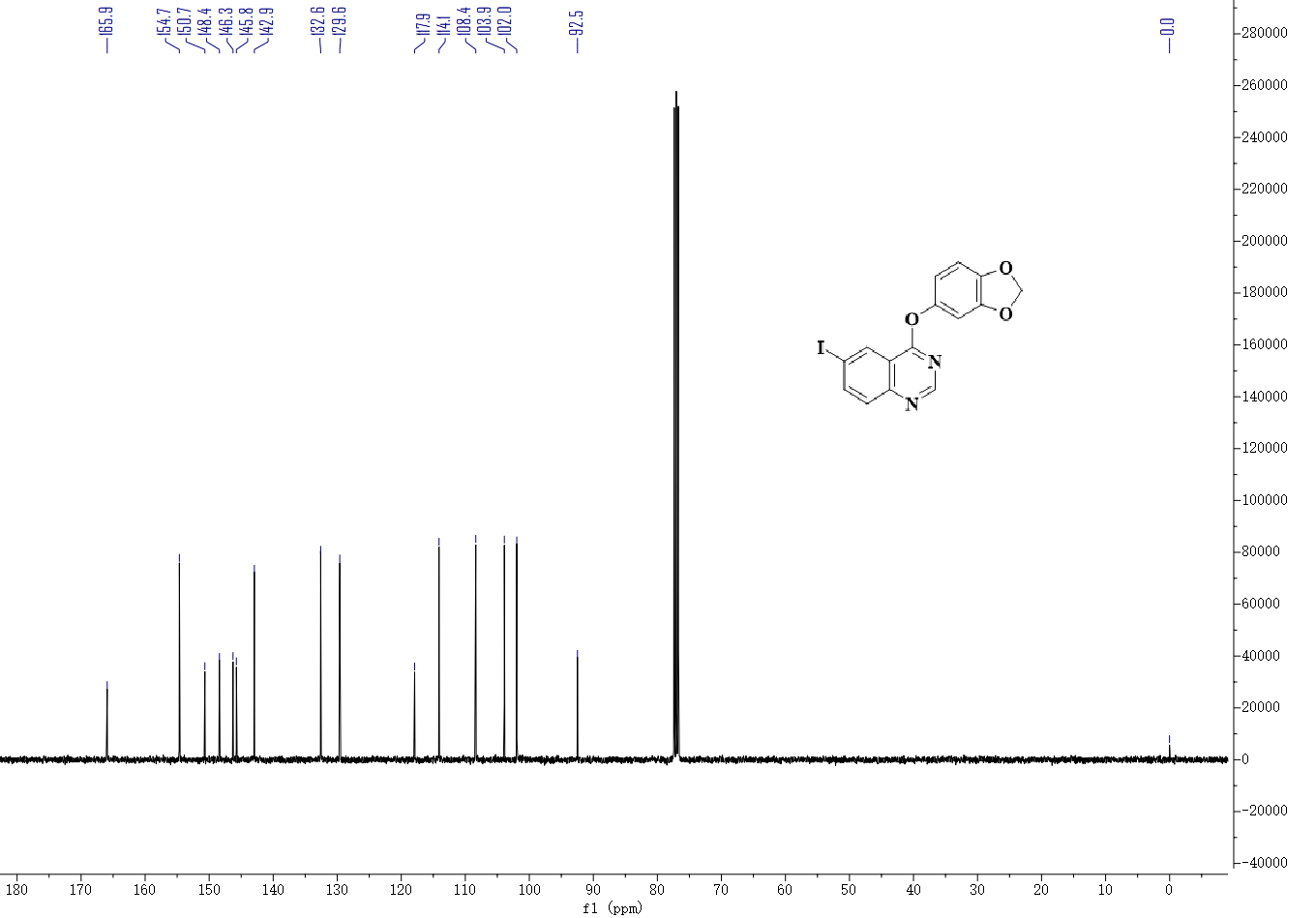


^13^C NMR of Compound 4h


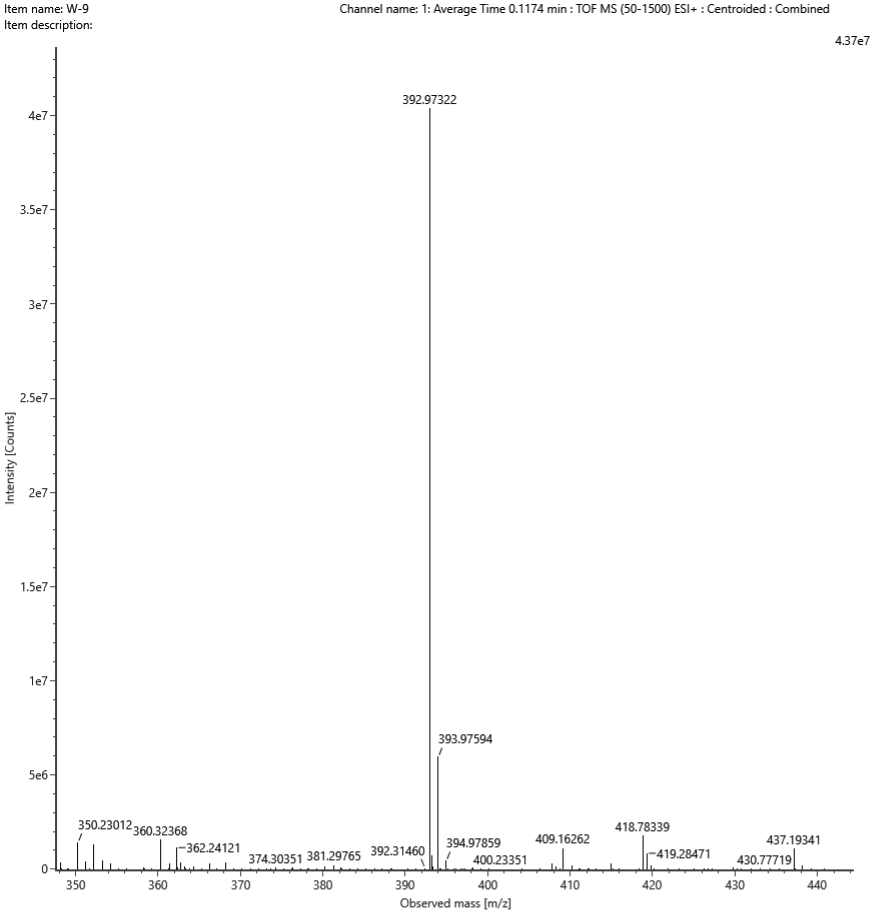


HRMS of Compound 4h


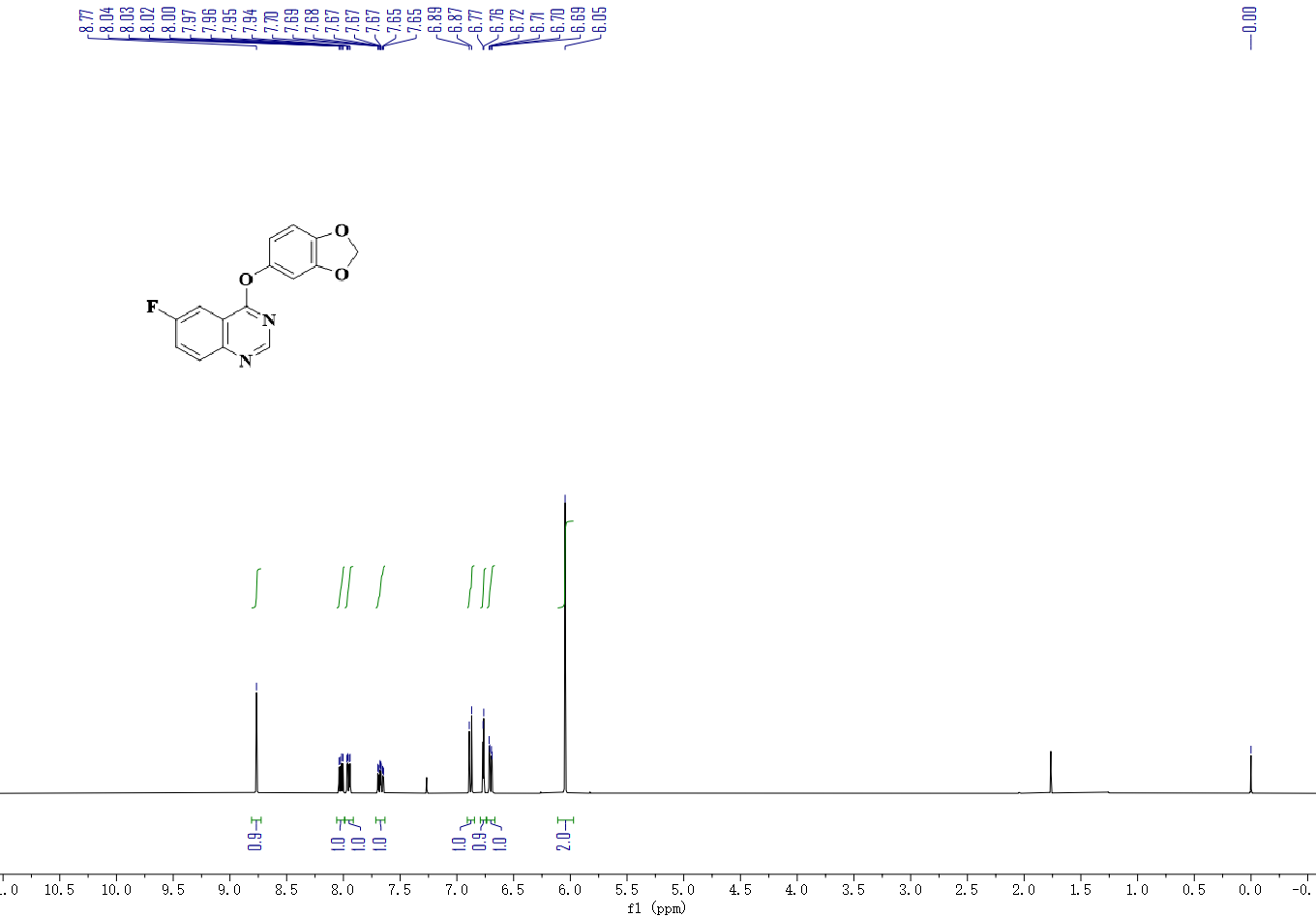


^1^H NMR of Compound 4i


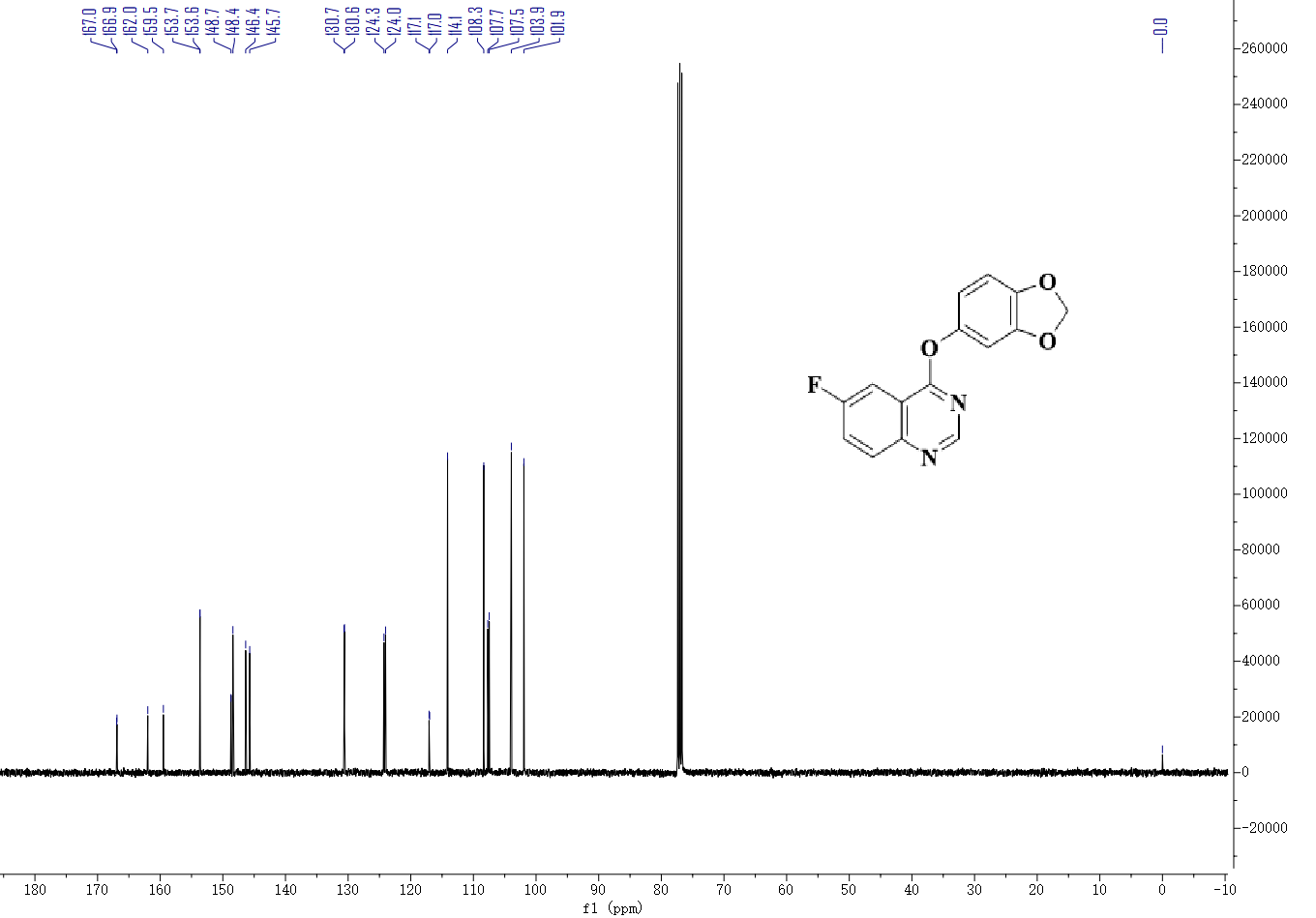


^13^C NMR of Compound 4i


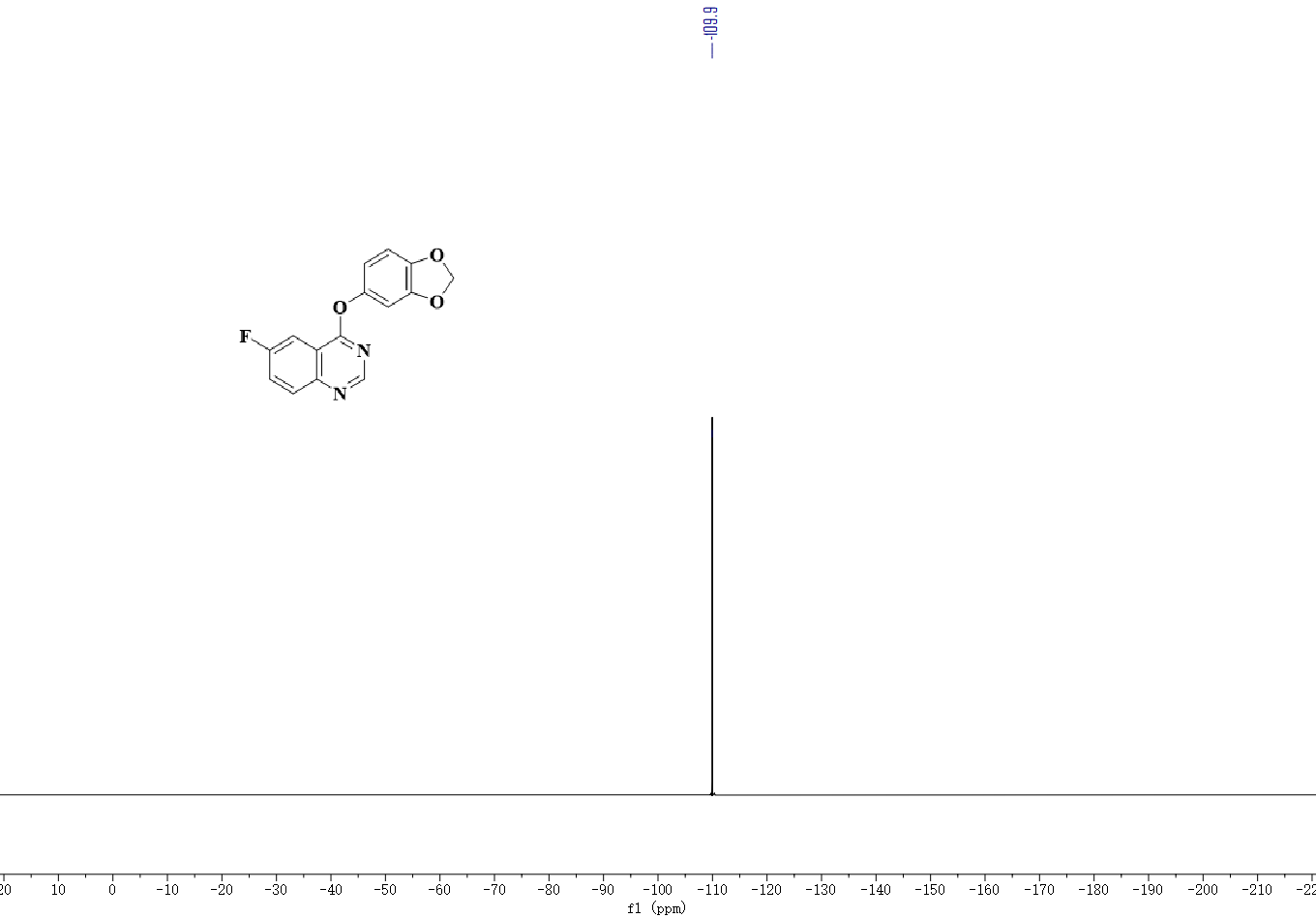


^19^F NMR of Compound 4i


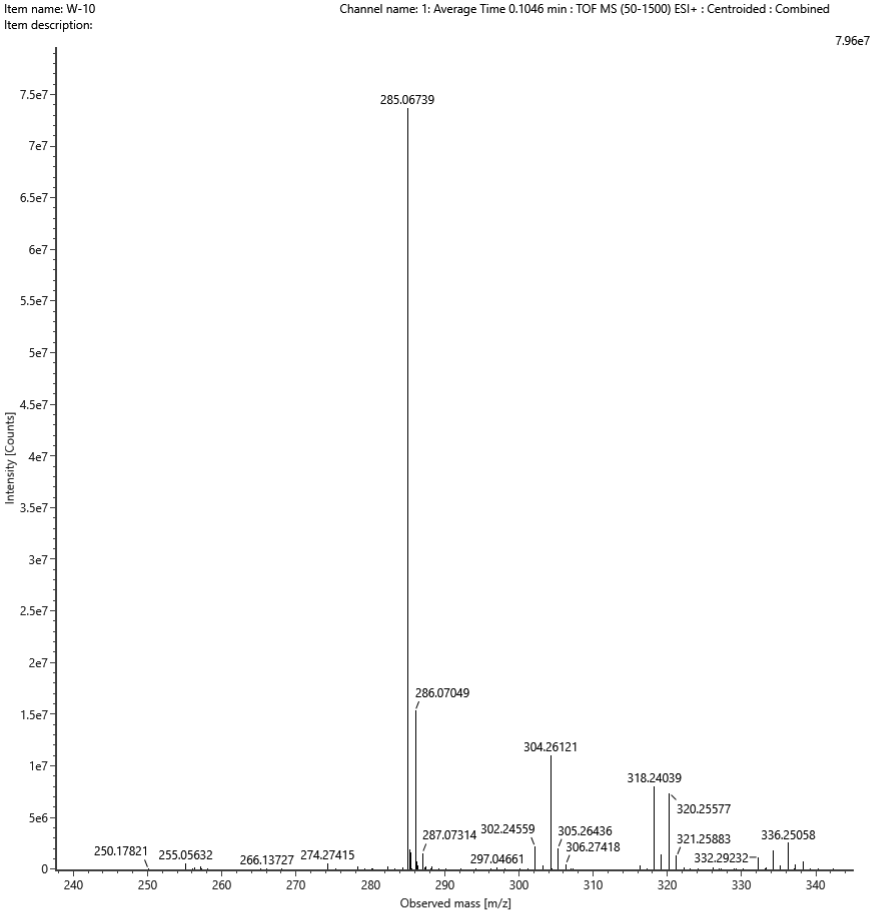


HRMS of Compound 4i


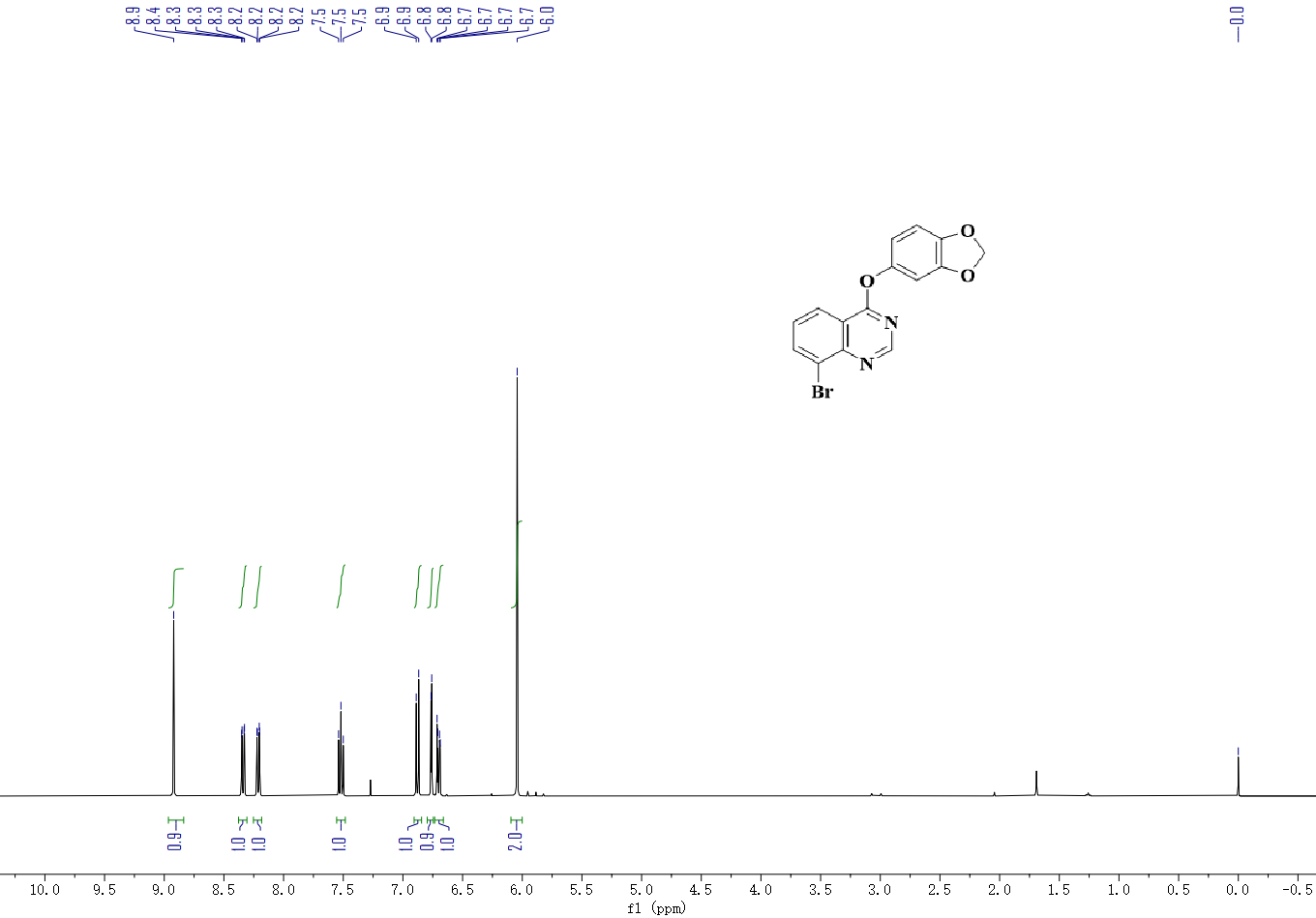


^1^H NMR of Compound 4j


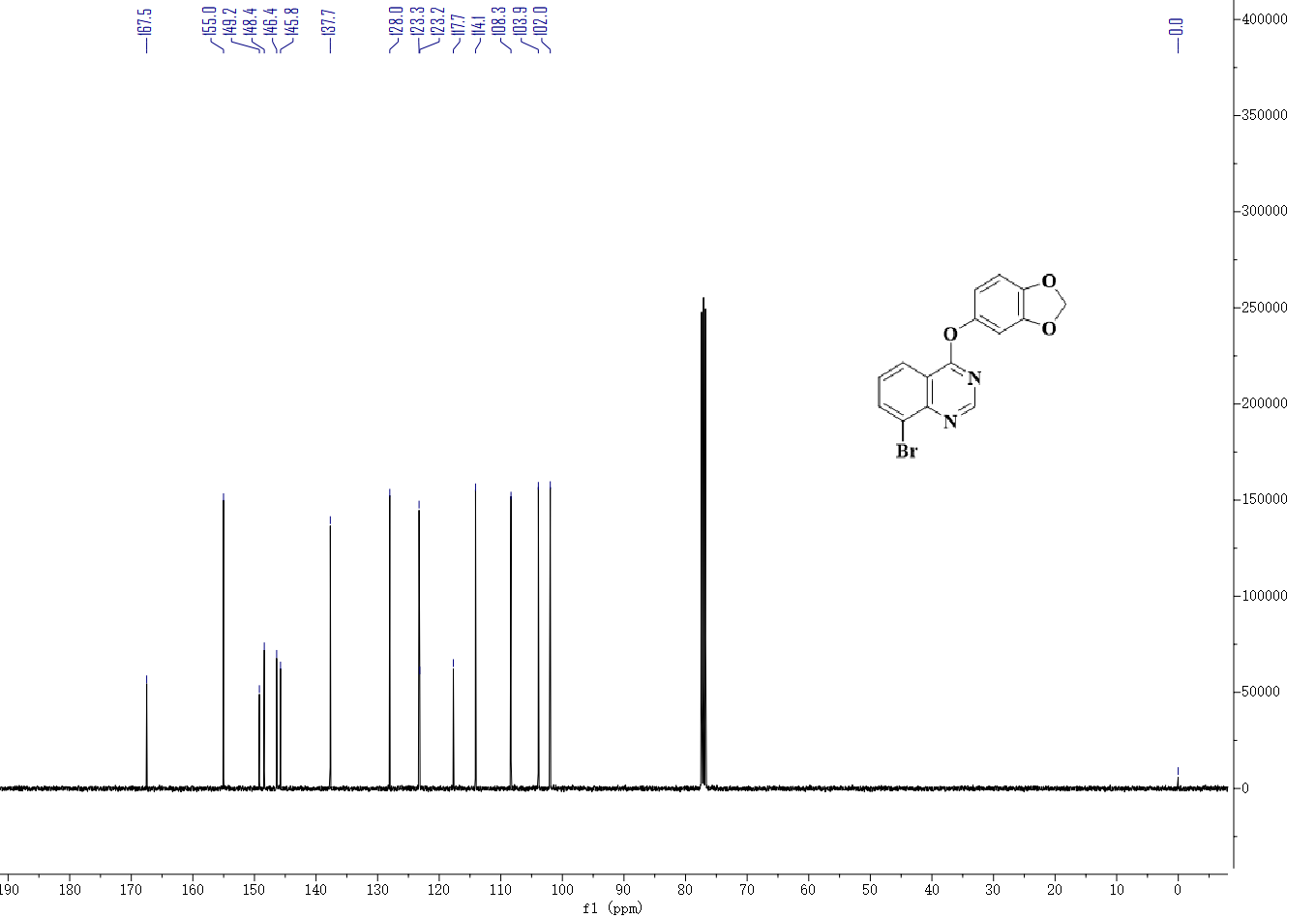


^13^C NMR of Compound 4j


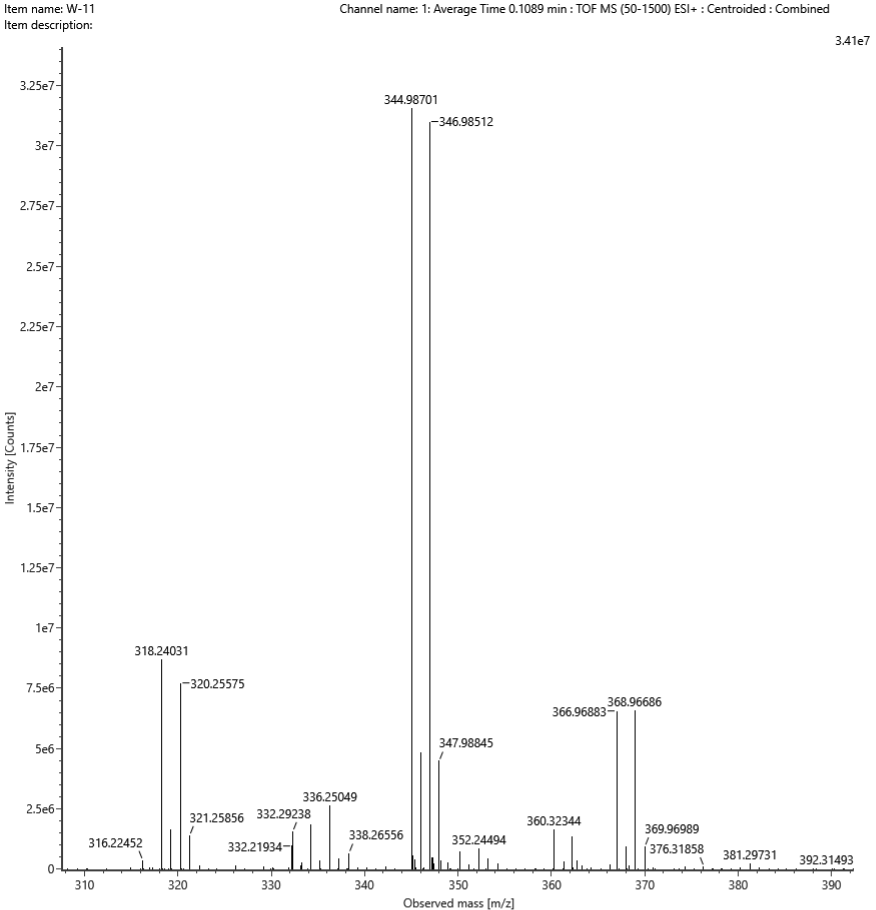


HRMS of Compound 4j


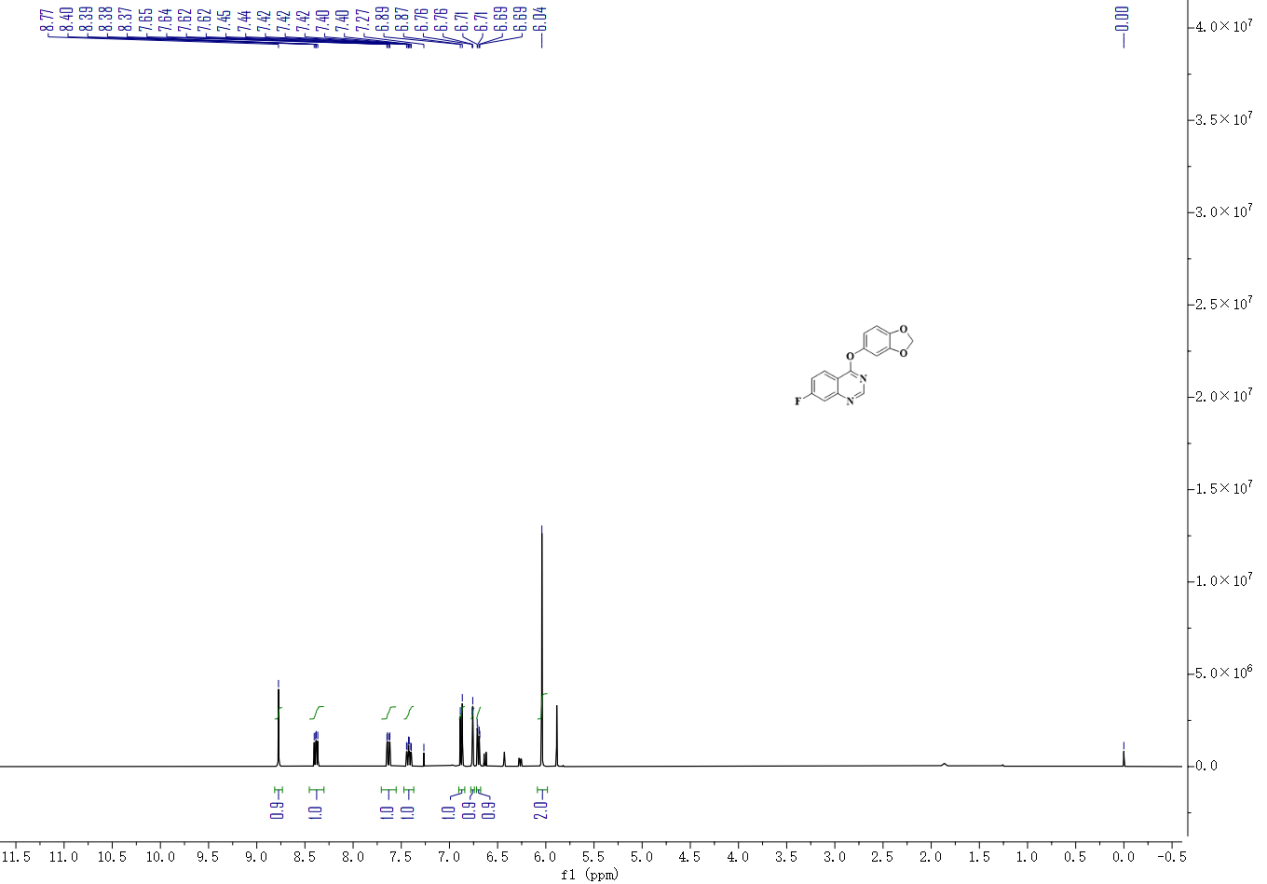


^1^H NMR of Compound 4k


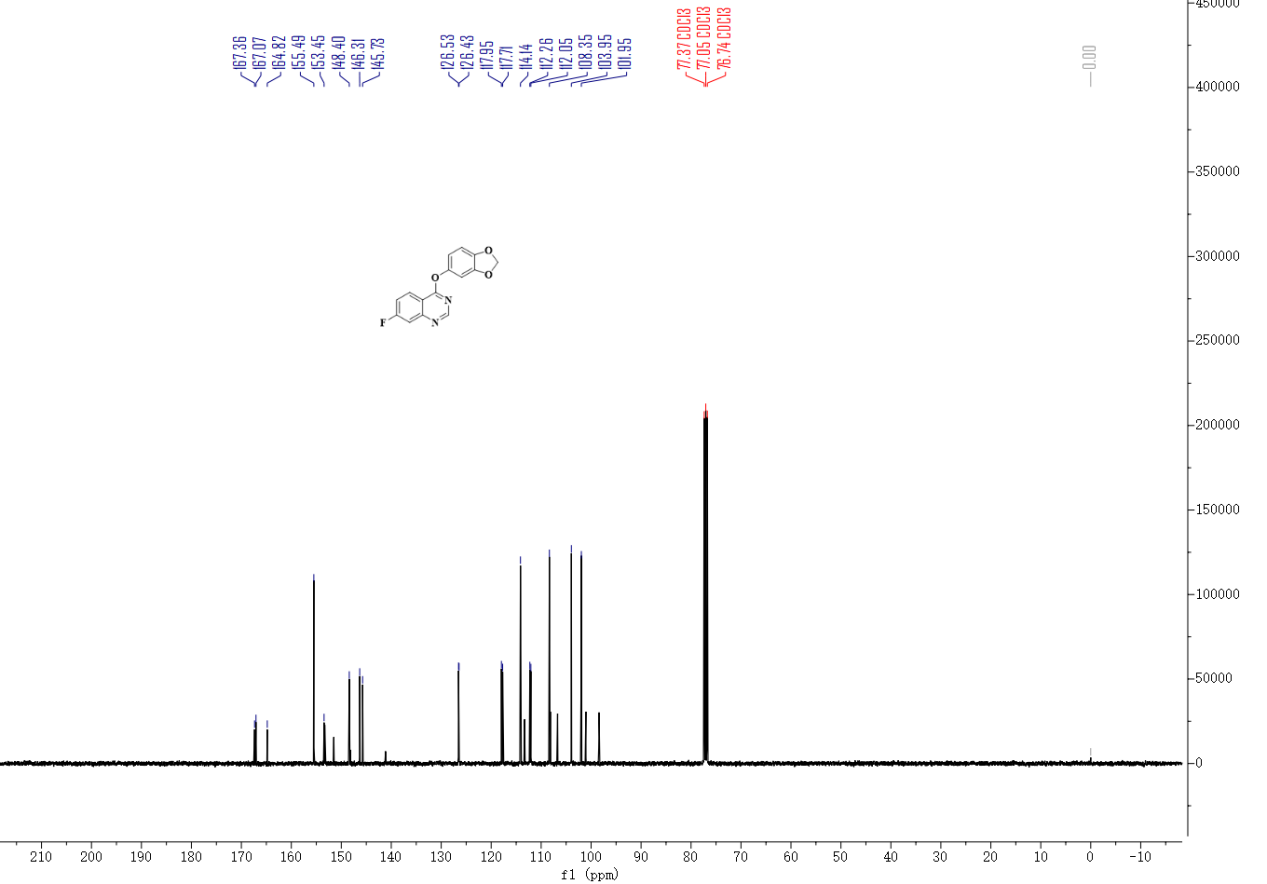


^13^C NMR of Compound 4k


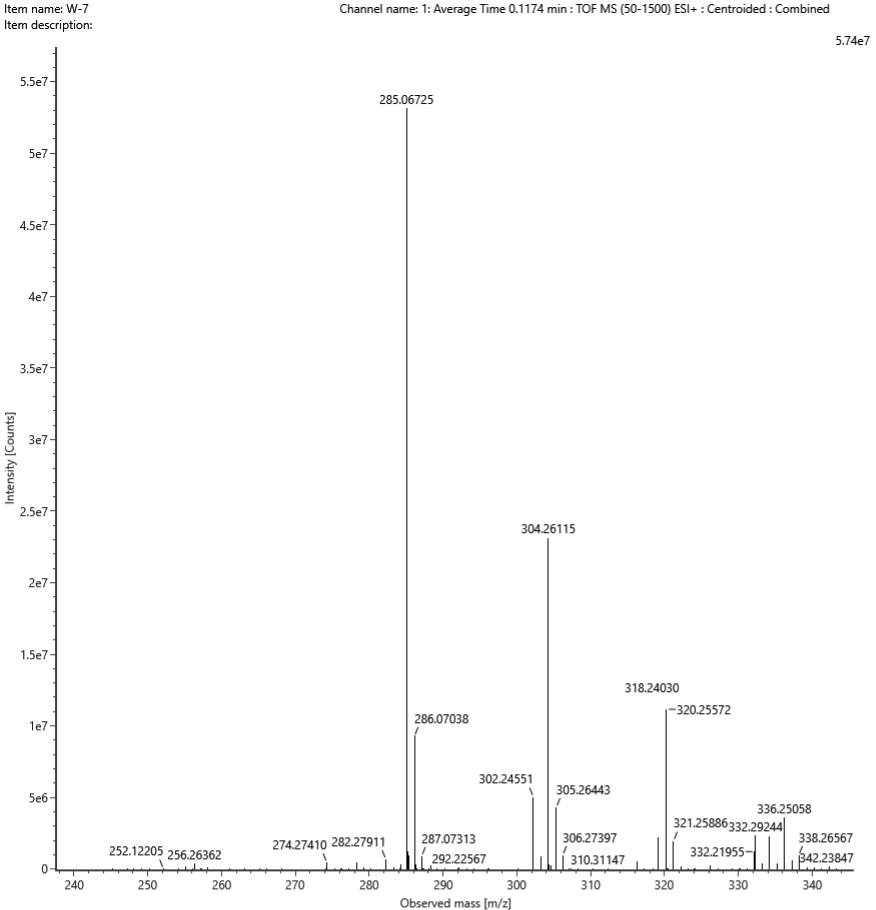


HRMS of Compound 4k


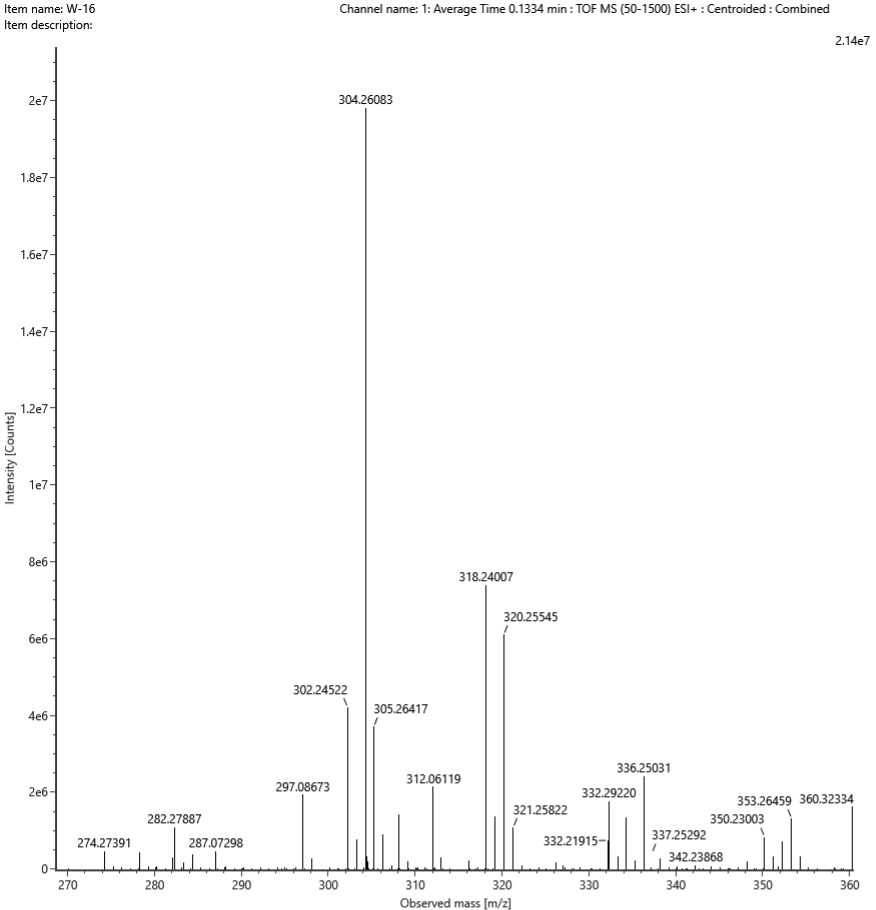


HRMS of Compound 4l


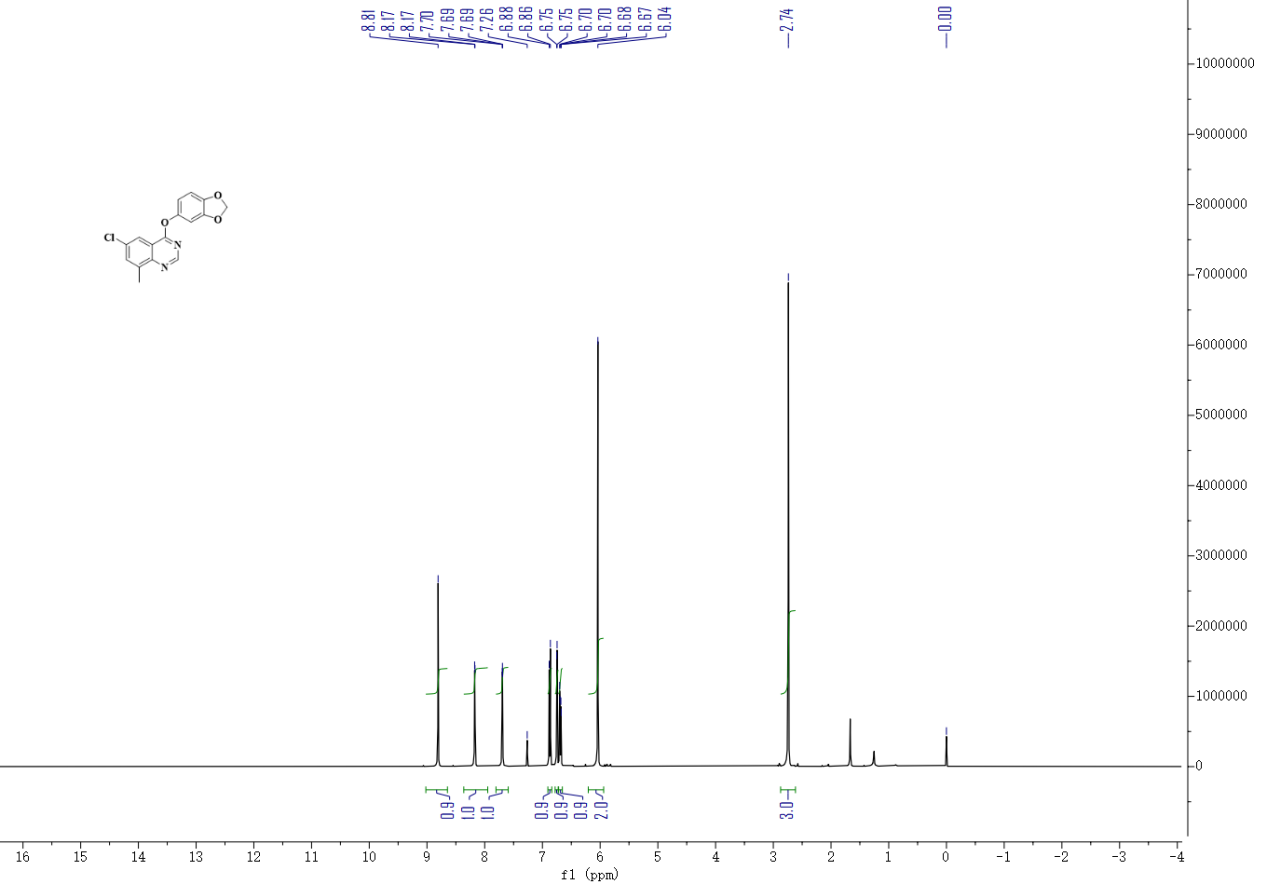


^1^H NMR of Compound 4m


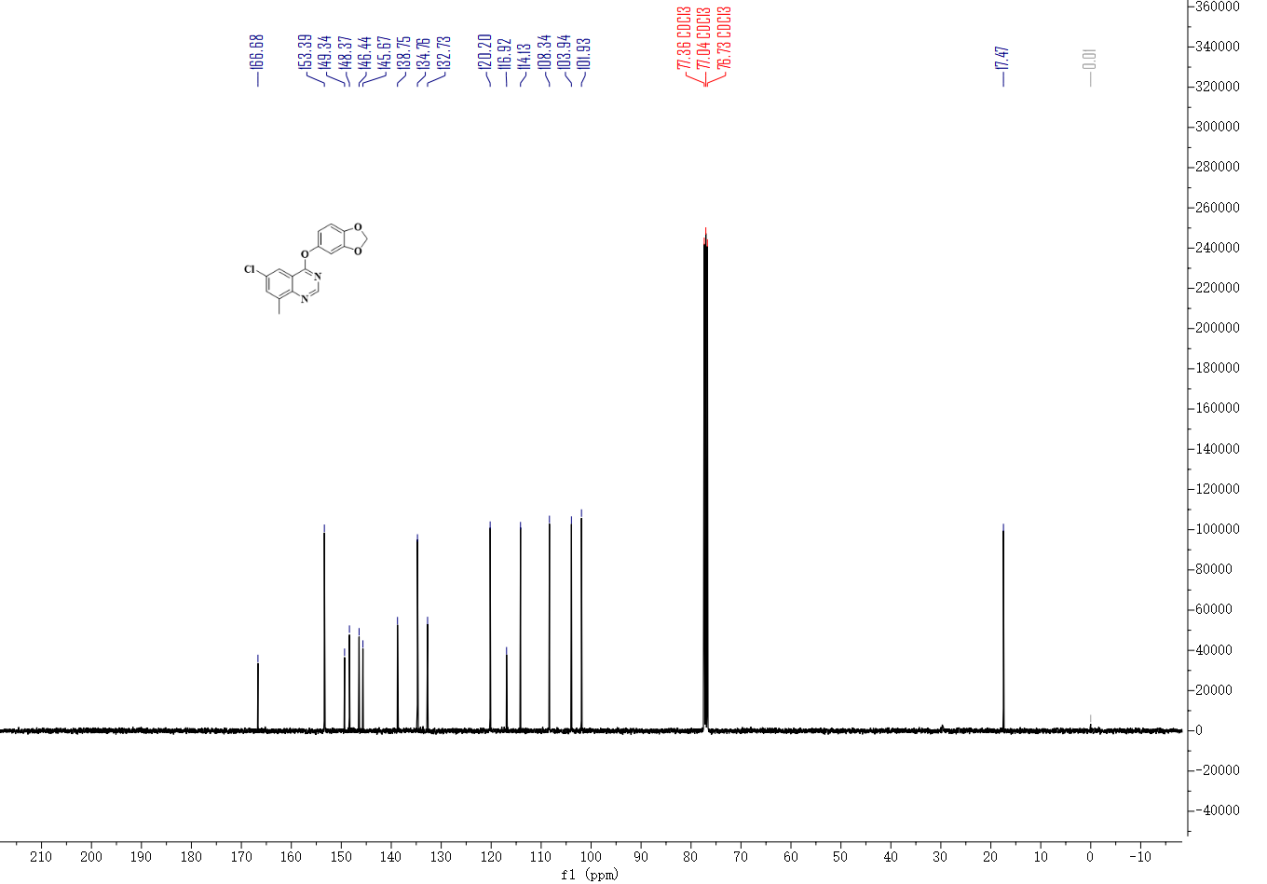


^13^C NMR of Compound 4m


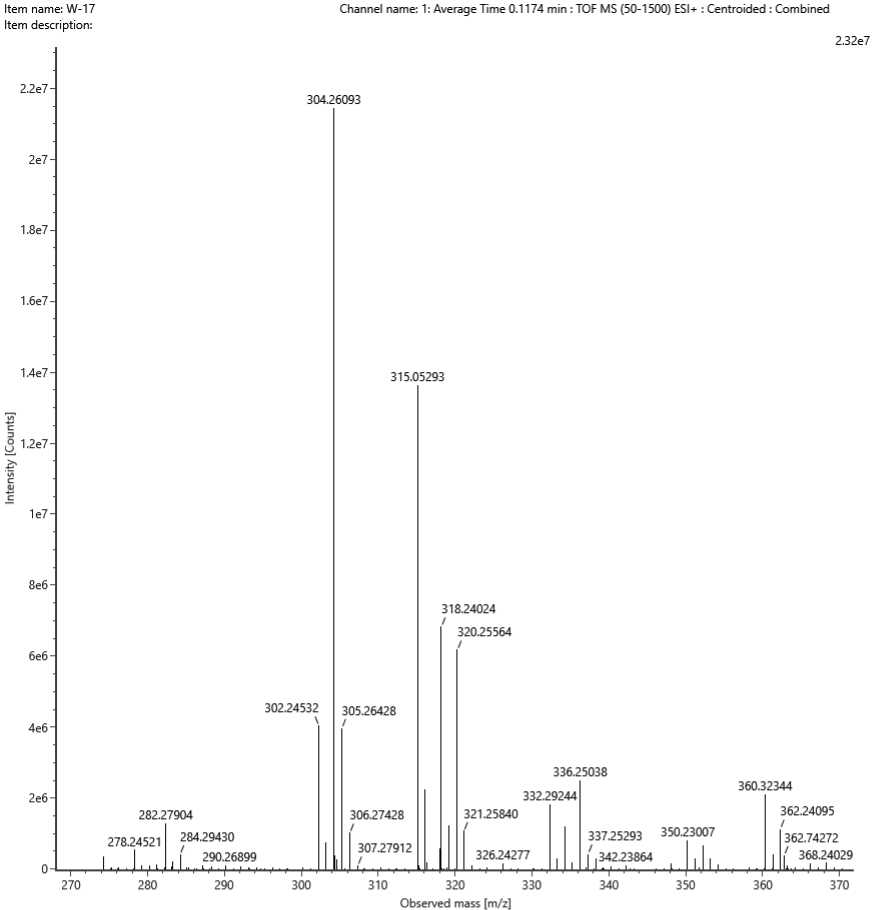


HRMS of Compound 4m


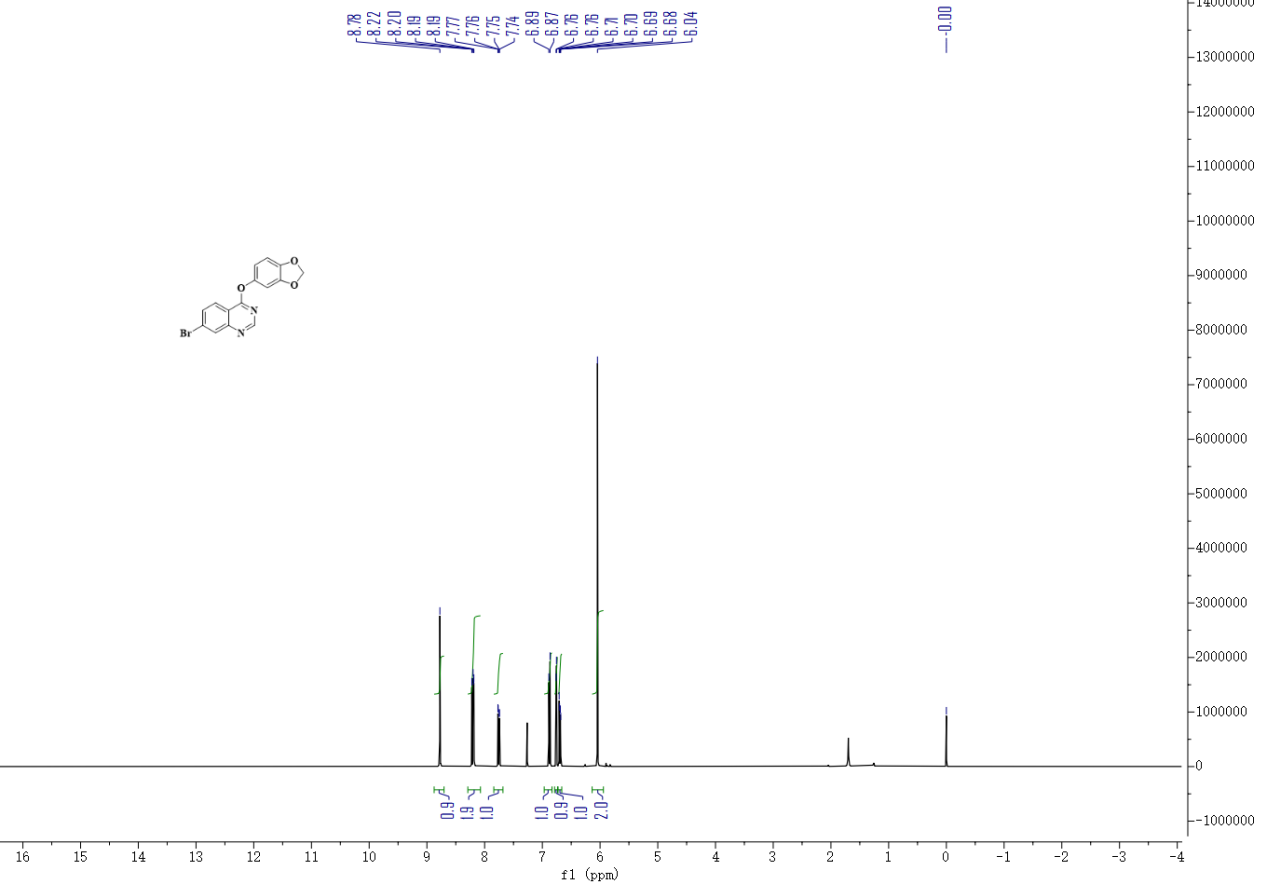


^1^H NMR of Compound 4n


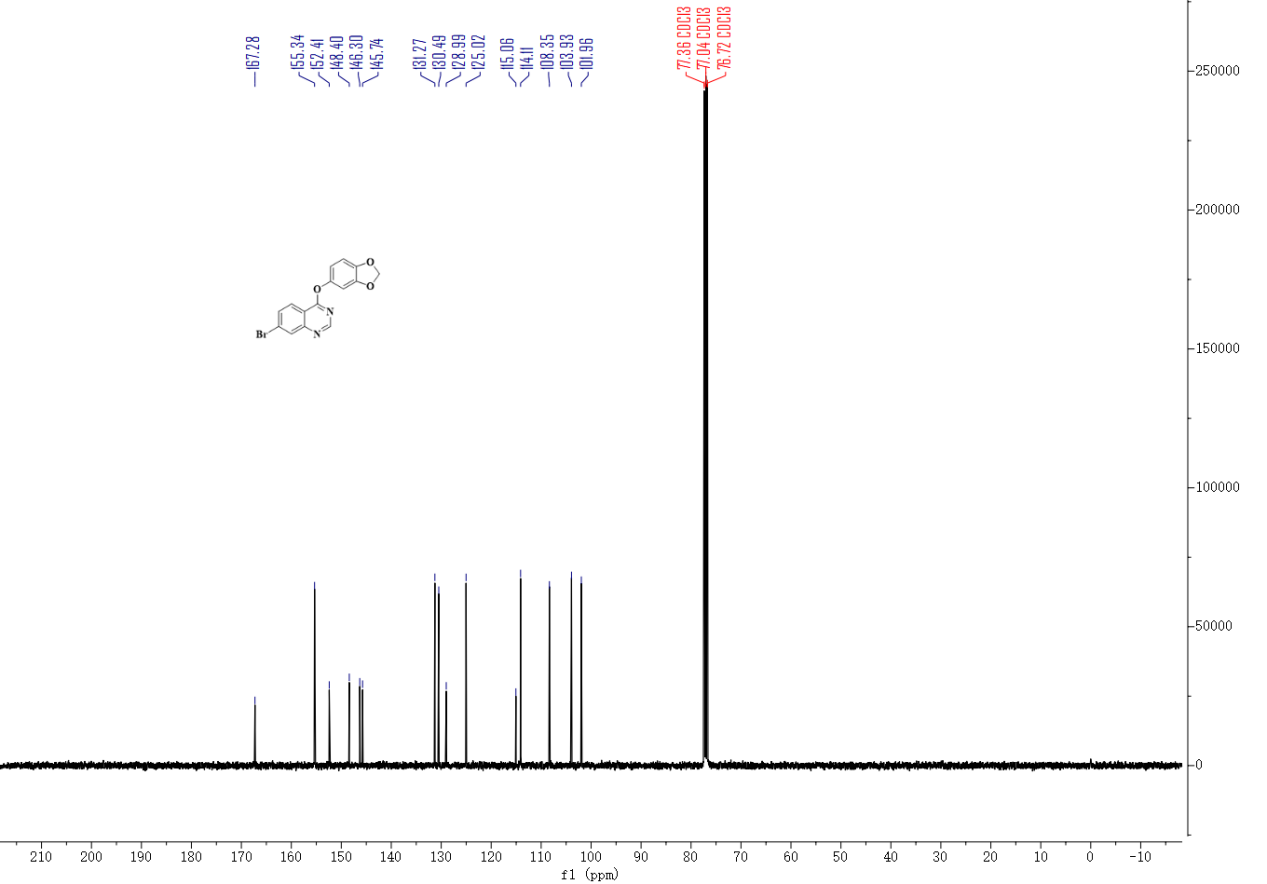


^13^C NMR of Compound 4n


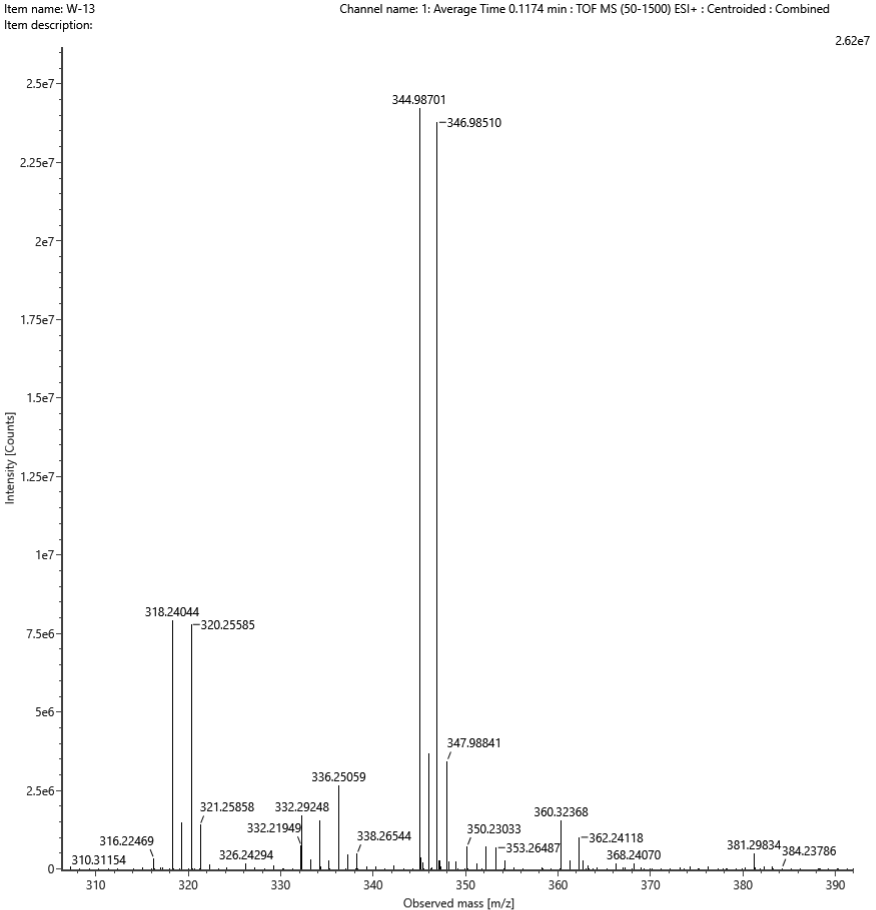


HRMS of Compound 4n


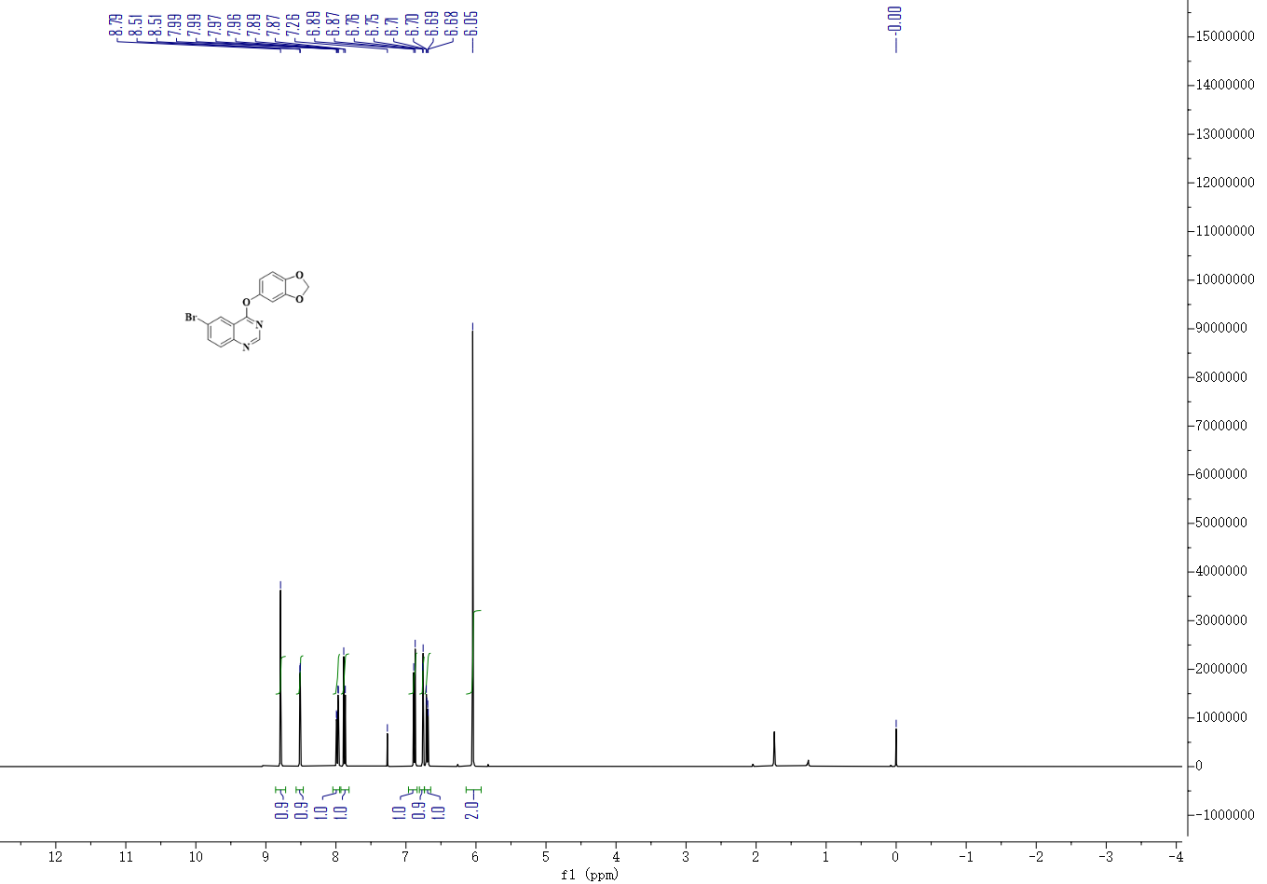


^1^H NMR of Compound 4o


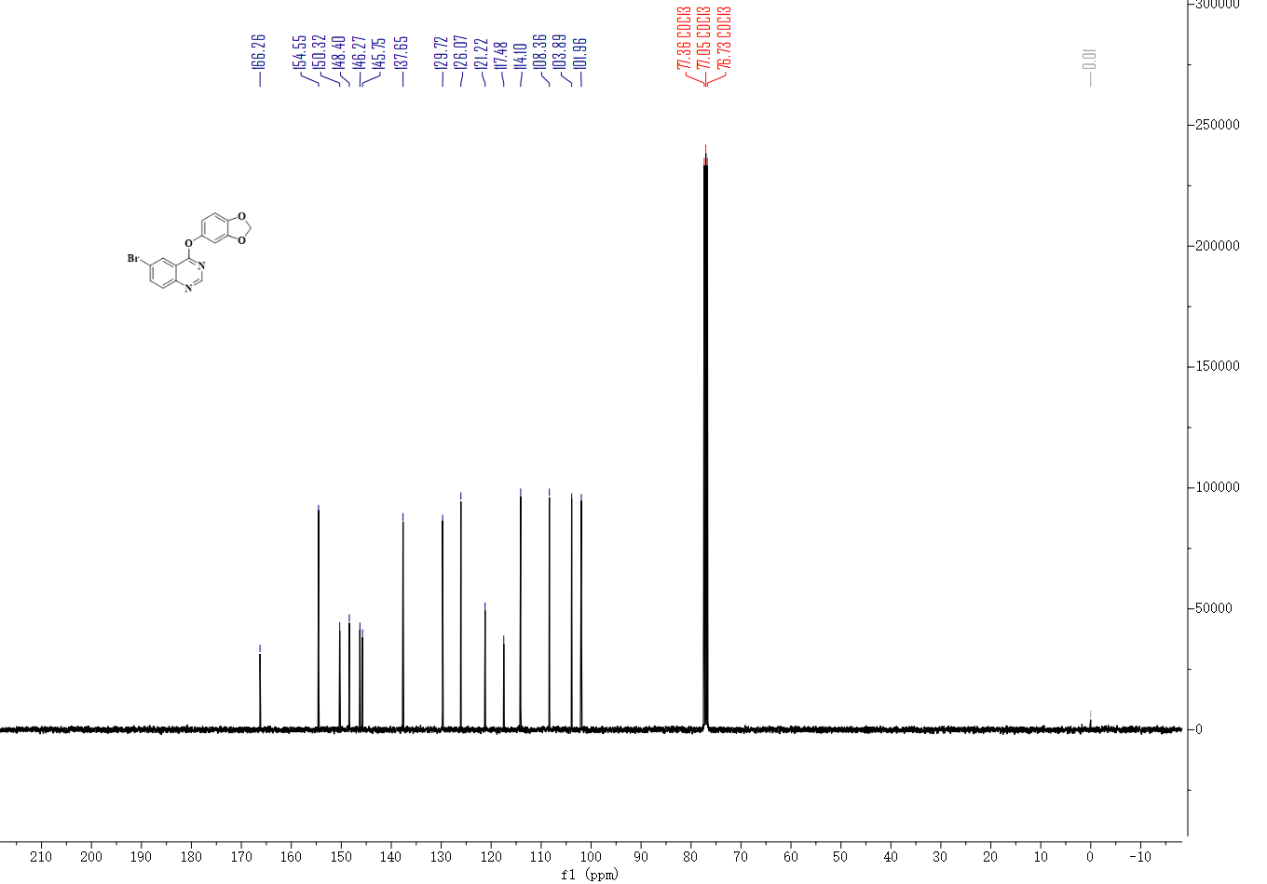


^13^C NMR of Compound 4o


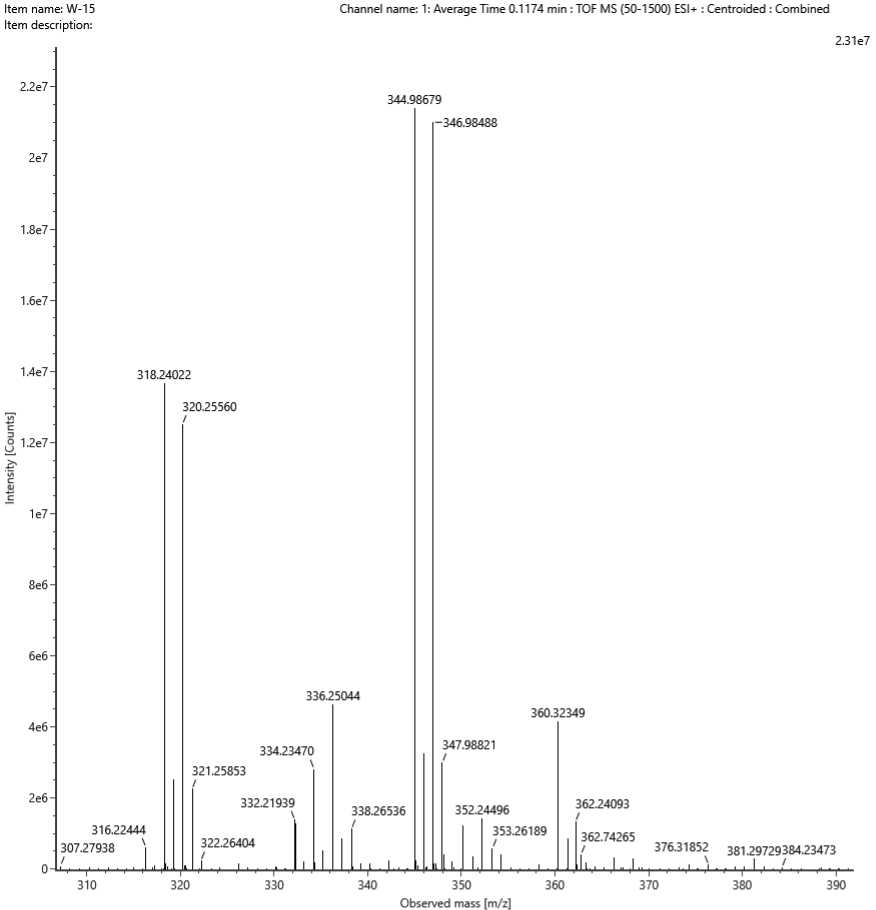


HRMS of Compound 4o


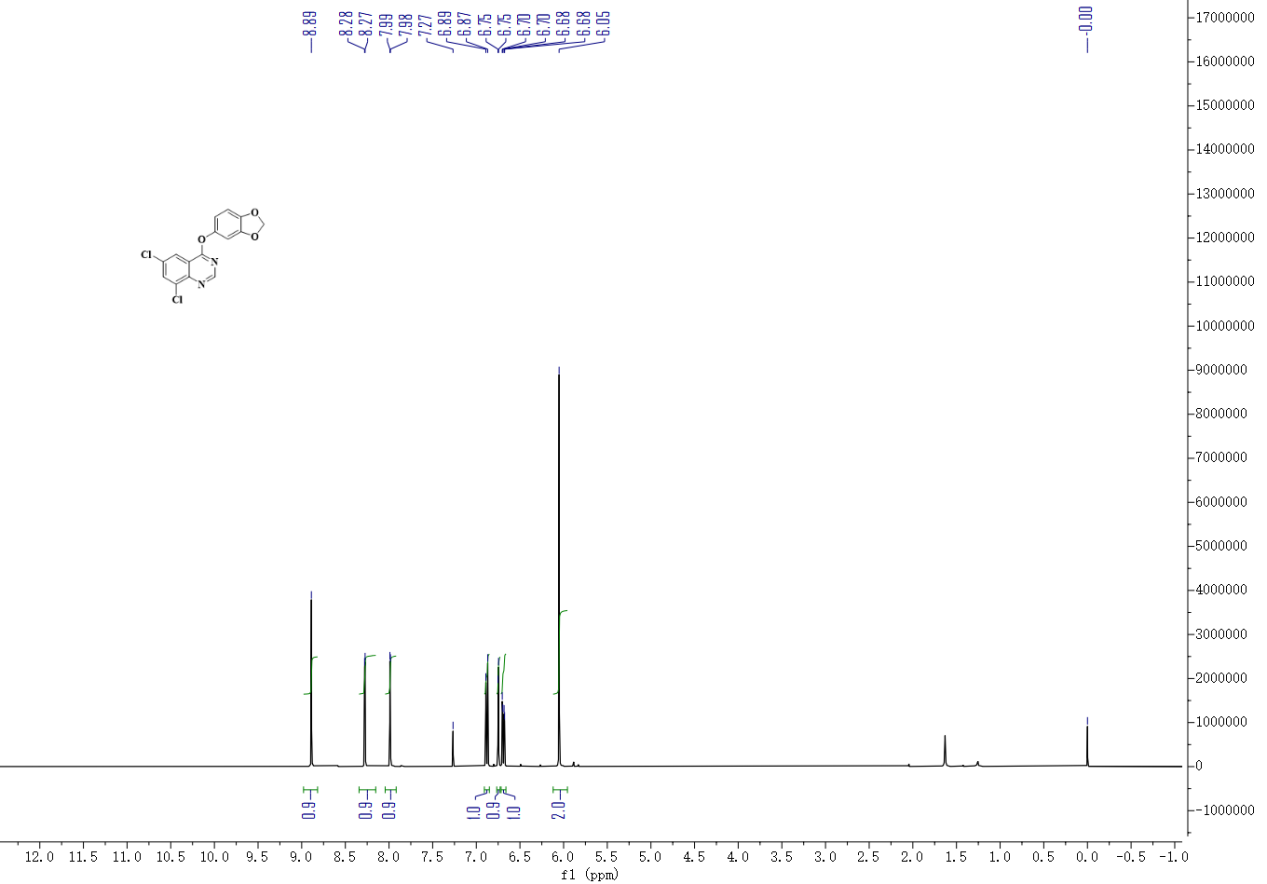


^1^H NMR of Compound 4p


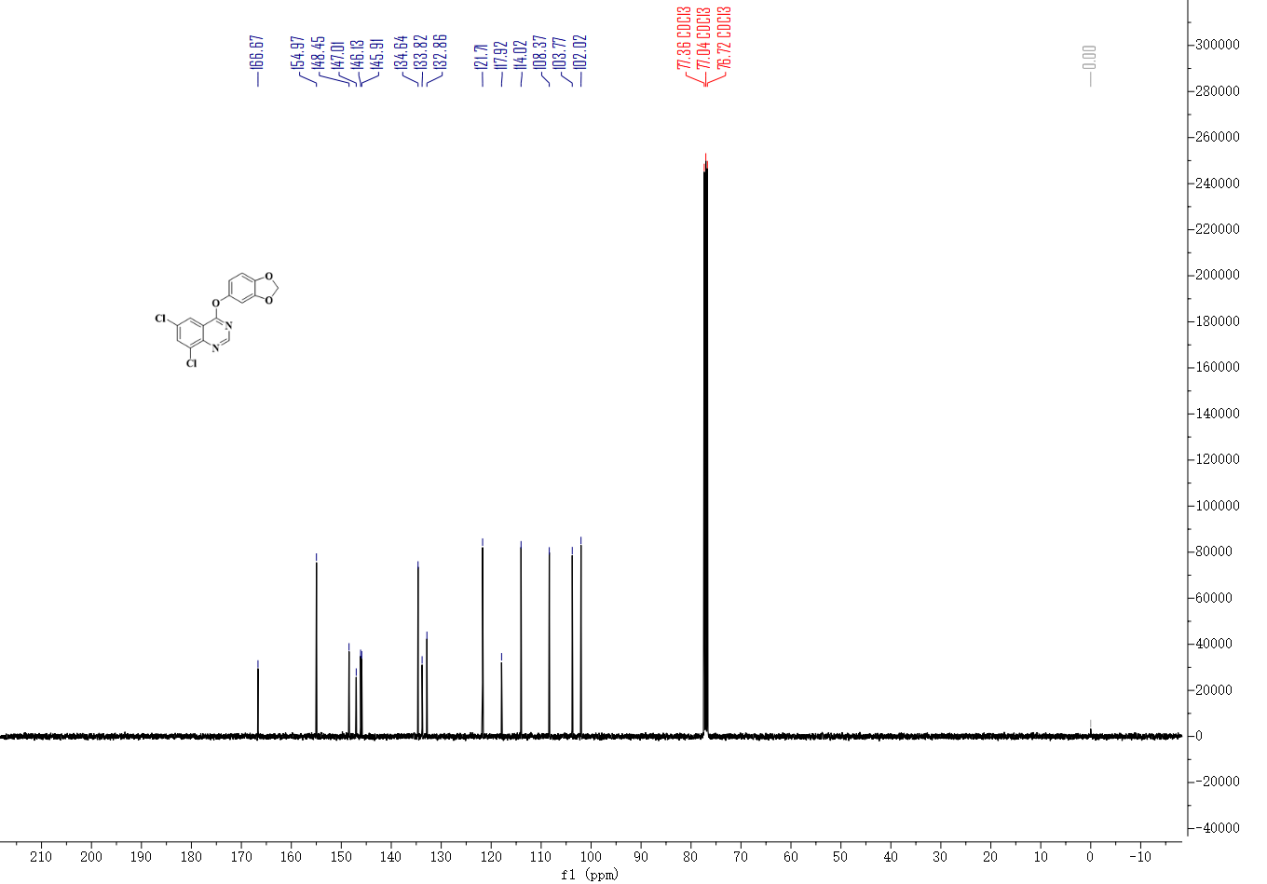


^13^C NMR of Compound 4p


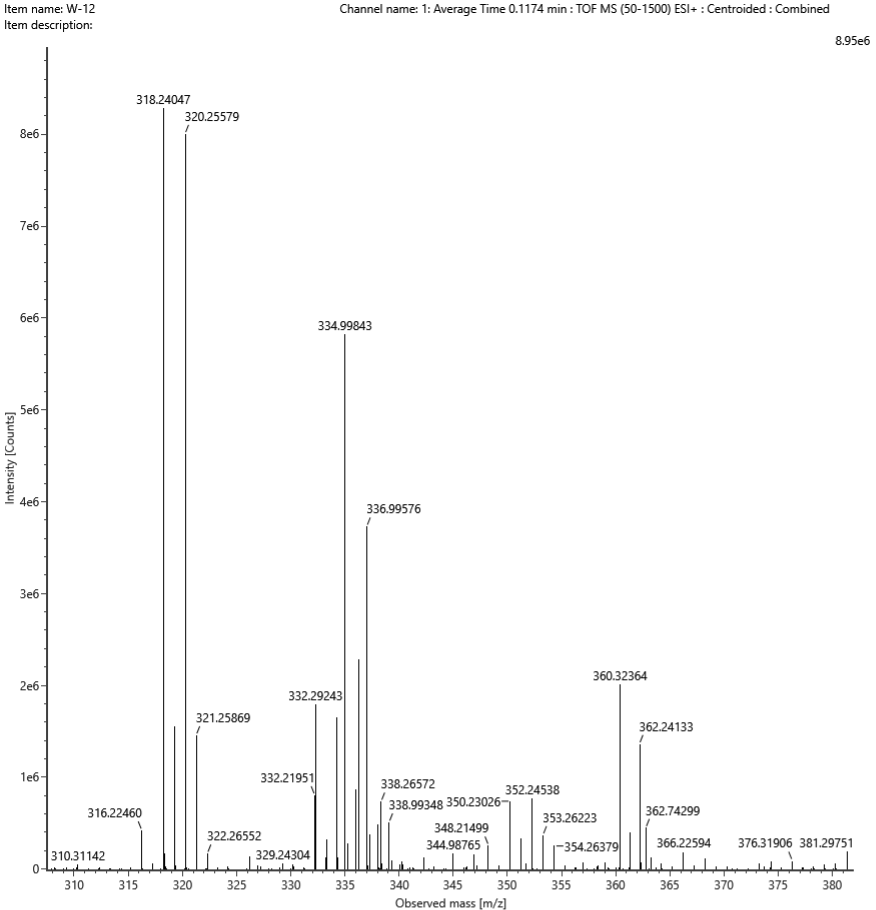


HRMS of Compound 4p


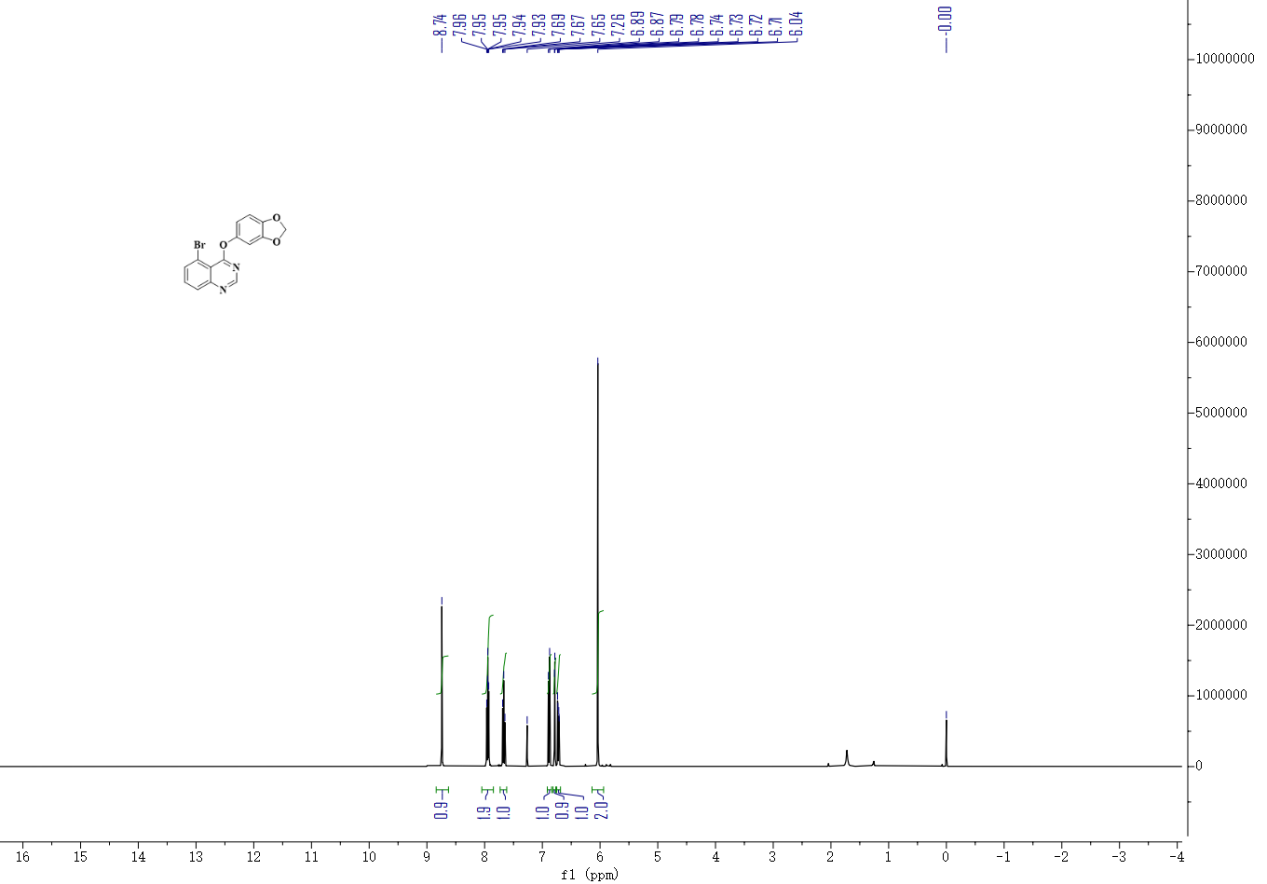


^1^H NMR of Compound 4q


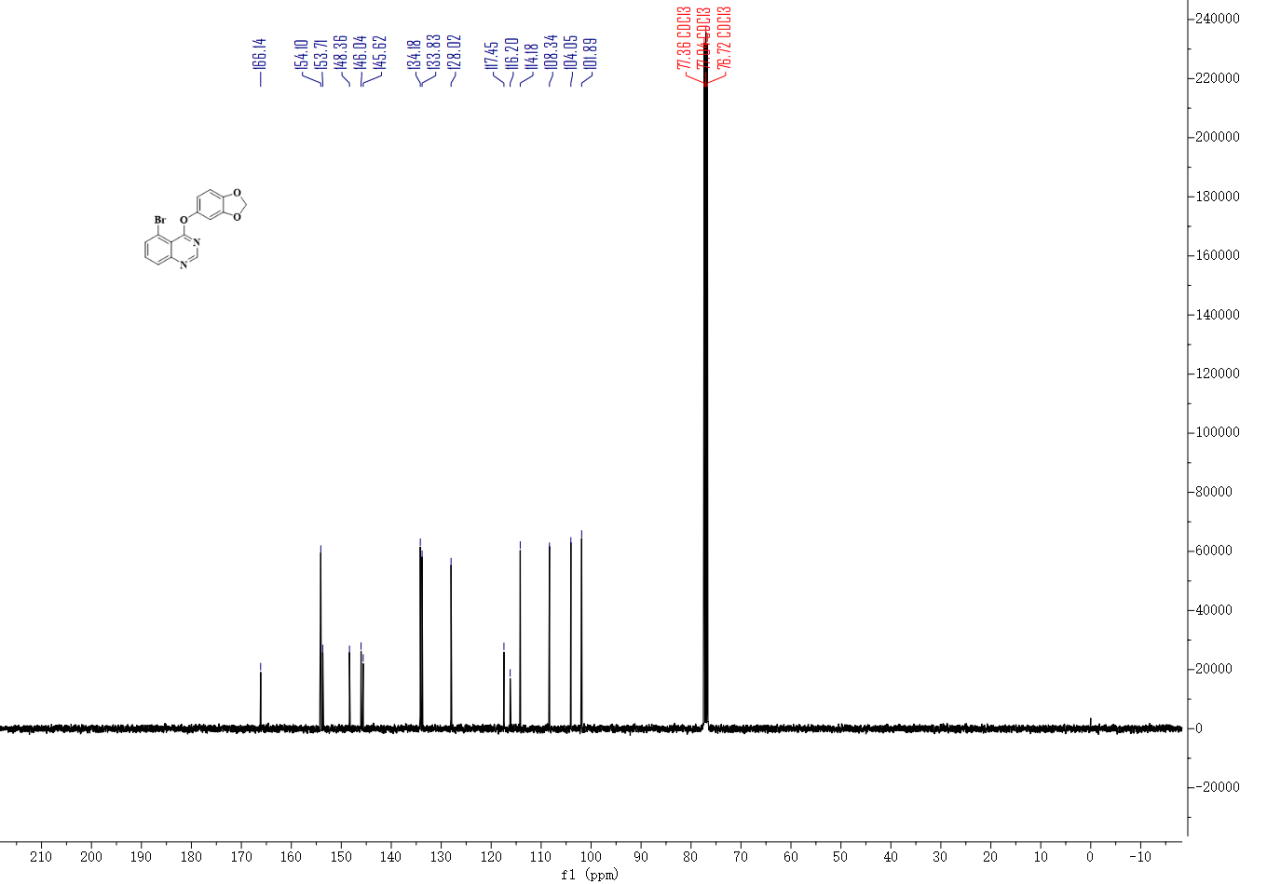


^13^C NMR of Compound 4q


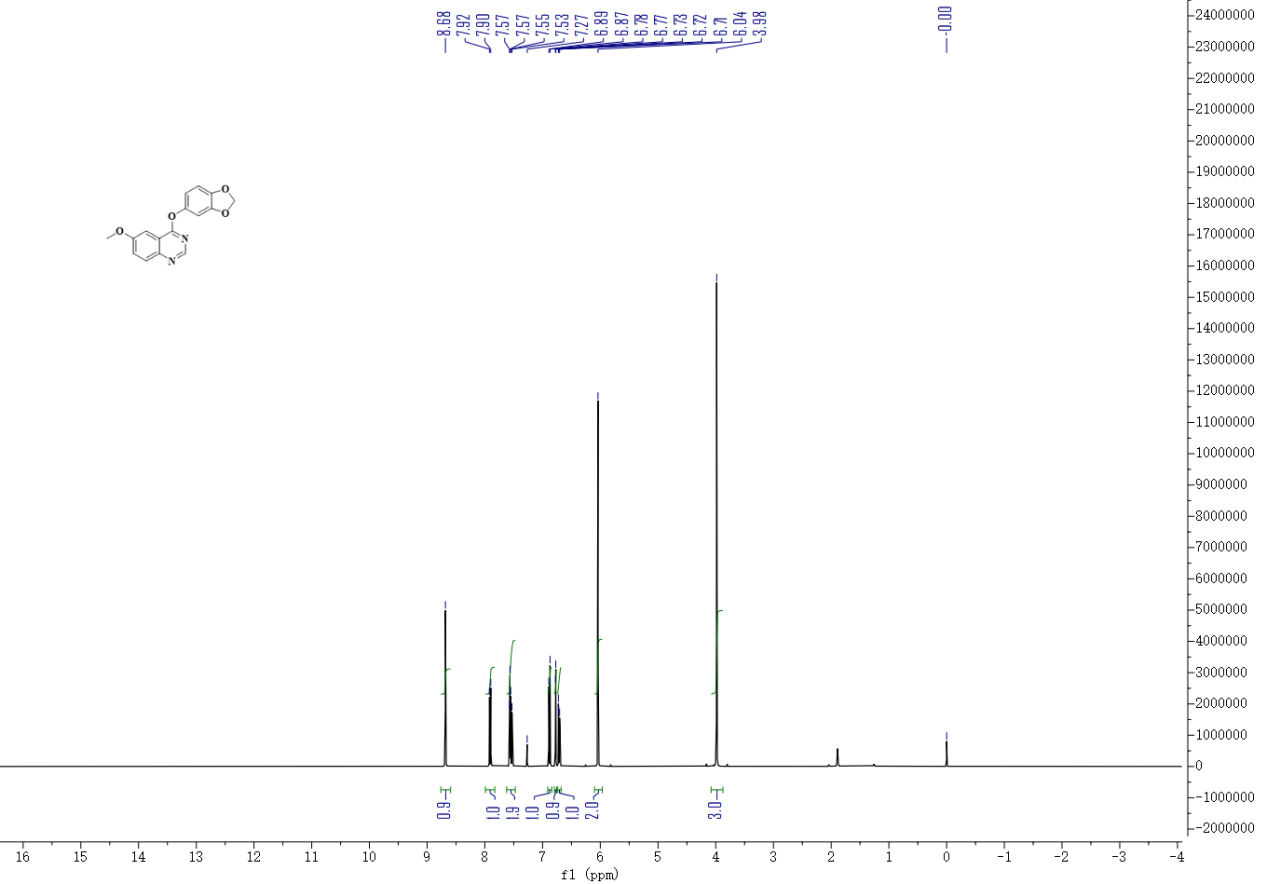


^1^H NMR of Compound 4r


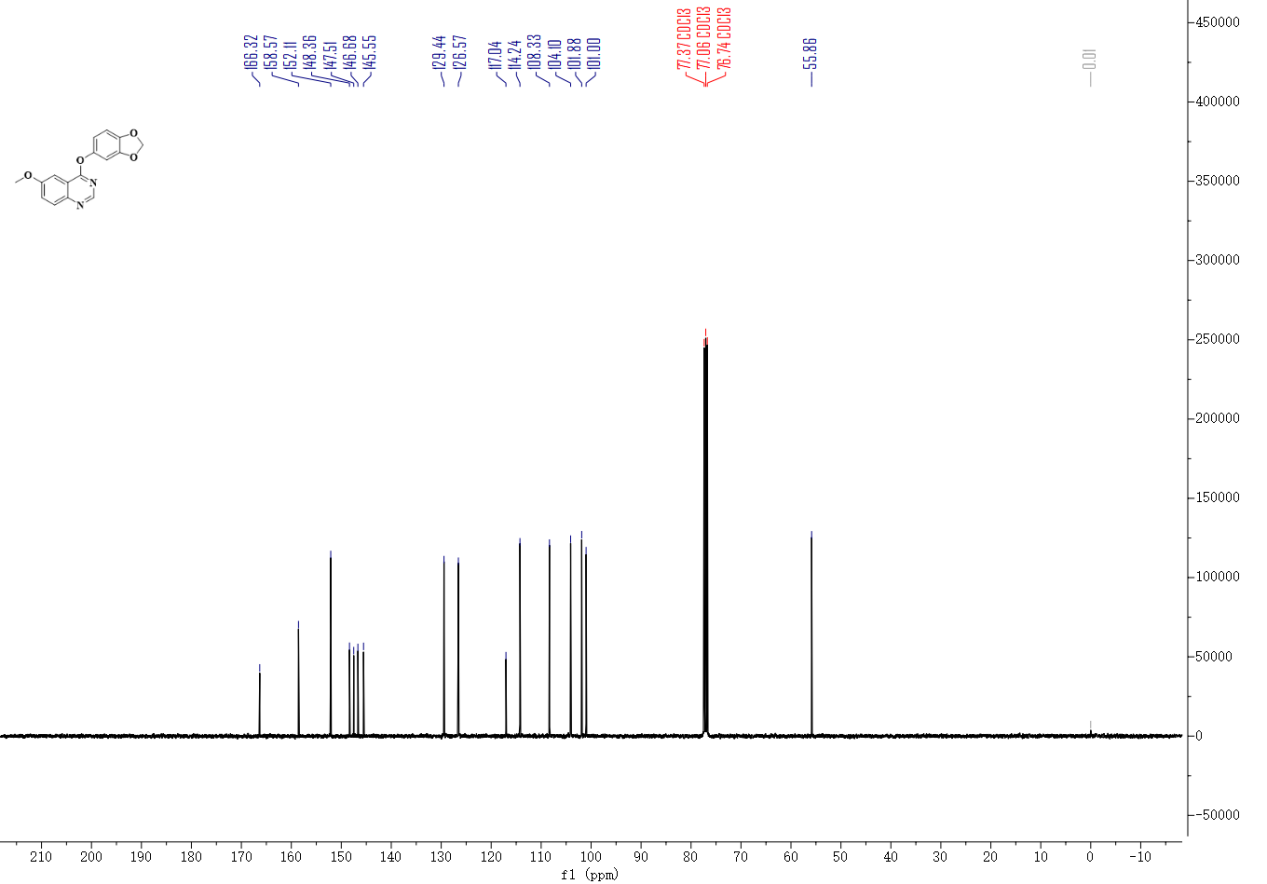


^13^C NMR of Compound 4r


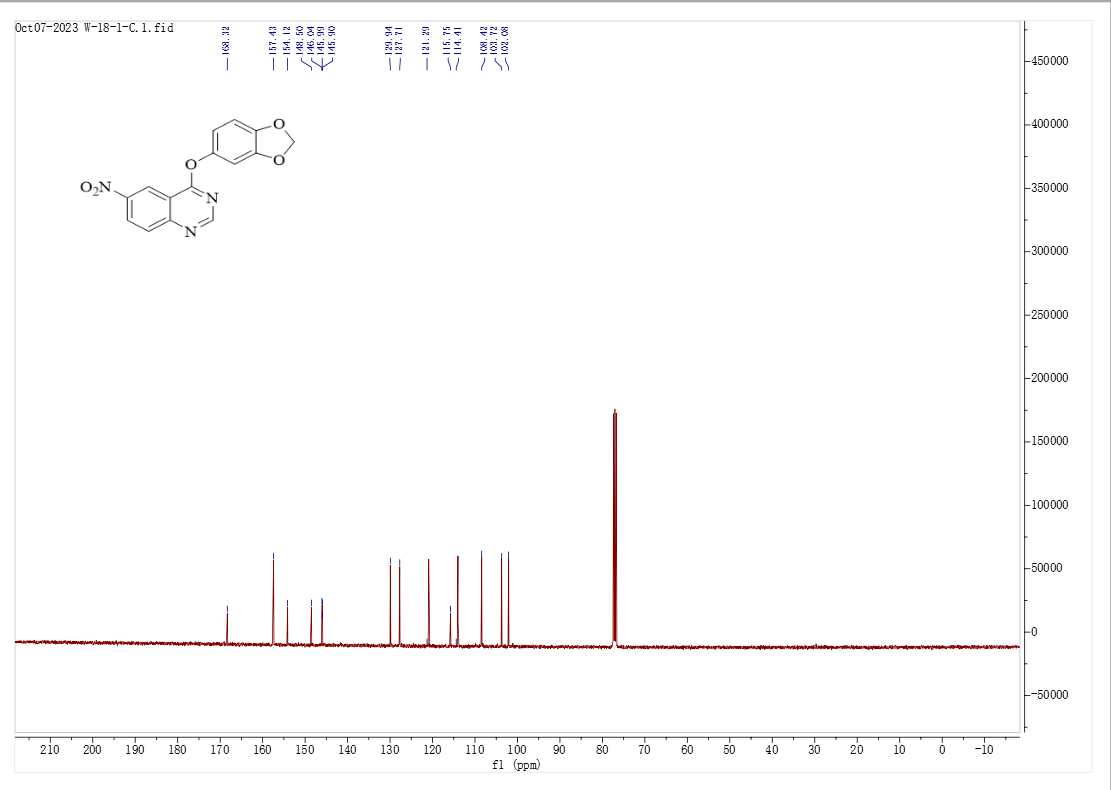


^13^C NMR of Compound 4s


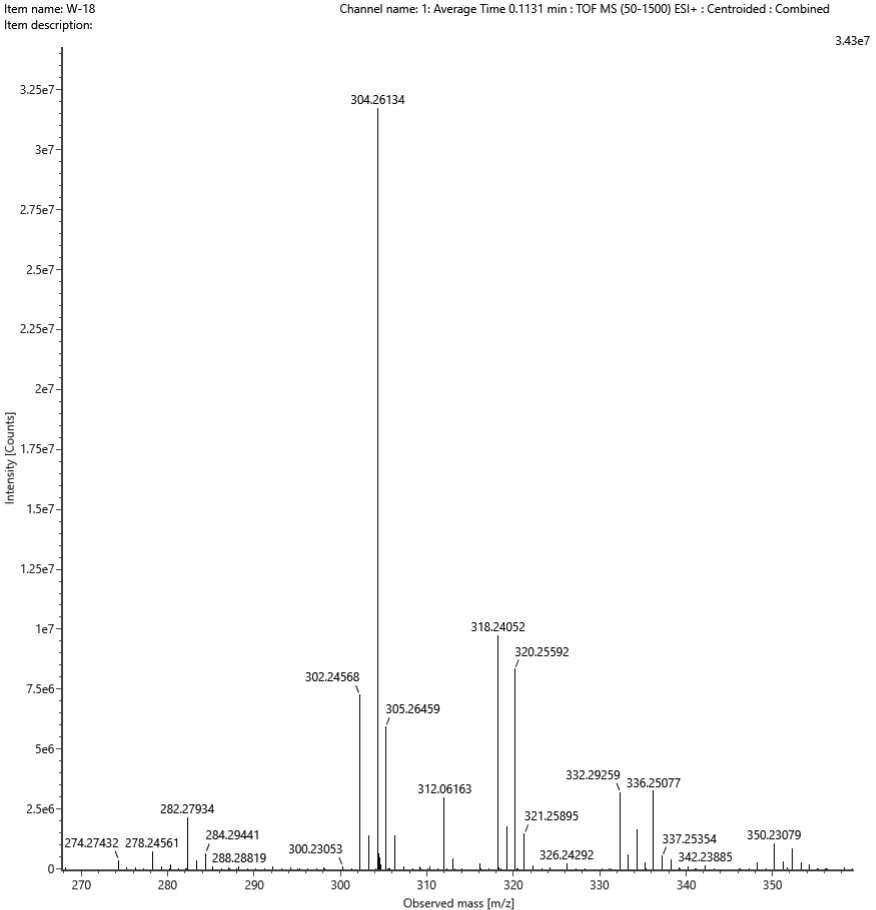


HRMS of Compound 4s


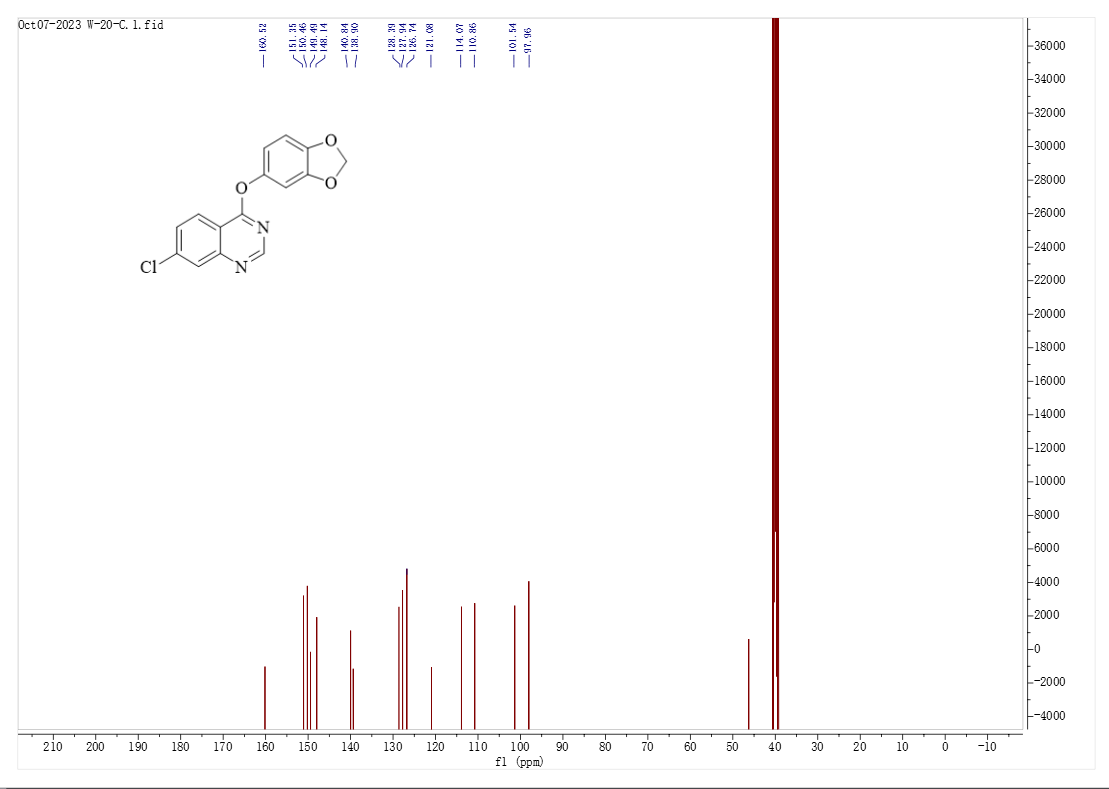


^13^C NMR of Compound 4t


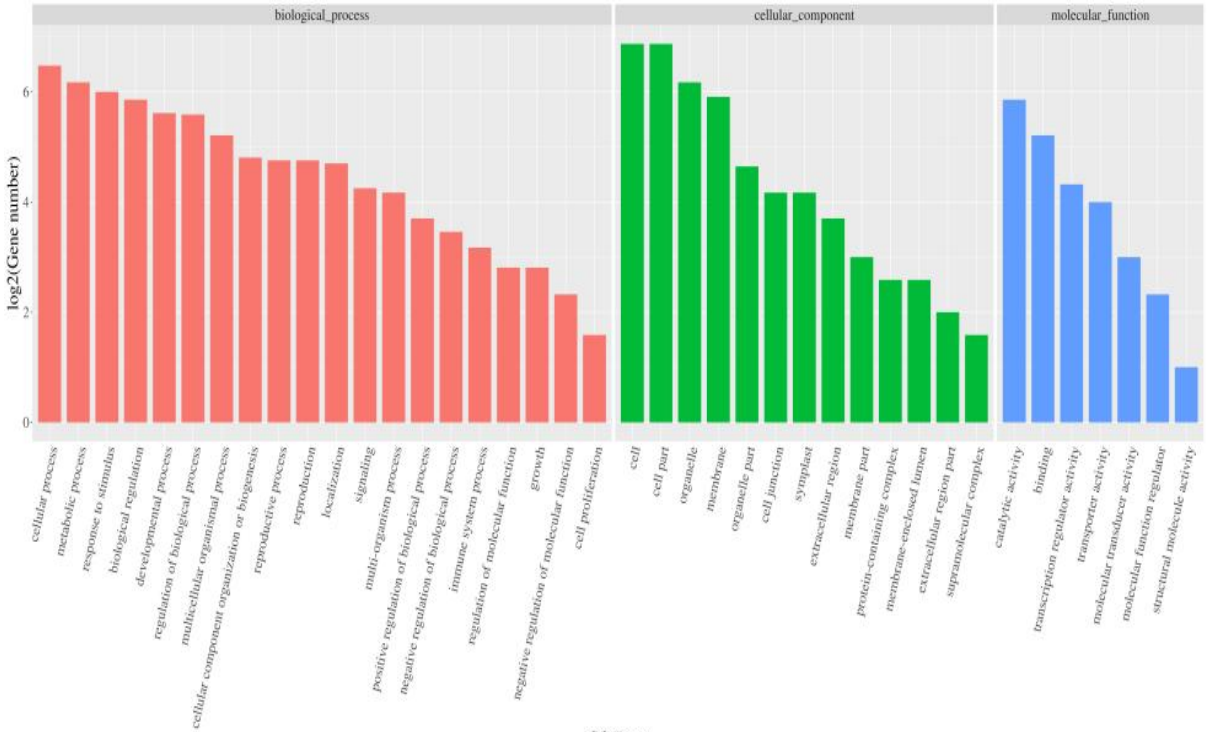


**Fig. 1** GO functional enrichment analysis of DEGs


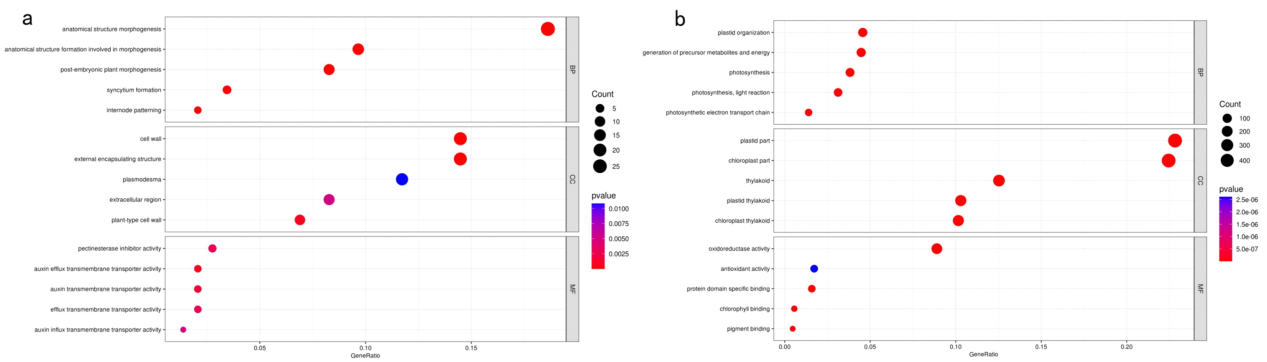


**Fig. 2** Bubble plot of GO functional enrichment for DEGs


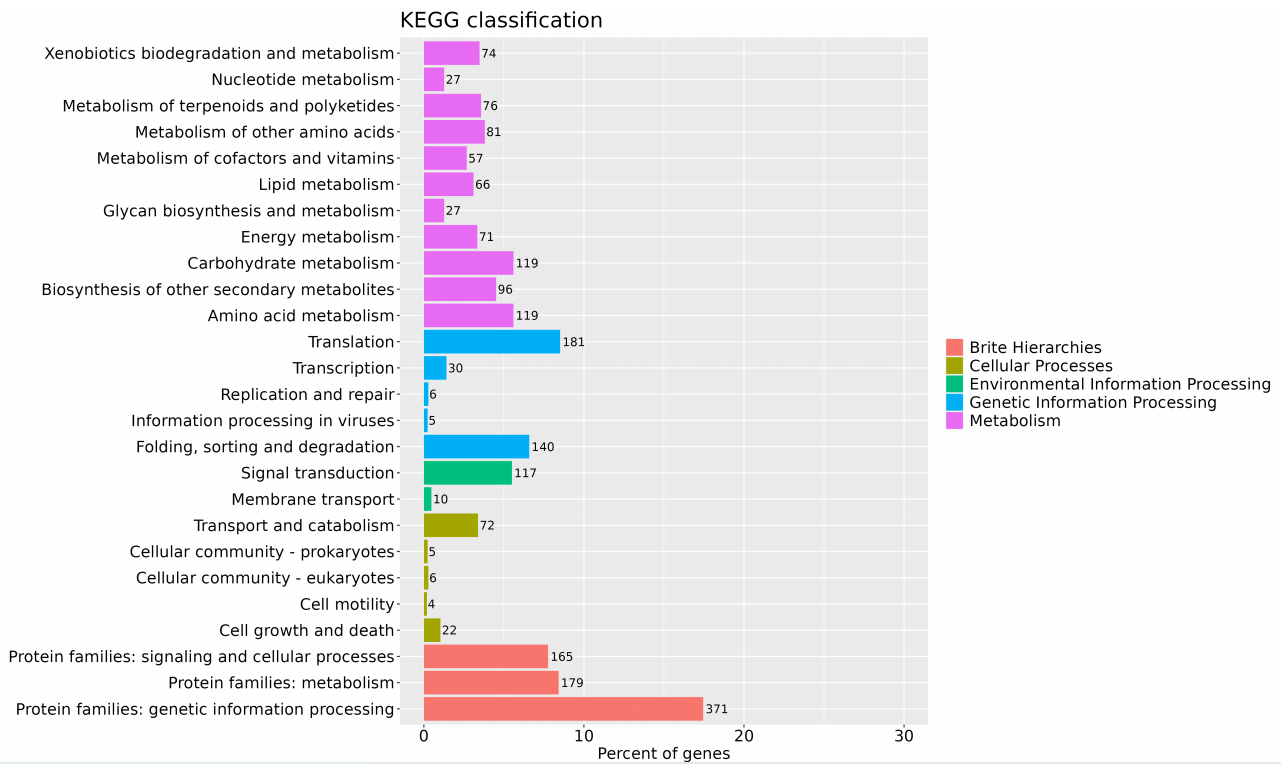


**Fig. 3** KEGG annotation classification statistics chart


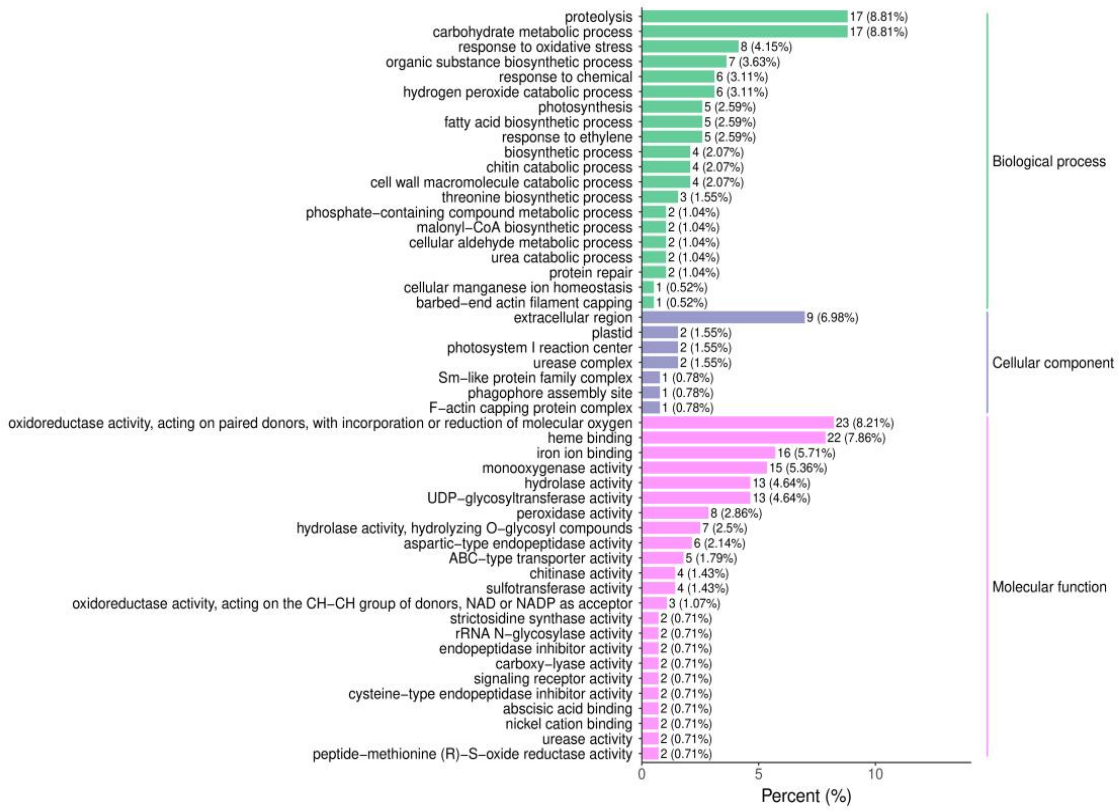


**Fig. 4** GO enrichment analysis of differentially expressed proteins


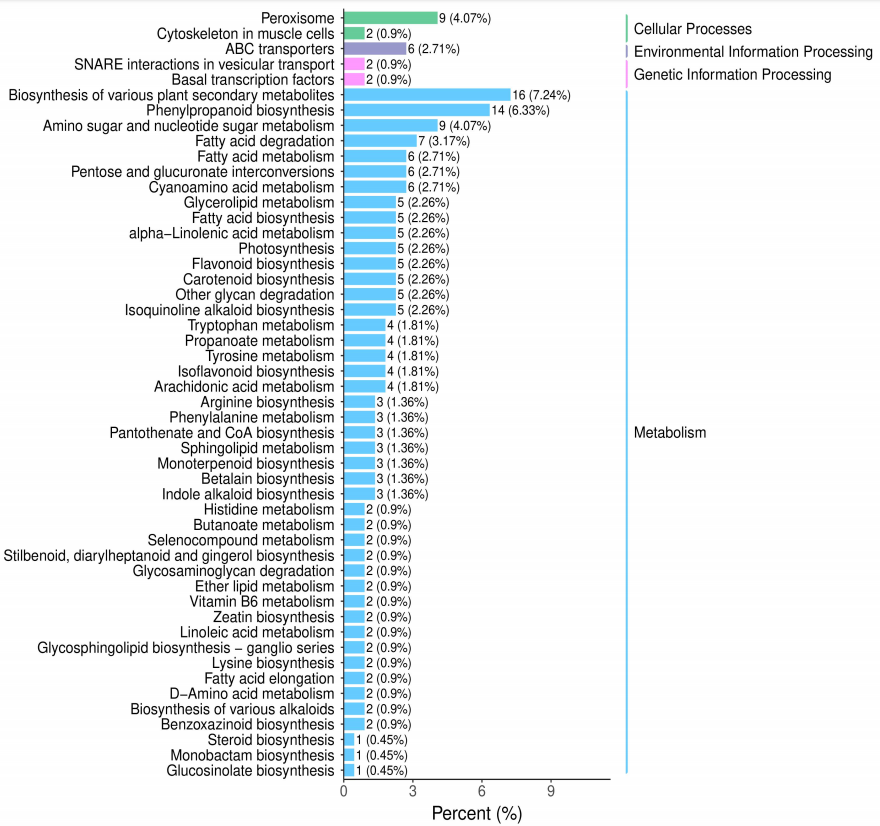


**Fig. 5** GO enrichment analysis of DEPs


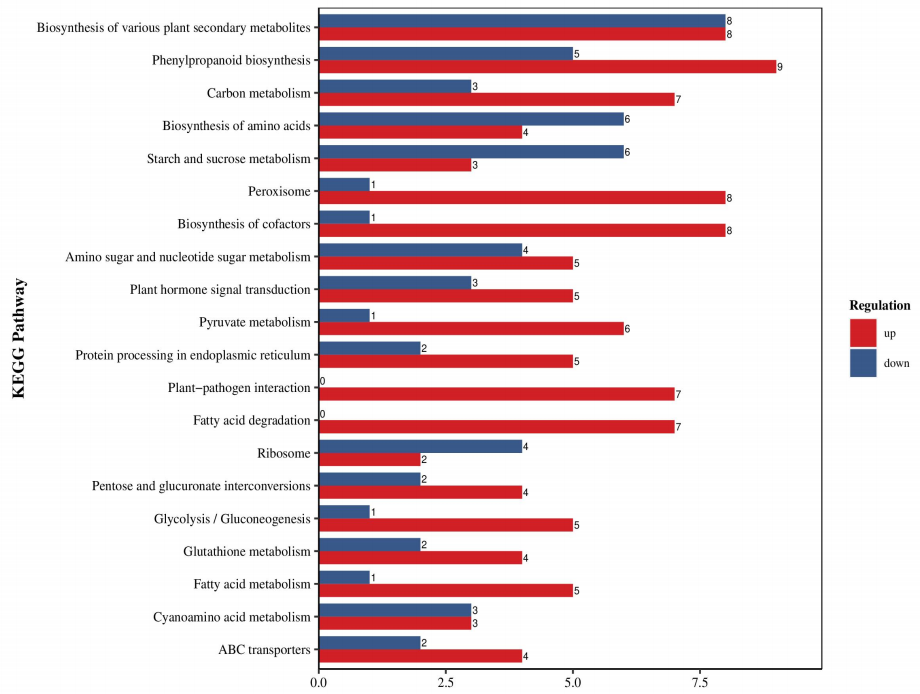


**Fig. 6** Bar chart of KEGG classification for up- and down-regulated DEPs
